# High-throughput method for detection and quantification of lesions on leaf scale based on trypan blue staining and digital image analysis

**DOI:** 10.1186/s13007-020-00605-5

**Published:** 2020-05-04

**Authors:** Emina Mulaosmanovic, Tobias U. T. Lindblom, Marie Bengtsson, Sofia T. Windstam, Lars Mogren, Salla Marttila, Hartmut Stützel, Beatrix W. Alsanius

**Affiliations:** 1 Department of Biosystems and Technology, Microbial Horticulture Unit, Swedish University of Agricultural Sciences, PO Box 103, 23053 Alnarp, Sweden; 2 Department of Crop Production Ecology, Plant Ecology Unit, Swedish University of Agricultural Sciences, PO Box 7043, 75007 Uppsala, Sweden; 3 Department of Plant Protection Biology, Chemical Ecology Unit, Swedish University of Agricultural Sciences, PO Box 102, 23053 Alnarp, Sweden; 4 Department of Biological Sciences, State University of New York at Oswego, 7060 NY-104, Oswego, NY 13126 USA; 5 Department of Plant Protection Biology, Resistance Biology Unit, Swedish University of Agricultural Sciences, PO Box 102, 23053 Alnarp, Sweden; 6 Institute of Horticultural Production Systems, Gottfried Wilhelm Leibniz University Hannover, Herrenhäuser Str. 2, 30419 Hannover, Germany

**Keywords:** Damage, Image analysis, Leaf scale, Leafy vegetables, Lesions, Spinach, Wounds

## Abstract

**Background:**

Field-grown leafy vegetables can be damaged by biotic and abiotic factors, or mechanically damaged by farming practices. Available methods to evaluate leaf tissue damage mainly rely on colour differentiation between healthy and damaged tissues. Alternatively, sophisticated equipment such as microscopy and hyperspectral cameras can be employed. Depending on the causal factor, colour change in the wounded area is not always induced and, by the time symptoms become visible, a plant can already be severely affected. To accurately detect and quantify damage on leaf scale, including microlesions, reliable differentiation between healthy and damaged tissue is essential. We stained whole leaves with trypan blue dye, which traverses compromised cell membranes but is not absorbed in viable cells, followed by automated quantification of damage on leaf scale.

**Results:**

We present a robust, fast and sensitive method for leaf-scale visualisation, accurate automated extraction and measurement of damaged area on leaves of leafy vegetables. The image analysis pipeline we developed automatically identifies leaf area and individual stained (lesion) areas down to cell level. As proof of principle, we tested the methodology for damage detection and quantification on two field-grown leafy vegetable species, spinach and Swiss chard.

**Conclusions:**

Our novel lesion quantification method can be used for detection of large (macro) or single-cell (micro) lesions on leaf scale, enabling quantification of lesions at any stage and without requiring symptoms to be in the visible spectrum. Quantifying the wounded area on leaf scale is necessary for generating prediction models for economic losses and produce shelf-life. In addition, risk assessments are based on accurate prediction of the relationship between leaf damage and infection rates by opportunistic pathogens and our method helps determine the severity of leaf damage at fine resolution.

## Introduction

Plant lesions (damage) are localised areas of dead cells on plant surfaces, typically occurring due to disease or trauma, such as wounding. Leafy vegetables are exposed to a diverse array of stress factors throughout pre- and post-harvest handling that can cause wounding. Such factors can be biotic (plant pathogens and insects), abiotic (e.g. wind, hail, drought, sunburn, freeze injury, nutrient imbalance) or mechanical (originating from agricultural and processing practices). As leaves are the principal site for photosynthesis, they are essential for plant survival. Cellular injury results in loss of water and solutes from the damaged area [1, 2] and localised cell death, causing loss of chlorophyll and thereby reduced net photosynthetic rate [3], affecting plant growth and metabolism [4]. Wounding leads to physical damage of cell membranes, disrupting both their function and the function of neighbouring cells [5]. Depending on the severity of damage, wounding can cause abnormal growth of plant organs and result in decreased crop productivity and yield [6]. In addition, injury-related leached solutes on the leaf surface provide nutrients that can support prolonged survival of microbial pathogens [1], making injury sites preferred habitats for microorganisms [7]. Injury sites can also serve as ports of entry for opportunistic bacterial pathogens [8] that lack the ability to break down pectin, allowing invasion of intact leaf tissues. Because most opportunistic pathogen cells are smaller in size than plant cells, methods for detection and quantification of microscopic, single-cell tissue damage on leaf scale are needed. Such methods would be very useful in research and also within the processing industry for leafy vegetables.

It is necessary to discriminate between damage that is manifested in the visible spectrum and damage without visible symptoms. Most current methods for evaluating plant damage, including damage due to plant diseases with visible symptoms, are based on visual assessment by trained experts. Such methods are laborious, time-consuming and prone to error, bias or optical illusions, and the precision decreases with rating time and when visual symptoms are small in size and abundant in number [9, 10].

The agriculture sector has expressed interest in replacing this mostly manual process with more automated, objective and sensitive approaches, such as digital (RGB), multispectral and hyperspectral imaging [11–13] and digital image processing [14–18]. Multispectral and hyperspectral imaging is a rather new, non-destructive but expensive technology, and generates large amounts of data that are sometimes difficult to collate and process [19]. Detection of single-cell injuries is challenging and currently based on microscopy, which is not an adequate tool for quantification of damage on leaf scale. An automated assessment such as RGB digital image analysis is faster, increases throughput, reduces subjectivity and is highly repeatable [14].

Digital image processing approaches are useful for detection, quantification and classification of plant pathologies [14, 17, 20–22], and measurement of plant disease severity [19, 23] in an objective manner. The basic approach for image processing techniques includes image pre-processing, segmentation, feature extraction, feature selection and classification of the diseased areas or leaves. Detailed surveys of established image processing techniques used for automated detection and classification of lesions have been reported [18, 21, 24–26]. Considering damage, those approaches employ an array of lesion segmentation and classification techniques such as thresholding [27, 28], edge detection [29, 30], watershed [31], fuzzy c-means [32], superpixel clustering [33], color transformation [17], pixel classification [22], improved histogram segmentation method [34], and genetic algorithms [14]. Popularly used classification techniques for plant lesion identification are K-means [35], K-nearest neighbor [36], Artificial Neural Networks [37, 38], Support Vector Machine [39–41], and Deep Learning [42–47] as a new standard in digital image analysis. Due to the complexity and variation of lesion symptoms, and as the color of normal region and lesion region is also uneven and unclear [48], segmentation of lesions in an image is challenging. In attempt to overcome these challenges, some damage detection and classification approaches are based on combination of techniques, such as local threshold and region growing [49], auto-cropping segmentation and fuzzy c-means [50], super-pixel clustering with K-mean clustering and pyramid of histograms of orientation gradients algorithms [51], and Markov Random Field combined with edge detection [52], and more.

A reduction in the accuracy of disease severity estimation algorithms has been reported [9] when the contrast between healthy and damaged tissue is low. Successful image segmentation relies on a sharp contrast between healthy (green) and damaged (yellow or brown) tissue, therefore infection needs to reach a threshold disease severity level in order to be detected. Furthermore, available damage detection methods based on image processing are developed for multiple cell (macroscopic) lesions [14, 20], and lower limit of detection with regards to lesion size is seldom discussed, except for stating the efficacy of proposed algorithms in detection of small lesions [20, 22]. Such a discussion needs to take place as the size of lesions is crucial for early detection of plant diseases and their controlling.

Leafy vegetables are mechanically damaged by cutting and bruising throughout harvest and post-harvest handling. Mechanical damage does not cause changes in tissue colour as such and there is no visible contrast between damaged and undamaged tissues. Thus, the level of mechanical damage can only be manually assessed. For mechanical damage and early stage damage detection, preceding a colour change, methods relying on natural colour change are invalid. Hence use of image processing quantification methods necessitates enhancing the contrast between healthy and damaged leaf tissue. In the leafy vegetable processing industry, there is a need for a robust and rapid method for evaluating produce quality, including damage quantification.

Trypan blue (TB) is a specific dye used for detection of dead plant tissue [53–55]. Staining with TB enables colour discrimination between intact-viable and damaged cells [56]. Intact cells exclude the dye, whereas cells with damaged membranes are stained blue [56], enhancing the contrast between intact and damaged tissue. TB has been used for discriminating structures on leaf surfaces, as it also stains chitin in fungal cell walls [57, 58], and has been used extensively in plant pathology for studying plant-fungal interactions by microscopy [53, 59–61].

To the best of our knowledge, there is no existing method for detection and automated quantification of damage to leafy vegetables on leaf scale that combines TB staining with digital image analysis.

The aim of this work was to develop a robust method for (i) detection of multiple (macro) and single-cell (micro) lesions on leaf scale and (ii) automated quantification and classification of lesion parameters using diverse established digital image processing methods combined with TB staining. Novelty of the proposed approach is in its simplicity achieved by combining clearing and TB staining with an array of widely employed image processing techniques such as OTSU and local thresholding, and DBSCAN and K-means clustering algorithms, enabling the proposed approach to distinguish healthy and damaged areas down to single cell level correctly.

## Materials and methods

Spinach (*Spinacia oleracea*) and Swiss chard (*Beta vulgaris* subsp*. vulgaris*) were chosen as sample leafy vegetables when developing the high-throughput method. All plants were grown outdoors in southern Sweden in June 2017 under conventional farming practices for 4 weeks (Vidinge Grönt, AB). Individual leaves were manually harvested at baby-leaf stage (BBCH stage 13) and transported to the laboratory in plastic containers (Orthex Sweden AB; 50 cm × 39 cm x 26 cm) to avoid additional damage.

**Fig. 1:**
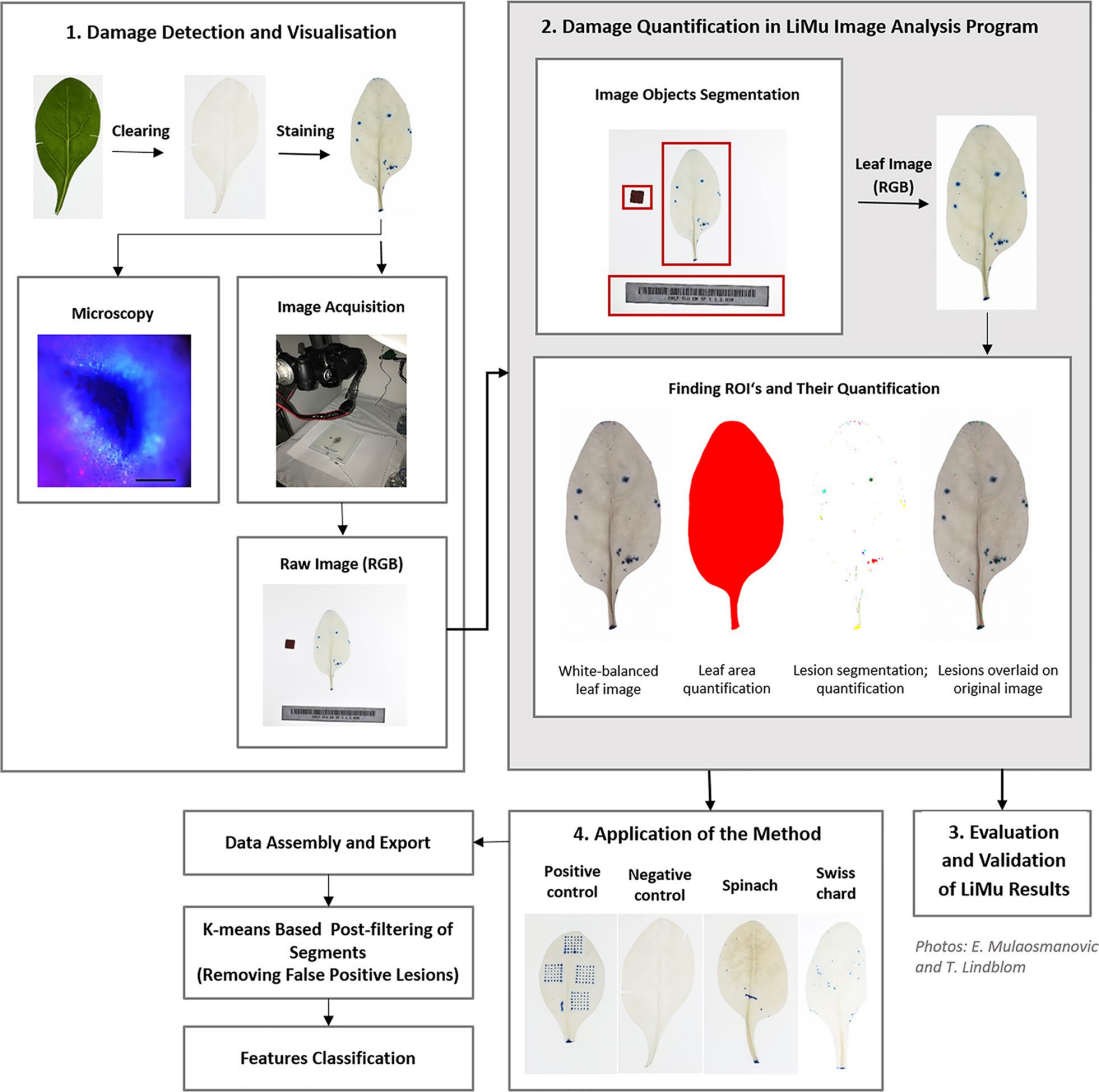
Overview of the lesion detection and quantification method. There are two main steps (1) damage detection and visualisation by clearing and staining and subsequent microscopy verification of staining (scale bar 50 µm) and image acquisition; and (2) damage quantification using the LiMu image analysis program. LiMu program results were (3) evaluated by comparing them against results acquired with IMAGEJ software and manual assessment, and validated with IMAGEJ results, followed by (4) application of the method on an experimental dataset

The experimental procedure comprised two main steps (1) damage detection and visualisation, and (2) damage quantification using the LiMu image analysis program (Fig. 1). LiMu results were (3) compared against results acquired with IMAGEJ software and manual assessment, and validated with IMAGEJ, followed by (4) application of the method on an experimental dataset.

### Damage detection and visualisation

Detection and visualisation of lesions was performed on detached, whole leaves in a series of steps comprising clearing, staining and verification of staining by microscopy and RGB image acquisition.

#### Clearing protocol

To enhance the contrast between healthy and damaged (later stained) tissue, chlorophyll was removed from the tissue of detached leaves by soaking the whole leaves in a clearing solution composed of ethanol (Solveco, 95%) and acetic acid (Acros Organics, 99.6%) in a 3:1 (v/v) ratio. Similar clearing protocols are described by Schenk and Schikora [62] and Sharma [63]. All leaves were soaked in clearing solution until they became entirely transparent, usually overnight (15–17 h) on a rotary shaker (50 rpm) at room temperature (21 °C). Saturated clearing solution was replaced when necessary. Clearing was followed by washing in 50% ethanol for 15 min on a rotary shaker (50 rpm).

#### Staining protocol

The staining step to detect and visualise leaf tissue damage was based on an existing protocol [59], but with modifications to TB concentration and staining time. In brief, whole cleared leaves were incubated in 0.01% TB (Sigma-Aldrich) in de-ionised water (diH_2_O) for 4 h on a rotary shaker (50 rpm), followed by washing with diH_2_O until the wash-off water was clear.

#### Verification of staining

To verify that only damaged areas were stained with TB dye, samples were studied using an inverted fluorescent microscope. First, leaves were artificially damaged with a Derma stamp (HudRoller Of Sweden; 36 microneedles; 1 mm) and Derma roller (HudRoller Of Sweden; 1 mm), mimicking dot-like lesions. Damaged leaf samples were immediately cleared. To achieve better visualisation of lesions, samples for microscopy were subjected to dual staining with TB and aniline blue (AB) dyes (Acros Organics). Aniline blue stains callose [59–62], including trauma-induced callose deposited around lesions. Cleared leaves were first soaked in 0.01% TB staining solution in diH_2_O (4 h), washed in diH_2_O, and stained with 0.01% AB staining solution (2 h) in 150 mM K_2_HPO_4_ (Merck) [62], followed by washing in 150 mM K_2_HPO_4_. Leaf discs (Ø 10 mm) were then extracted from corresponding sites on TB-stained and dual-stained leaves (N = 5), using a coring tool (Harris Uni-Core). As a negative control for microscopy, discs from cleared leaves without the staining step were imaged (N = 5). Microscopy was carried out using a Zeiss Axio Observer D1 microscope (Carl Zeiss, Jena, Germany) as described previously [62].

#### Image acquisition

Stained leaves were placed on a LED light table (DÖRR GmbH; 200 × 200 × 8 mm), with a few drops of water between light table surface and sample, and a barcode label was added. The barcode specified the year, plant species, experimental repetition, replicate and sample number of the leaf. A reference standard-size object (1 cm^2^) was included in each image. A camera (Canon EOS 5D Mark IV fitted with a Canon EF 50 mm 1:1.4 lens) was placed vertically on a tripod, at a height of 35.5 cm above the sample, and operated in manual exposure mode (shutter speed 1/125, aperture 6.3, ISO 160). The height and objective magnification were adjusted for the field of view to include the LED light table, with leaf, sample barcode and standard-size object. The focus of the camera and its distance from the sample remained fixed for the duration of the experiment. Images were obtained in a dark room, with light only from an LED light table below the sample. Images were collected in raw format (CR2), with picture dimensions 6880 × 4544 (31.26 MP) and approximate size 62–65 MB.

*Preparation of image datasets*: The *original* image dataset consisted of stained spinach (N = 300) and Swiss chard (N = 300) leaf images, negative control images (N = 25) representing undamaged leaves (cleared, unstained leaves and positive control images (N = 36) with different severity levels (low to high) of standardised artificial damage (cleared and TB-stained leaves).

The original dataset was divided into two subsets: (1) a *primary* image dataset, used to design the image analysis pipeline, and (2) a *test or experimental* image dataset, used to evaluate the pipeline developed.

The primary image dataset comprised randomly selected images of spinach leaves (N = 100) from the original dataset. Selected images represented the range of plant damage expected to be encountered in the original image set.

The experimental image dataset consisted of the remaining images of spinach leaves (N = 200) and randomly selected images of Swiss chard leaves (N = 200) from the original dataset, along with control images.

### Damage quantification in LiMu image analysis pipeline

**Fig. 2:**
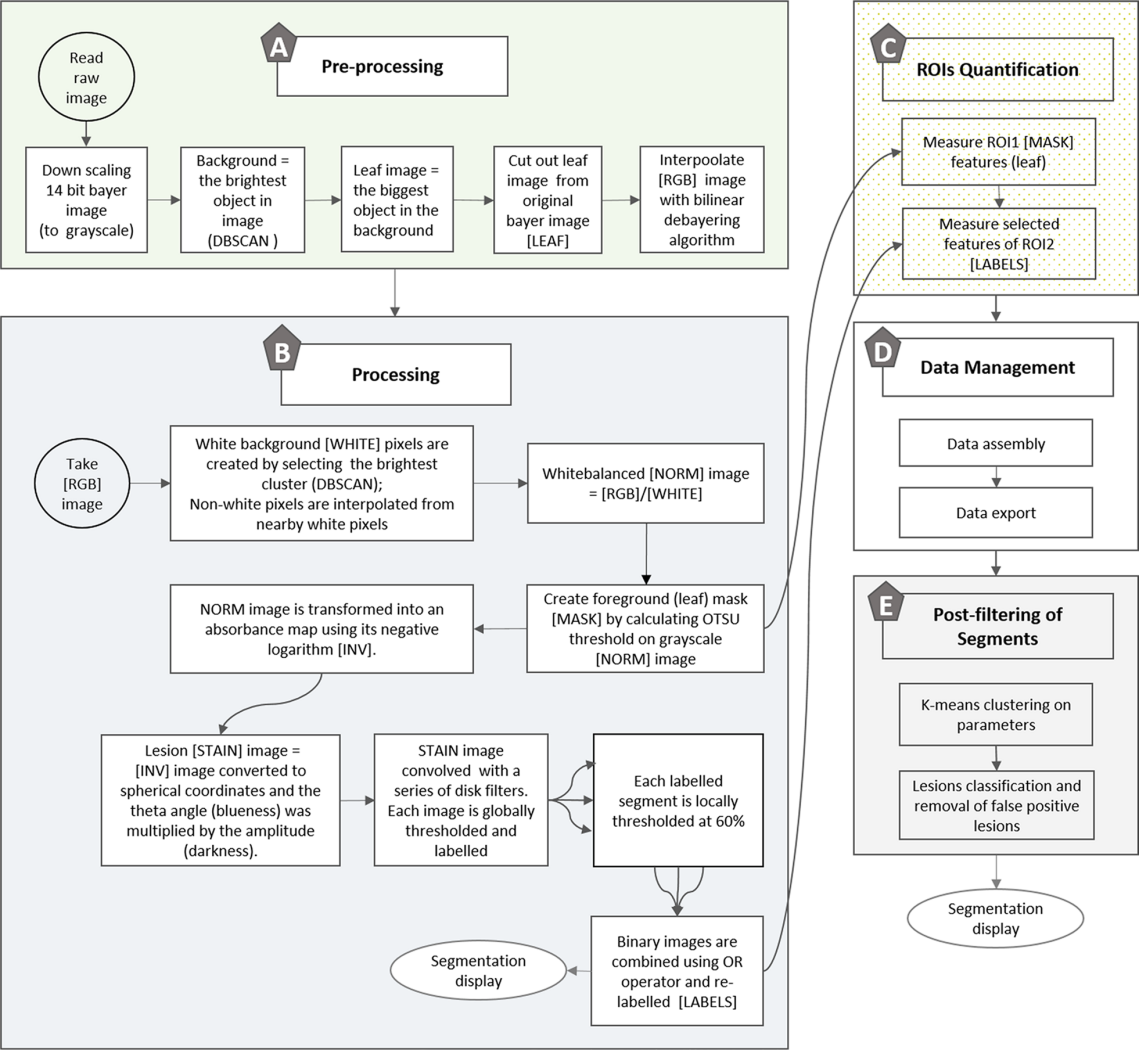
The LiMu image analysis pipeline. Detailed schematic overview of the main steps in the LiMu image analysis program: **a** Image pre-processing, **b** image processing, **c** quantification of regions of interest (ROIs), **d** data management and **e** post-filtering on segments with K-means clustering

The LiMu image analysis program is written in Python and its main objectives are to identify and quantify leaf area and individual lesion (stained) areas and their morphometric parameters (Fig. 2). The current application of the program consisted of (1) image *pre*-*processing* (Fig. 2a), i.e. finding leaf images, (2) *processing* (Fig. 2b), i.e. finding and segmenting regions of interest (ROIs, where ROI1 = leaf, ROI2 = lesion), (3) *quantification* of ROIs (Fig. 2c), (4) *data management* (Fig. 2d) and (5) *post*-*filtering of segments* (Fig. 2e). The LiMu image analysis program enables segmentation of leaf and leaf lesions and quantification of total leaf area and of individual lesion areas and their morphometric parameters. The LiMu script for automated image processing of stained spinach leaves is provided in Additional file 1.

#### Pre-processing

Unprocessed raw (Canon, CR2) 14-bit, Bayer-encoded image data were imported using the rawkit library [64]. To increase processing speed, image data were downscaled by a factor of 16 and pixel (px) values were linearly scaled between 0 and 1. In order to extract morphometric plant features from images, leaf regions (foreground) were segmented from the light table (background).

*Background segmentation:* To select the brightest object in image, representing the light table (background), non-linear intensity correction was conducted, followed by clustering on pixel indices using the hdbscan library [65]. Pixels belonging to the largest cluster were identified as the backlit light table.

*Leaf image segmentation:* Once the background was identified, the program was used to search for objects (holes) in the background, and the identified objects were classified as “leaf image”, “size marker image” (if present) and “barcode image”. The largest logical hole in the background was assumed to be the leaf image, which was extracted with surrounding background [LEAF] from the full resolution Bayer image. Leaf images were de-Bayered using a bi-linear de-mosaicing algorithm into a colour image [RGB].

#### Processing

The colour images [RGB] obtained consisted of leaf and leaf surrounding background (Fig. 1).

*Leaf segmentation* (*ROI1*): White balancing for nearly white background images [WHITE] was performed by creating a crude foreground/background mask and clustering pixel indices and colour intensities of the colour [RGB] image with DBSCAN [66]. The background area was set to the largest area object. The second largest area object was the designated foreground (leaf). The white image representing the light-table (background) was re-constructed using the background and infilling by means of linear interpolation. Foreground non-white pixels (leaf) were constructed using data from the nearby white pixels (background). A white-balanced/normalised image [NORM] was created by dividing the RGB image by the WHITE image. The foreground mask [MASK] was made using OTSU thresholding on a grey scale version of the white-balanced image. This mask also contains information on actual holes in leaves, when present.

*Lesion segmentation* (*ROI2*): To retrieve absorbance information (blueness), the white-balanced image was transformed into an absorbance map using its negative logarithm [INV]. The inverted image [INV] was then converted to spherical coordinates and the Theta angle (blueness) was multiplied by the amplitude (darkness). This resulted in a lesion [STAIN] image representing the lesion area. This lesion image was convolved with a series of disc filters with radius 1–13 pixels, constructed with outside and inside disc pixels adding up to zero. The resulting 13 images were thresholded at 0.1 of their maximum value. These binary images were dilated once and logical holes in individual segments (lesions) were filled and labelled. Each labelled segment was then thresholded at 60% of the stain value range within the segment. The thresholded segments were combined using logical operator (OR) to a single binary image and relabelled [LABELS].

#### Quantification

The ROIs leaf area (ROI1) and individual stained areas (lesions, ROI2) were quantified. Leaf area was measured from the foreground (leaf) mask and lesion area was measured from combined binary images. Morphometric parameters, e.g. area, Theta angle (blueness), location (distance from edge and from centreline), shape, perimeter and amplitude (darkness), were measured for each labelled segment representing a single lesion. These parameters were then selected as data features, representing the raw dataset, saved as a text file and used for further evaluation.

#### Post-filtering of segments

Post-filtering of segments, an optional step in the LiMu program, was performed on extracted data points in order to remove false positive lesions introduced as a result of uneven clearing between different leaves, but also between different parts of the leaves used in the experiment. This step can be customised based on the research question, e.g. parameters used for clustering (shape descriptors, distance from the edge and centre of the mask) can be added or removed. Lesion (segment) data from all segmented images were post-filtered and classified into 30 clusters using K-Means (Minibatch), with each individual lesion in the text file assigned a number from 1 to 30. Features used for clustering were area, maximum Theta angle (blue value), maximum to minimum Theta angle (range), maximum amplitude (dark value), maximum to minimum amplitude (range), eccentricity and log_10_[Square root of area + 0.1]. Clusters were plotted (10 × 15) by randomly selecting 225 lesions (segments) with the same class number assigned. Visual inspection was used to determine which of the clusters contained false positive lesions, followed by automated removal of individual lesions that had a class number assigned in the text file. Figures containing plotted clusters (classes) are provided in Additional file 2. After false positive lesions had been removed, lesions were once again displayed on images. Images used to create the image analysis program were not used for validation.

### Evaluation and validation of the LiMu program

To evaluate potential benefits of the LiMu program application developed in the present study, the results obtained with LiMu were compared against results obtained with two commonly used approaches: (i) automated processing in IMAGEJ, regarded as the discipline-standard image analysis system, and (ii) manual segmentation (manually rounding lesion areas with the “freehand” tool in IMAGEJ). Images used for comparisons of results obtained with these three approaches were positive control images (N = 10) and randomly selected test spinach images (N = 10).

For image analysis in IMAGEJ, a macro was written (Additional file 3). Due to large variations in the colour of cleared leaf tissue and the size and intensity of the stained areas, the default thresholding algorithm in IMAGEJ could not be successfully applied uniformly across the entire dataset. It was found that the Shanbhag thresholding algorithm [67] provided the best lesion segmentation on the largest number of the images. Thresholding was performed on the red channel. There were two major differences in the image analysis workflows between the two tools. First, leaf image segmentation in LiMu was completely automated, while in IMAGEJ leaf images had to be manually cut from the pictures and saved for later batch processing, as the position of the leaf on the light table varied. Second, post-filtering as a form of error correction step where false positive detected lesions were removed was lacking in the IMAGEJ workflow (Additional file 4).

To validate LiMu image analysis results, a simple linear regression analysis was carried out using the results of LiMu as the explained variable and the results of IMAGEJ as the explanatory variable. Validation was performed on 50% of the experimental dataset, containing both spinach and Swiss chard leaf images.

### Application of the method on the experimental dataset

The LiMu program was applied on the experimental image dataset, which was processed in the same manner as the primary dataset used to create the image analysis program.

### Statistical analysis

Statistical analysis was performed in R studio (version 3.6.1.) [68], using packages *ggplot2* for plotting and *ggpubr* for customisation in ggplot2 plots. Differences in damage level means for leaf area, lesion area, number of lesions per leaf and leaf damage (%) were tested with the nonparametric Kruskal–Wallis test. Pairwise multiple-comparison post hoc tests were carried out using Dunn′s test, with Holm correction to adjust the significance values for multiple comparisons. Differences in means between the LiMu image analysis program, the image analysis software IMAGEJ and the manual assessment approach in terms of leaf area, lesion area, leaf damage, lesion number and lesion classes were tested with the nonparametric Friedman′s test, with image used as block, followed by Dunn′s post hoc test. Function *geom_hline*() was used to add y-intercept. A linear regression model was created using the *lm*() function. Function *geom_smooth*() was used to add regression lines to scatter plots and a reference line with slope = 1 and intercept = 0 was added using the *geom_abline* () function. Coefficient of determination (R^2^) was calculated using *stat_cor*() function. The linear regression models used for linear regression analysis were $y_{LiMu} = \beta_{0} + \beta_{1} x_{ImageJ}$ and $y_{LiMu} - x_{ImageJ} = \beta_{0} + \beta_{1} x_{ImageJ}$. Differences in mean values of morphometric variables between the two leafy vegetable species were tested with the nonparametric Wilcoxon test. A two-dimensional density estimation was added to the scatter plot using the *geom_density_2d*() function.

## Results and discussion

### Method development and associated issues

**Fig. 3:**
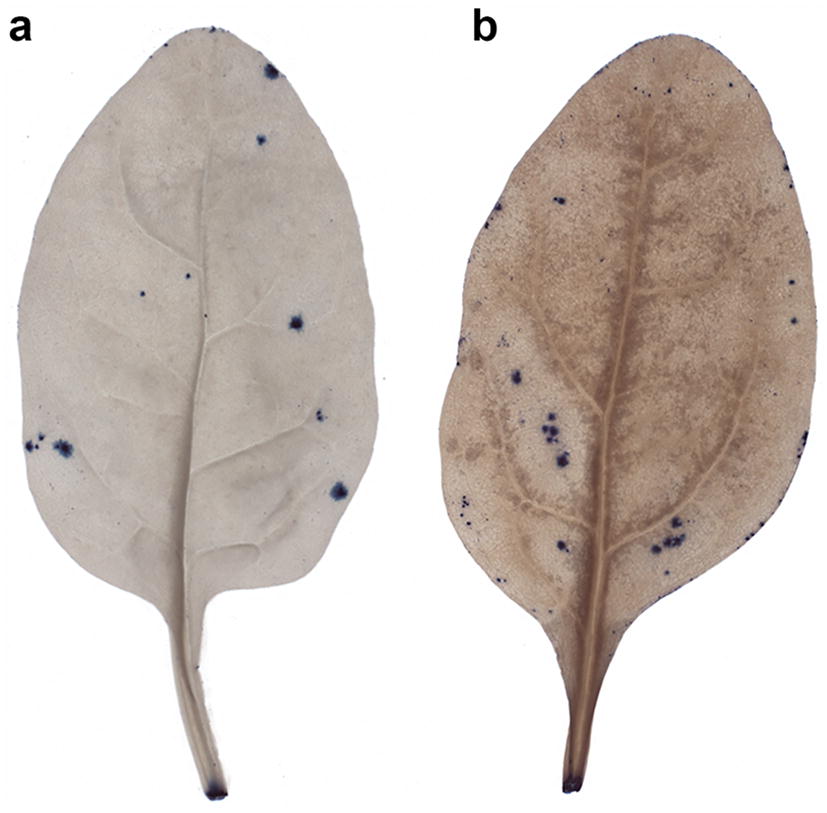
Examples of **a** even and **b** uneven (incomplete) leaf clearing. Leaf images acquired post-staining with trypan blue dye

**Fig. 4:**
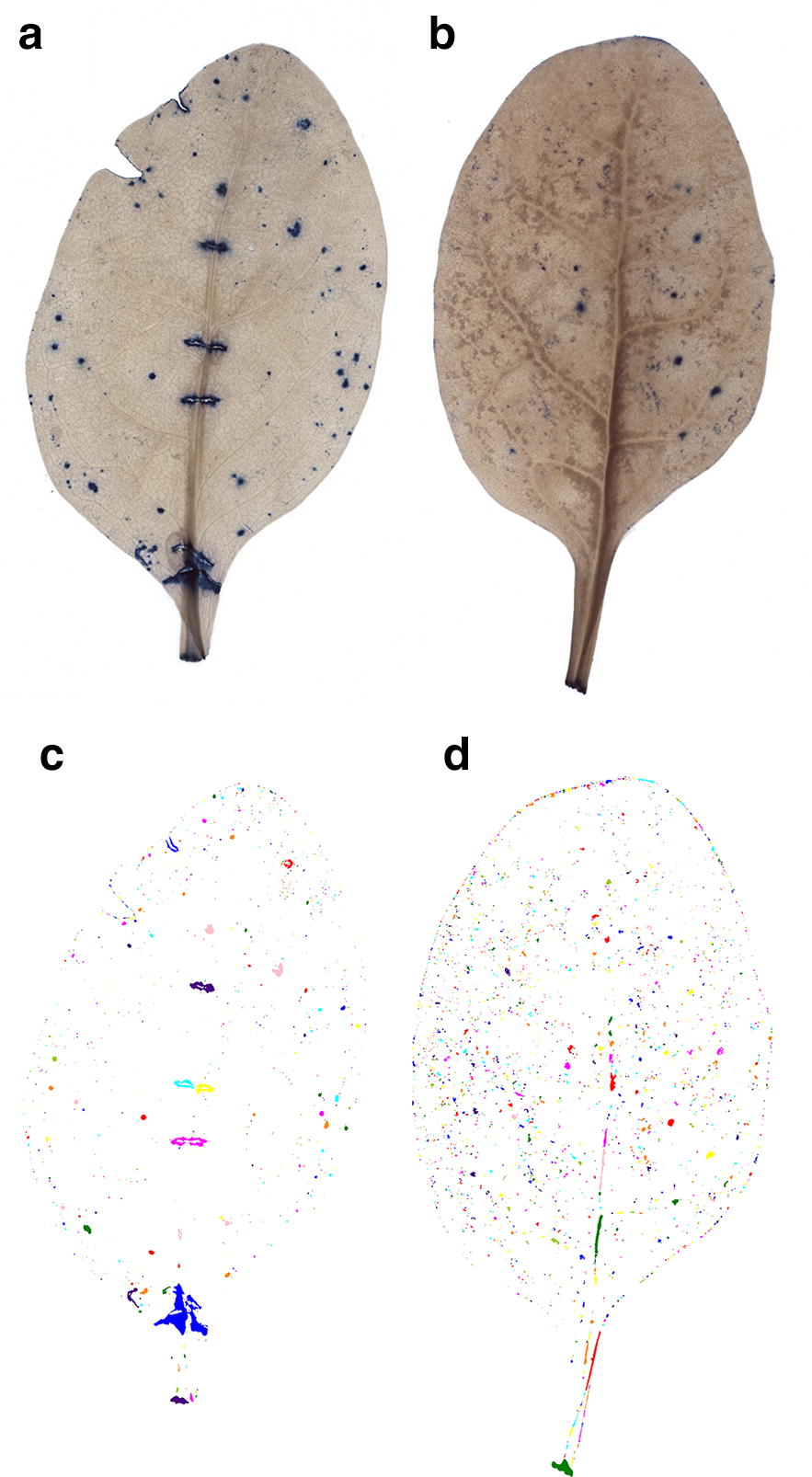
Examples of leaf lesion segmentation using the LiMu program. Images of leaves with **a** even and **b** uneven tissue clearing, acquired post-staining with trypan blue dye. **c**, **d** Labelled lesion segments from leaf images **a** and **b**. Uneven or incomplete clearing (**b**) can result in subsequent detection of false positive lesions (**d**) in poorly cleared leaf parts

**Fig. 5:**
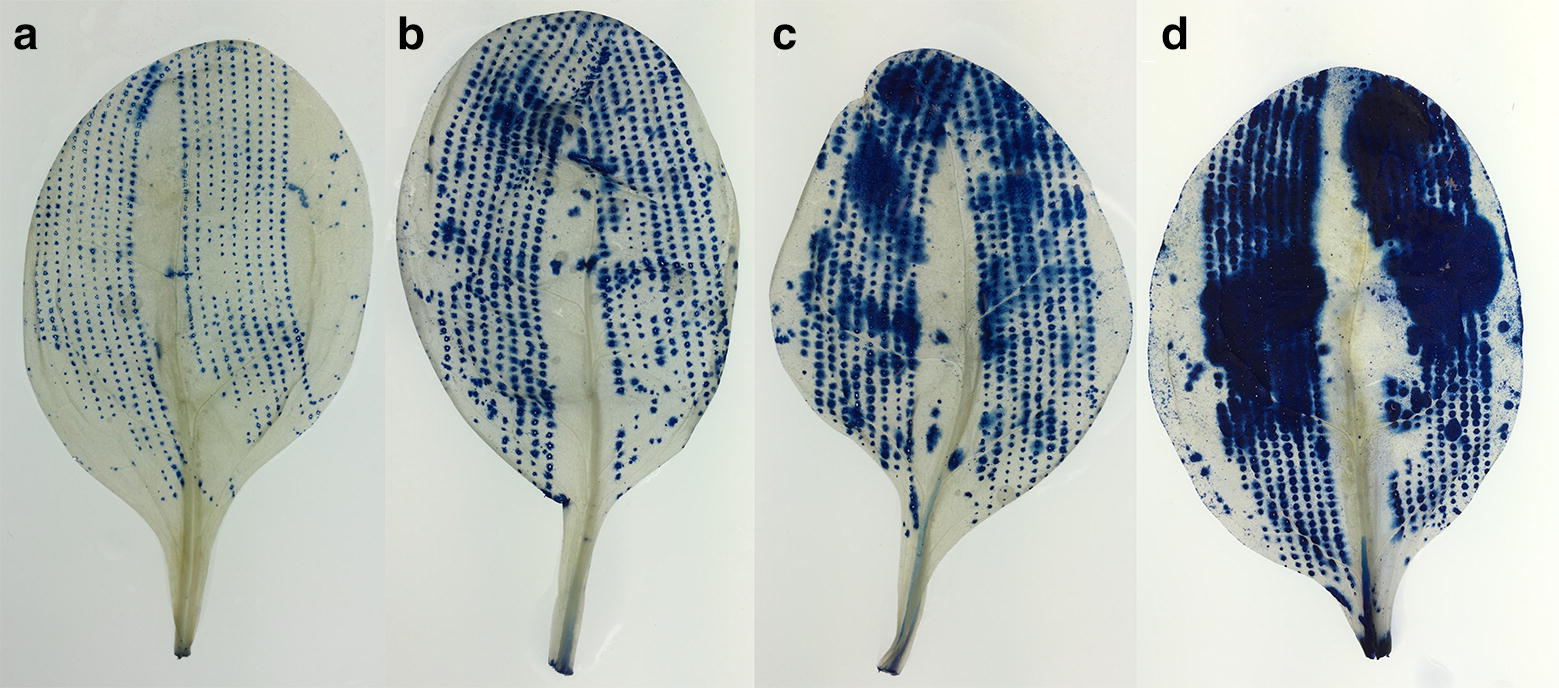
Examples of artificially damaged leaves, stained post-clearing with different concentrations of trypan blue (TB) dye. Leaves were stained with either **a** 0.01% TB, **b** 0.05% TB, **c** 0.1% TB, or **d** 1% TB

**Fig. 6:**
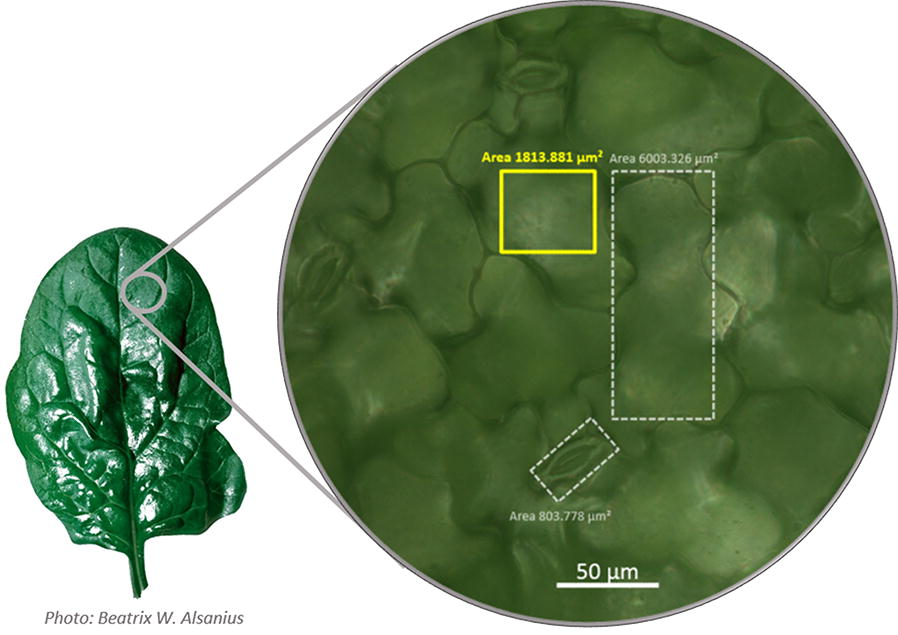
Micrograph of spinach epidermal cells. Area within the yellow square (≈ 1800 µm^2^) corresponds to the size of one pixel in spinach photographs analysed using the LiMu program and represents the smallest area (bottom threshold) that can be detected and quantified with LiMu. This area is approximately two times larger than a stomata (small white rectangle), and three times smaller than an average spinach epidermal cell (large white rectangle)

**Fig. 7:**
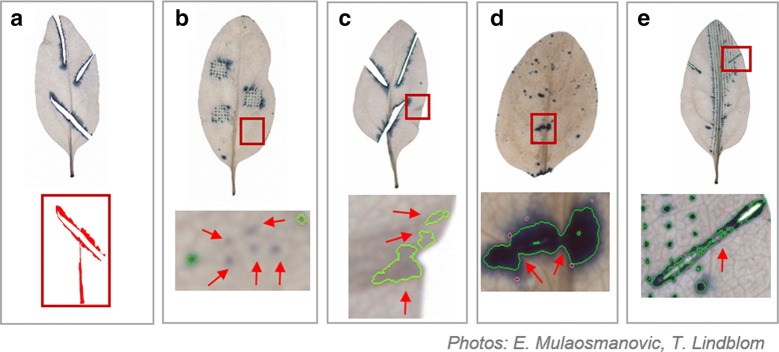
Examples of object mis-segmentation (red squares) after processing using the LiMu program. Common types of mis-segmentation: **a** Failure to segment leaf area, **b** failure to detect lesions (false negative lesions), **c** recognition of undamaged plant tissue as lesion (false positive lesions), **d** recognition of two or more neighbouring lesions as one larger lesion (under-segmenting), and **e** recognition of one lesion as two or more lesions (over-segmenting)

**Fig. 8:**
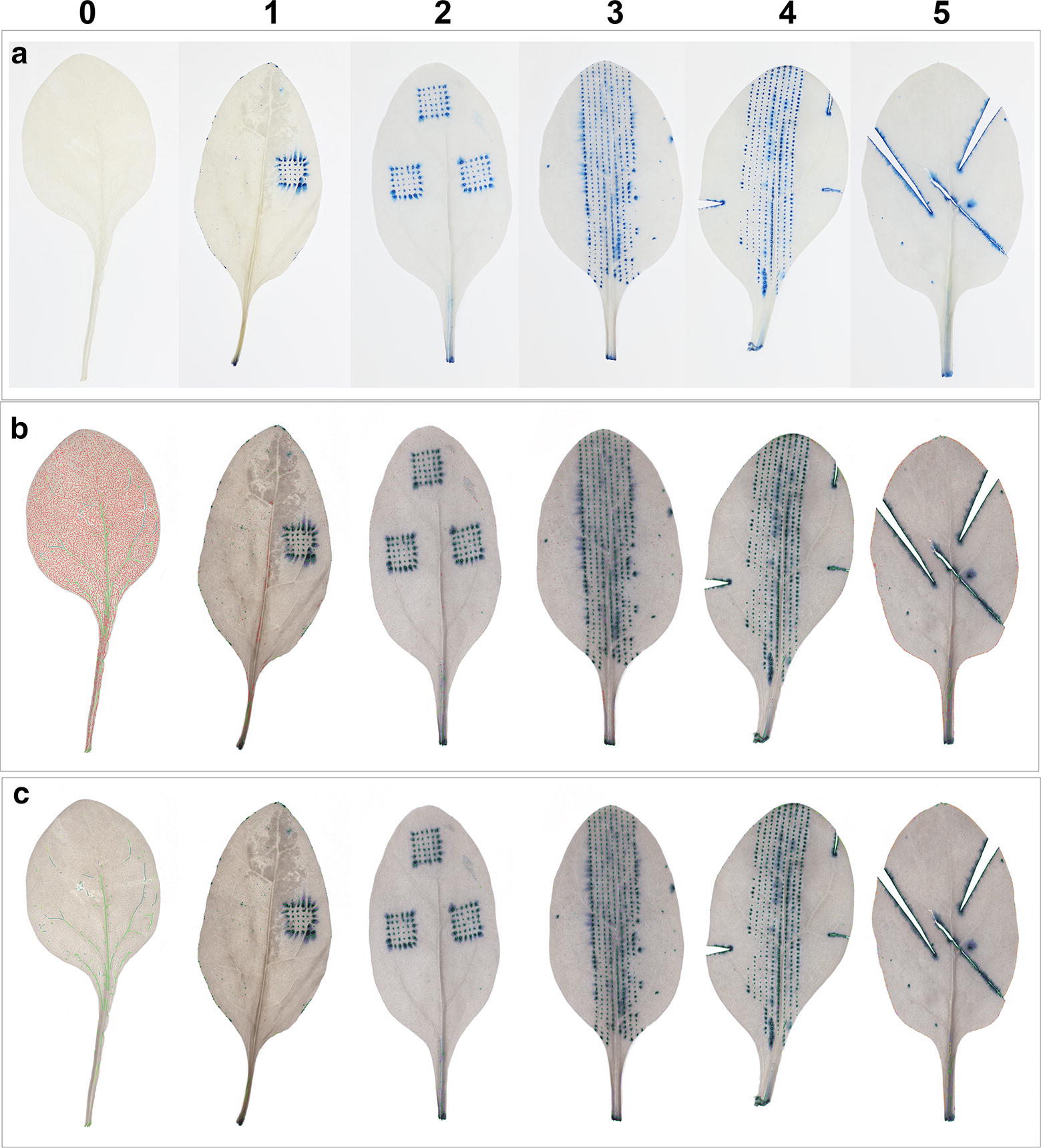
Different levels (0–5) of standardised artificial damage inflicted on spinach leaves. Leaf images **a** before and **b** after processing in the LiMu program, and **c** after K-means based filtering and removal of false positive segments

**Fig. 9:**
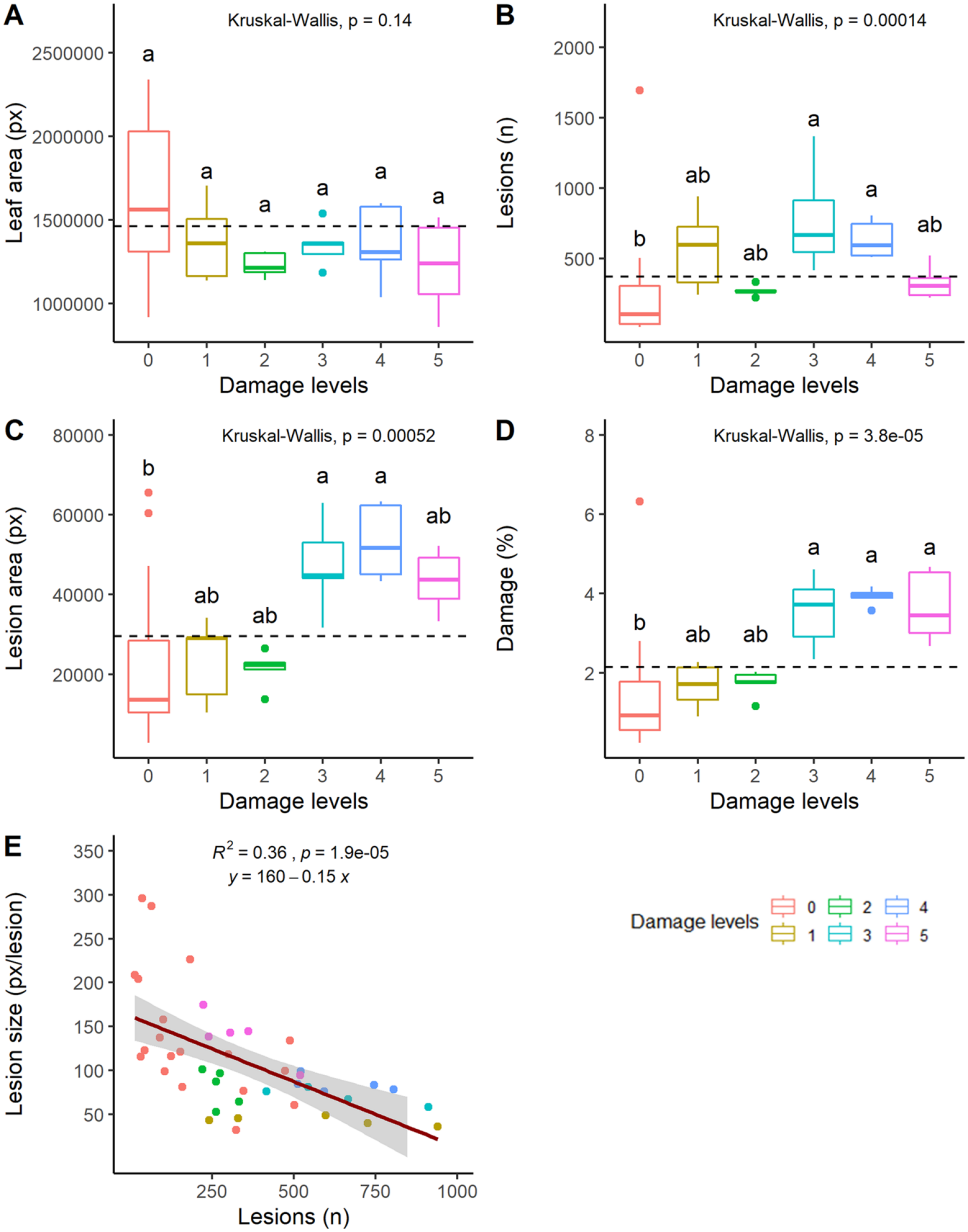
Leaf morphometric parameters for different levels of standardised artificial damage (0–5) inflicted on spinach leaves. Results shown are average values for negative (N = 25) images and each damage level for positive (N = 5) images. The morphometric parameters **A** leaf area, **B** number of lesions, **C** lesion area and **D** leaf damage (%) were compared. Area was measured in pixels (px) and leaf damage was calculated as $Damage = \left( {\frac{lesion area}{leaf area}} \right) \times 100$. Dashed horizontal line represents the overall mean across damage levels. A nonparametric Kruskal–Wallis test was used, followed by Dunn′s post hoc test. Significant differences (p ≤ 0.05) identified in the post hoc test are indicated by different lower-case letters (a, b) (Additional file 7). **E** Results of linear regression analysis between number of lesions and lesion size

We successfully developed a robust, cost-effective and fast method for detection and quantification of lesions on leaves of leafy vegetables, which can be handled in full by one person (Fig. 1). The infrastructure required is commonly available in most laboratories and comprises a balance, fume hood, shaker, chemicals, tripod-supported high-resolution camera, light table and computer (Additional file 5). During development of the method, we identified three steps that generally caused the majority of issues in the process, namely clearing of leaf tissue (Figs. 3, 4), detection of damage by TB staining (Figs. 4, 5, 8a) and quantification of damage in the LiMu program (Figs. 2, 6, 7, 8, 9, 11A, B).

*Clearing of leaf tissue*: Clearing involves removal of chlorophyll from leaf samples in order to provide sufficient contrast between TB-stained and intact tissue. Leaf samples can be cleared and stained individually, or batch-processed. To enhance penetration of clearing and staining solutions, careful shaking during clearing is recommended. We discovered variations in clearing outcome between different leaf samples and between different parts of the same leaf, due to differences in size and thickness (Fig. 3). It took longer for the clearing solution to penetrate large/thick leaves and evenly clear the leaf midrib surrounding area. Complete clearing of all leaf parts was critical in achieving accurate feature segmentation in the LiMu program (Figs. 3, 4). The volume of the clearing and staining solutions must be adjusted to the amount of leaf samples processed and to leaf area and thickness. There should be enough solution to completely cover all leaf material and enable floating of leaves. Insufficiently cleared leaf areas, found mainly along the midrib and its surrounding area, were falsely recognised as lesion tissue in the damage quantification step (Fig. 4). We optimised the protocol for spinach and for Swiss chard, which has red leaf ribs. For other plant species, the clearing protocol would need to be optimised for the leaf morphology and properties of the particular species. This can be achieved by (i) increasing the overall volume of clearing solution added to leaves, (ii) replacing the saturated clearing solution with fresh solution or (iii) increasing the proportion of acetic acid in the solution, before proceeding with staining.

*Detection of damage by TB staining*: Different concentrations of TB dye (0.01%, 0.05%, 0.1%, 1%) were tested, to determine the optimal concentration for staining. At high concentrations (0.1% and 1%), the dye tended to deposit on the leaf surface as precipitate, resembling staining on the intact tissue (Fig. 5), and was challenging to wash off post-staining. This led to over-estimation of damaged tissue when using image analysis for quantification. High concentrations of TB are therefore inappropriate. Dye deposition was lowest with 0.01% TB and enabled visualisation of damaged areas, providing good visualisation of damaged leaf tissue. At a concentration of 0.01%, TB dye was internalised in spinach tissue in a pattern characteristic of the induced artificial damage (Figs. 5, 8a).

Dual TB and AB staining revealed callose deposits surrounding the damage sites (Fig. 1). Microscopy revealed that intact cells did not take up the TB dye (Additional file 6). Scrutiny of TB-stained areas showed that the dye was internalised in artificially damaged epidermal cells of spinach tissue. Some diffusion of TB dye into neighbouring cells around lesions was also observed. This corroborates previous findings on increased permeability of damaged and adjacent cells impacted by mechanical damage [5].

Due to simplicity of the clearing and staining procedures herein, we believe that a device to properly stain and clear leaf tissue would be fairly easy to build. The minimum requirements for successful performance of such a device are a closed vessel system, with mild liquid mixing or agitation. Additionally, the device should comprise automated pumping of used clearing and staining solution, and rinsing of the system and samples between the two procedures, and disposal of the chemical waste. The staining procedure needs to be executed in laboratory settings, away from the processing factory facilities due to toxicity of the TB dye, feasibly within the quality control room that is common within processing facilities. Therefore, stained leaves are not be used for human consumption. Considering assessment of damage, for a proper characterization of a batch, 30 observations taken at harvest would give a statistically sound basis for decisions [69].”

*Quantification of damage in the LiMu program*: Uniformity of image acquisition and maintaining the same settings, especially light and camera distance from the object, are crucial when calculating damage as a percentage of leaf area [70]. We included an object of known dimensions (1 cm^2^) in the image, which enabled calibration of the dataset. During development of the image analysis pipeline, several image segmentation methods were tested, namely colour separation [71], superpixelation [72] and morphological snakes [73]. Colour separation worked well overall, but issues arose with very dark, almost black, stained lesions, as black has no colour. In addition, the colour of insufficiently cleared leaves (yellow–brown to green) had to be automatically assessed, which proved difficult. Superpixelation, a form of over-segmentation, worked adequately, but we could not find a robust method for reliably joining the super-pixels. The morphological snakes approach was discarded due to lack of robustness. We also tested a form of ‘local thresholding’ with square discs in the LiMu program, which proved to be the most efficient and robust solution.

Feature extraction (quantification) and data export are completely automated in the method based on the LiMu program developed in this study (Fig. 2). The program has a selective threshold, adapting to each image individually, which was an essential trait to solve the issue of variability in clearing and image quality within the dataset. It is a conservative, robust and fast program (30 s/image, depending on number of lesions per leaf). The LiMu program thereby enables large-scale image data analysis. False positive lesions are easily removed by post-filtering on data. The program is flexible to improvement and creates a framework for future analysis of damage. Depending on the research question, it is also easy to combine different extracted parameters in order to gain more information about the lesions (i.e. shape description, position, distance to edge etc.) and fine-tune lesion classification.

An example of image segmentation, where leaf and lesions are segmented from a leaf image, is shown in Fig. 1. The size of each pixel in images subjected to image analysis is approximately 1800 µm^2^. This area is demonstrated *in planta* in Fig. 6, where the area within the yellow square corresponds to one pixel in spinach photographs analysed with the LiMu program and represents the smallest area that can be detected and quantified using this method (bottom threshold). One pixel is larger in area than a stomata (small white rectangle), but smaller than one epidermal cell (large white rectangle). This means that a single cell lesion can be detected and quantified. We divided all detected lesions into three classes based on their area (Fig. 11A, B), namely *micro*lesions (smallest detectable area; 1 px), *macro*lesions (major plant tissue damage; > 200 px) and *meso*lesions (1–200 px lesion range).

Some image segmentation issues arose due to uneven distribution of light across the surface of the light table, so use of a global foreground/background threshold to find a leaf in the image was not satisfactory. For this reason, it was necessary to perform white balancing on the background, by estimating the picture without leaf and dividing the real picture by the estimate. This resulted in an image with a normalised background. This white balancing step in LiMu is adjusted and computed for each image individually. A good light table with uniform distribution of light is also necessary. Leaf transparency is somewhat dependent on leaf thickness and this can result in a minor segmentation issue for very thin leaves, where leaf can be mistaken for background, and for very thick leaves, where darkness represents leaf tissue, leading to recognition of dark areas as lesions. In imaging on a light table, there is a thin layer of water between light table and plant material. If excess water is present on the table during imaging, it can cause a minor leaf segmentation issue where darkness (shadow) appears at the edges of water spots, making it difficult for the program to find the leaf edge. This issue was partly solved with background white balancing. After placing a leaf on the light table, all excess water surrounding the leaf should be removed before taking an image, or a 1-mm water layer should be applied over the whole light table.

Common types of mis-segmentation (Fig. 7) were: (a) failure to recognise and segment leaf area, (b) failure to detect lesions (false negative lesions), (c) recognition of undamaged plant tissue as lesion (false positive lesions), (d) recognition of two (or more) neighbouring lesions as one larger lesion (under-segmenting) and (e) recognition of one lesion as two or more lesions (over-segmenting). Leaf area mis-segmentation occurred in < 1% of cases, where very thin and bright leaf images were mistaken for background (Fig. 7a). Lesion area mis-segmentation can occur when TB-stained areas are very dim, and hence might not be quantified (false negative) (Fig. 7b). This is more of a clustering problem, as lesions are detected initially but, since they appear more ‘dark’ than blue, they are removed as false positives in post-filtering on segments. Mis-segmentation of lesions in most cases occurred due to uneven leaf tissue clearing (false positive) (Fig. 7c). This was a major image analysis issue, due to variation in leaf tissue colour between images, and caused difficulties in finding a suitable segmentation method that would work for a variety of spinach images, irrespective of variation between different images. As a combination of blueness and darkness parameters is used to detect lesions, this issue is specific for leaves that have some darker areas (green, brown and grey) as a result of uneven clearing. To reduce this variation, optimisation of the clearing protocol for specific plant species and modification of blueness and greyness parameters is necessary. Interestingly, false positive lesions also occurred when the number of lesions on a leaf was very low. This was clearly demonstrated by the negative control images, where lesions were detected on non-TB stained leaves, i.e. false positive lesions. As leaves that are not stained with TB (negative control images) have no blue colour, the detection approach based on a combination of blueness and darkness leads to any dark area, including vascular bundles, being recognised as lesions. One solution would be to use only blueness as the definition for lesions (colour separation method). A trade-off in this case is that very dark blue-stained lesions (almost black) would be recognised as holes in the leaf, and therefore their area would not be measured accurately. Under-segmentation (Fig. 7d), i.e. recognition of two or more lesions as one, is a minor lesion segmentation issue. It occurs when lesions are so close to each other that they almost merge, with possible diffusion of the stain to neighbouring cells. In this case, it is difficult for the program to define the edge of each individual lesion and thus they are recognised as one large lesion. Over-segmentation (Fig. 7e), i.e. recognition of one lesion as two or more lesions, is also an issue of defining the lesion edge and of stain diffusing into neighbouring cells. Lesions are not stained evenly across their area, with the central part of individual lesions being darker (almost black) than the outer edges, and the program recognises this difference in intensity of blue staining. For this reason, the whole lesion area is segmented into two or more lesions, which results in a greater number of lesions being detected. A compromise must be reached when increasing the sensitivity of the method in order to quantify small, weak-stained lesions. This is a potential issue when determining lesion numbers and lesion classes, but is not an issue when assessing total lesion area or damage per leaf, as lesion number is not an indication of total lesion area on leaf scale. Thus, total lesion area per leaf is not affected by over-segmentation. A possible solution to this issue could be shrinking the lesion threshold. Additionally, segmentation steps of the proposed algorithm could potentially be replaced by a semantic segmentation model [43].

As a control for both staining and image analysis, a set of images with six different levels of standardised, artificially inflicted damage with a known pattern was prepared (Fig. 8). The artificial damage comprised low (1, 2) and high (3–5) severity levels. Comparison of results before (Fig. 8a) and after processing in the LiMu program (Fig. 8b), and after K-means based filtering and removal of false positive segments (Fig. 8c), indicated that most false positive lesions (Fig. 8b; outlined in red) were removed with post-filtering (Fig. 8c). The stained (Fig. 8a) and quantified (Fig. 9B, D) lesion area increased with increasing level of artificial damage. There was no statistically significant difference in leaf area between leaves used to represent low and high damage levels (Fig. 9A). As expected, the number of lesions detected differed between the low and high damage treatments except for damage level 5, which had a low lesion count due to introduction of cuts, recognised as large (macro) lesions. Although number of lesions varied between the treatments (Fig. 9B), due to variation in size of individual lesions the total lesion area was not predicted. Quantified lesion area (Fig. 9C) and percentage of damage per leaf area (Fig. 9D) increased with increasing introduced damage. Damage was also detected on negative control images (treatment 0) (Fig. 9B–D), but was significantly lower than in the high damage treatments. Significant differences in total lesion area (Fig. 9C) and percentage of damage per leaf (Fig. 9D) were found between low and high damage levels. A significant linear regression between number of lesions detected and lesion size (pixels per lesion) was found, indicating a separation between damage levels (Fig. 9E).

### Evaluation and validation of LiMu results

**Fig. 10:**
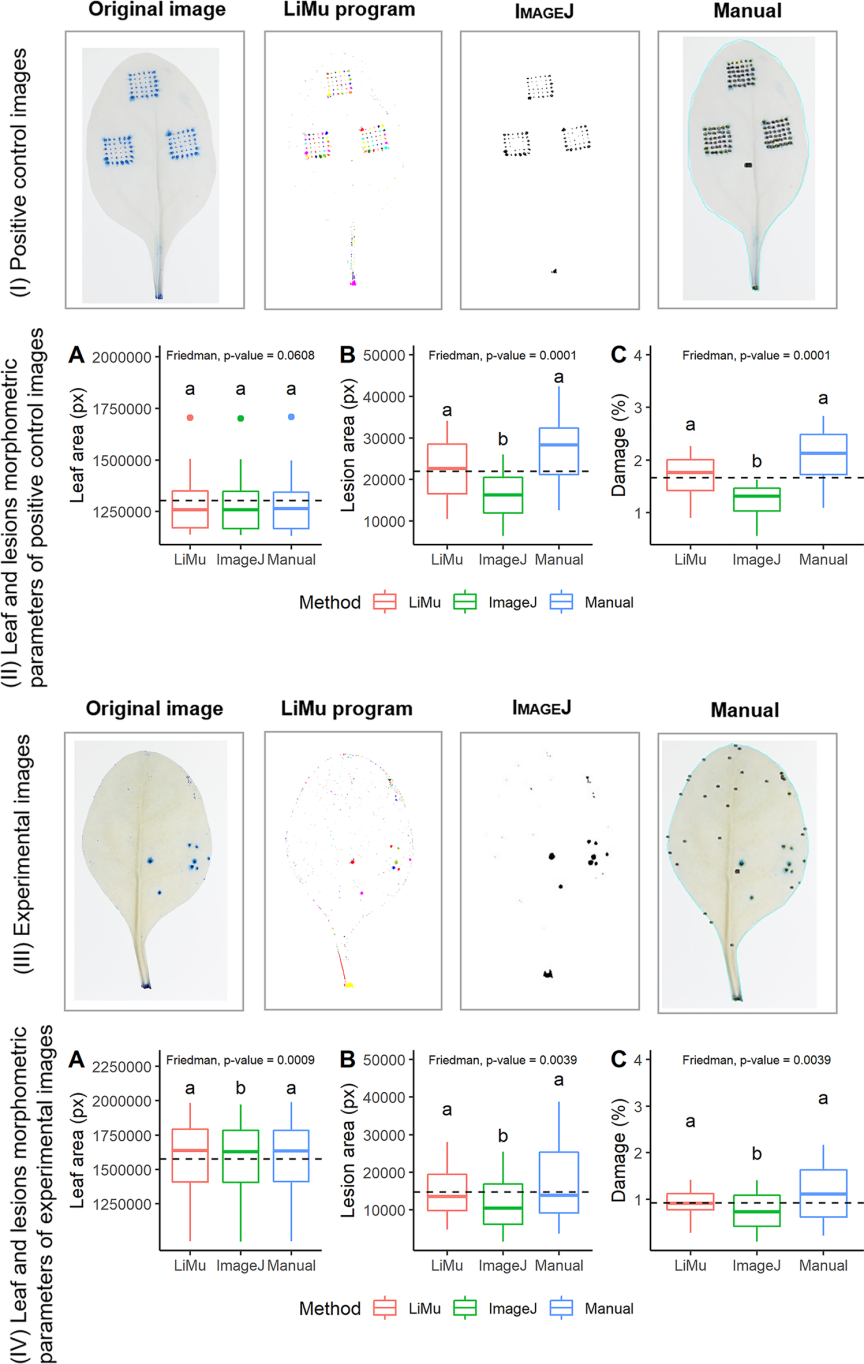
Comparison of results obtained in LiMu program, IMAGEJ and manual assessment of morphometric leaf parameters. Images used for comparisons were (I) positive control images (N = 10) and (III) randomly chosen images from the experimental dataset (N = 10). Morphometric parameters such as **A** leaf area and **B** lesion area, measured as pixels (px), and **C** leaf damage were compared (II, IV). Leaf damage was calculated as $Damage = \left( {\frac{lesion area}{leaf area}} \right) \times 100$. Dashed horizontal line represents the overall mean across damage levels. A nonparametric Friedman′s test was used, followed by Dunn′s post hoc test. Significant differences (p ≤ 0.05) identified in the post hoc test are indicated by different lower-case letters (a, b) (Additional file 7)

**Fig. 11:**
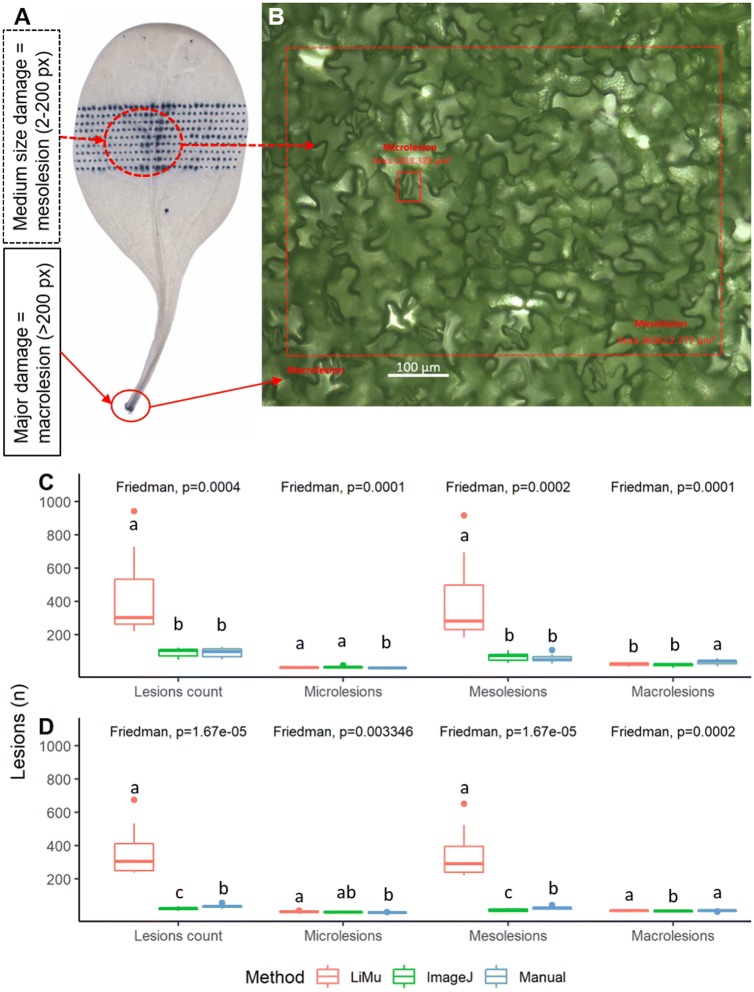
Lesion quantification and classification. **A** Image of artificially damaged and stained spinach leaf containing damage of different sizes. All detected lesions were divided into three classes, based on their area (measured as pixels (px)). **B** Definition and *in planta* demonstration of lesion classes: *microlesion* (smallest detectable area; 1 px or 1800 µm^2^) shown as a small yellow square, *mesolesion* (2–200 px; up to 360,000 µm^2^) shown as area within large dashed rectangle, and *macrolesion* (major damage, > 200 px or > 360 000 µm^2^) shown as the area outside the dashed rectangle. Images used for comparisons between methods, comprising **C** positive control images (N = 10) and **D** randomly chosen images from the experimental dataset (N = 10). Lesion classification by the LiMu program, the commonly used image analysis software IMAGEJ and manual assessment was compared. Significant differences (p ≤ 0.05) between methods (post hoc test) are indicated with different lower-case letters (a, b, c) (Additional file 7)

*Evaluation*: We performed visual and numerical comparison of the LiMu program results with results obtained using the IMAGEJ software and by manual assessment (Fig. 10). Comparisons of methods were based on leaf and lesion morphometric parameters for positive control images (Fig. 10I, II), and randomly chosen experimental images (Fig. 10III, IV). We compared leaf area (Fig. 10IIA, IVA), total lesion area (Fig. 10IIB, IVB) and damage per leaf (Fig. 10IIC, IVC) for the three methods. Results for both positive control images and experimental images followed the same trend in detection and quantification of parameters of interest. There was a significant difference between the three methods in detection of lesion area (Fig. 10IIB, IVB) and percentage leaf damage (Fig. 10IIC, IVC), for both positive control images and experimental images. Total lesion area and percentage leaf damage were significantly higher for LiMu detection than for IMAGEJ, but not manual assessment. As the freehand tool used for manual segmentation by rounding of individual lesion areas is not completely precise, it might lead to slight area overestimation. In terms of time requirement, LiMu was significantly less time-consuming than manual assessment. The selection capabilities of IMAGEJ did not provide the level of detail afforded by LiMu. In addition, the adaptability and consistency of LiMu was very good for both completely and unevenly cleared leaves and this method was more likely to correctly segment objects than the form of ‘fixed’ threshold used in IMAGEJ. In addition, the LiMu program allows correction by filtering out false positive lesions through K-means based clustering on the extracted data, which is not the case with manual assessment or IMAGEJ. Comparisons of the three methods in terms of total number of detected lesions and lesion classes revealed that a significantly larger number of lesions was quantified with the LiMu program than with the other two methods, both for positive control images (Fig. 11C) and experimental images (Fig. 11D). A significantly higher number of microlesions was detected with the LiMu program than with manual assessment, but not IMAGEJ. This is because in most cases microlesions are not visible to the naked eye and therefore cannot be assessed manually. A significantly higher number of mesolesions was detected with the LiMu program than with IMAGEJ and manual assessment, whereas a significantly higher number of macrolesions was detected with manual assessment than with the LiMu program for positive control images, but not for experimental images. This might be a result of over-segmentation in manual assessment, because finding lesion edges manually is not as precise as it is with LiMu or IMAGEJ.

Manual segmentation of individual lesion areas cannot be regarded as absolute ‘truth’, as it is limited by the ability of the human eye to discern lesions (stained areas). Depending on the number of lesions, it can also be a very tedious and time-consuming process and somewhat subjective. Manual assessment is thus not suitable for large-scale image analysis and for detection of very dim and small lesions. It is possible, but rather complex, to segment all damage using the IMAGEJ default threshold. On the other hand, the LiMu program’s local thresholding method, using square disc filters, is robust and computes a threshold individually for each image. Although the results obtained using IMAGEJ and the LiMu program were very similar for the selected images, IMAGEJ was not adequate for batch processing of a large number of spinach leaf images due to large variations in the colour of cleared leaf tissue and the size and intensity of blue-stained areas. There was variation in the colour of cleared leaf tissue between different leaf samples, but also between different parts of the same leaf, and in most cases this led to the introduction of false positives (Additional file 4). Large variation prevented the same threshold being used for all images tested without generating many segmentation errors, although it functioned satisfactorily for measurement of leaf area. Therefore, to apply IMAGEJ batch processing, it would be necessary to pre-classify images into groups to minimise the variation between images in the same group, and adapt a threshold for the groups individually. This would be time consuming and would involve a certain level of subjectivity.

**Fig. 12:**
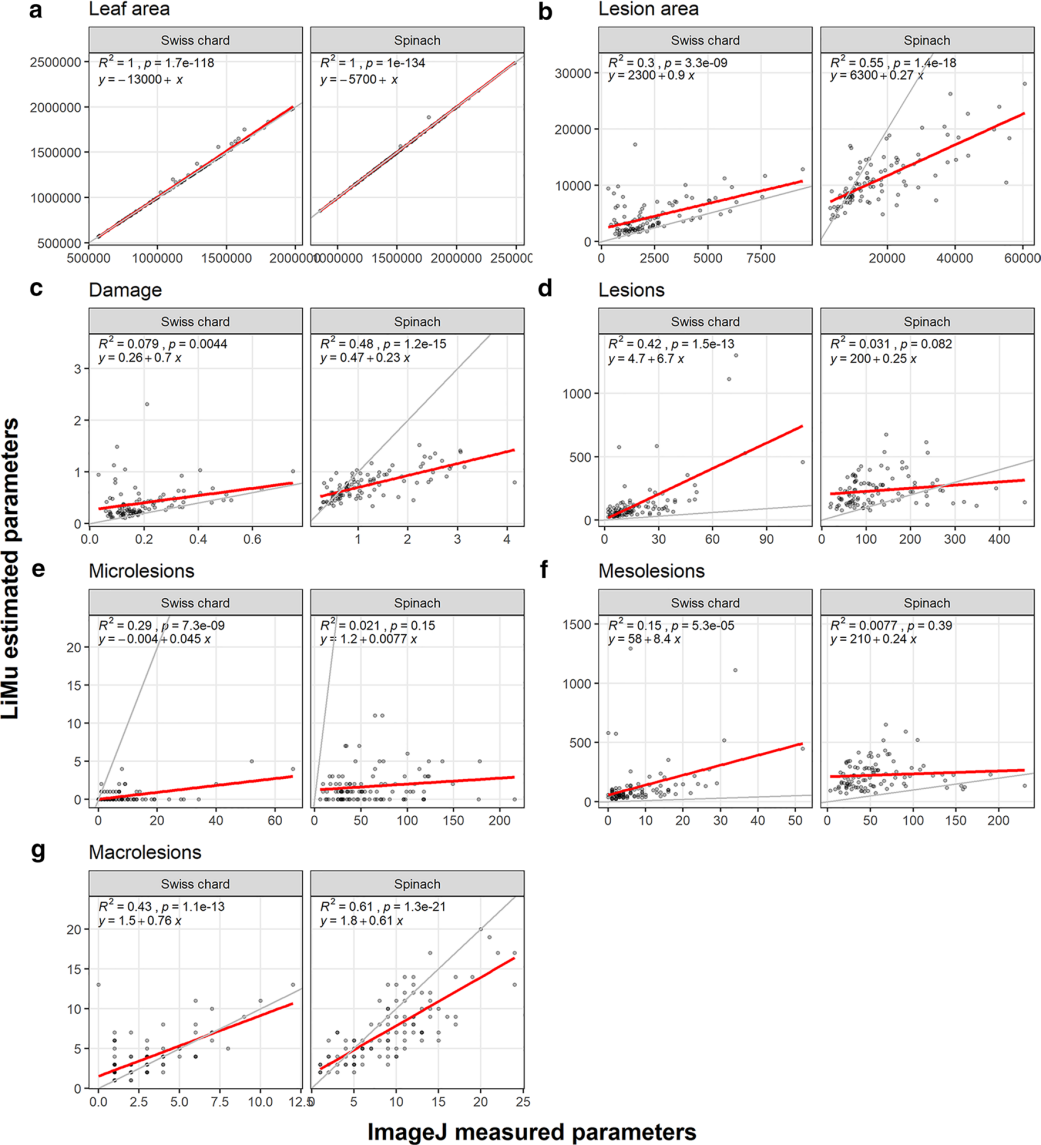
Comparison of IMAGEJ-measured and LiMu-estimated leaf morphometric variables for 50% of the experimental dataset. The variables **a** leaf area (pixels), **b** lesion area (pixels), **c** leaf damage (%), and number of **d** lesions, **e** microlesions (1 pixel), **f** mesolesions (2–200 pixels) and **g** macrolesions (> 200 pixels) were assessed. Regression line (red) showing the result of the model: $y_{LiMu} = \beta_{0} + \beta_{1} x_{ImageJ}$ and line with slope 1 and intercept 0 (grey) are shown

*Validation:* The results of simple linear regression analysis for both tools are presented in Table 1 and Fig. 12.

**Table 1:** Results of linear regression analysis on leaf morphometric variables for Swiss chard and spinach

	Swiss chard			Spinach		
	R^2^ (adjusted)	Intercept	Slope	R^2^ (adjusted)	Intercept	Slope
Leaf area (px)	5.6e−02	0.13	9.7e−03**	2.5e−03**	0.41	0.39
Lesion area (px)	4e−03**	1.8e−07***	0.46	0.89	2e−16***	2e−16***
Damage (%)	6e−03**	9.3e−06***	0.21	0.91	2e−16***	2e−16***
Lesions (n)	0.34	0.82	8e−11***	0.21	3.7e−15***	8.5e−07***
Microlesions (1 px)	0.99	0.97	2e−16***	0.99	2.5e−03**	2e-16***
Mesolesions (2–200 px)	0.11	0.02*	3.3e−04***	0.06	2e−16***	7e−03**
Macrolesions (> 200 px)	0.05	4.9e−06***	8e−03**	0.38	9.5−04***	3.5e−12***

***p < 0.001, **p < 0.01. *p < 0.05. LiMu results are used as the explained variable and IMAGEJ results as the explanatory variable. Linear regression analysis was performed on 50% of the experimental dataset. The results shown are for the model: $y_{LiMu} - x_{ImageJ} = \beta_{0} + \beta_{1} x_{ImageJ}$

Leaf area: The results obtained using IMAGEJ validated LiMu results for the variable leaf area, i.e. the estimated regression line indicated a perfect fit between the observed IMAGEJ and predicted LiMu models. Lesion area, damage (%) and number of lesions: For these three variables in Swiss chard, the estimated regression line was above the perfect fit line. This indicates that the LiMu model predicted significantly higher lesion area, percentage leaf damage and number of lesions than observed with IMAGEJ. However, for lesion area and damage, the slope when comparing LiMu predictions and IMAGEJ observations did not differ, indicating that the over-estimation by LiMu was the same regardless of size of lesion area or percentage leaf damage for Swiss chard. For number of lesions the slope differed, i.e. the number of lesions predicted by the LiMu model compared with observed IMAGEJ outcomes varied with the number of lesions. For spinach, the slope was significantly different between LiMu and IMAGEJ models for these three variables. Outcomes predicted by the LiMu model varied with the value of the input variable compared with the IMAGEJ model.

Microlesions, mesolesions, macrolesions (n): The observed regression line for microlesions was below the perfect fit line and there was a significant slope for both spinach and Swiss chard. This indicates that the LiMu model predicted a significantly lower number of microlesions than was observed with IMAGEJ, irrespective of the value of the input variable. The observed regression line for mesolesions was above the perfect fit line for both leafy vegetable species. This indicates that the LiMu model predicted a significantly higher number of mesolesions than was observed with IMAGEJ, irrespective of the value of the input variable. Outcomes predicted by the LiMu model for macrolesions varied with the value of the input variable compared with the IMAGEJ model for both leafy vegetable species.

The major difference between the two tools was for the parameters lesion area and percentage leaf damage in spinach and number of microlesions and mesolesions in both species. It can be speculated that the difference between the two tools with respect to lesion quantification is partly due to the fact that the IMAGEJ workflow does not include post-filtering as a form of error correction step (Additional file 4), and thus false positive lesions induced by incomplete clearing are not removed. A certain number of microlesions detected with LiMu are removed in the post-filtering step, due to weak staining and small size (Fig. 2e). Hence it can be expected that false positive lesions one pixel in size are mis-classified as microlesions. It can also be speculated that the choice of threshold algorithm selected in IMAGEJ underestimated individual lesion areas, thereby classifying some mesolesions as microlesions.

### Application of the method on the experimental dataset

**Fig. 13:**
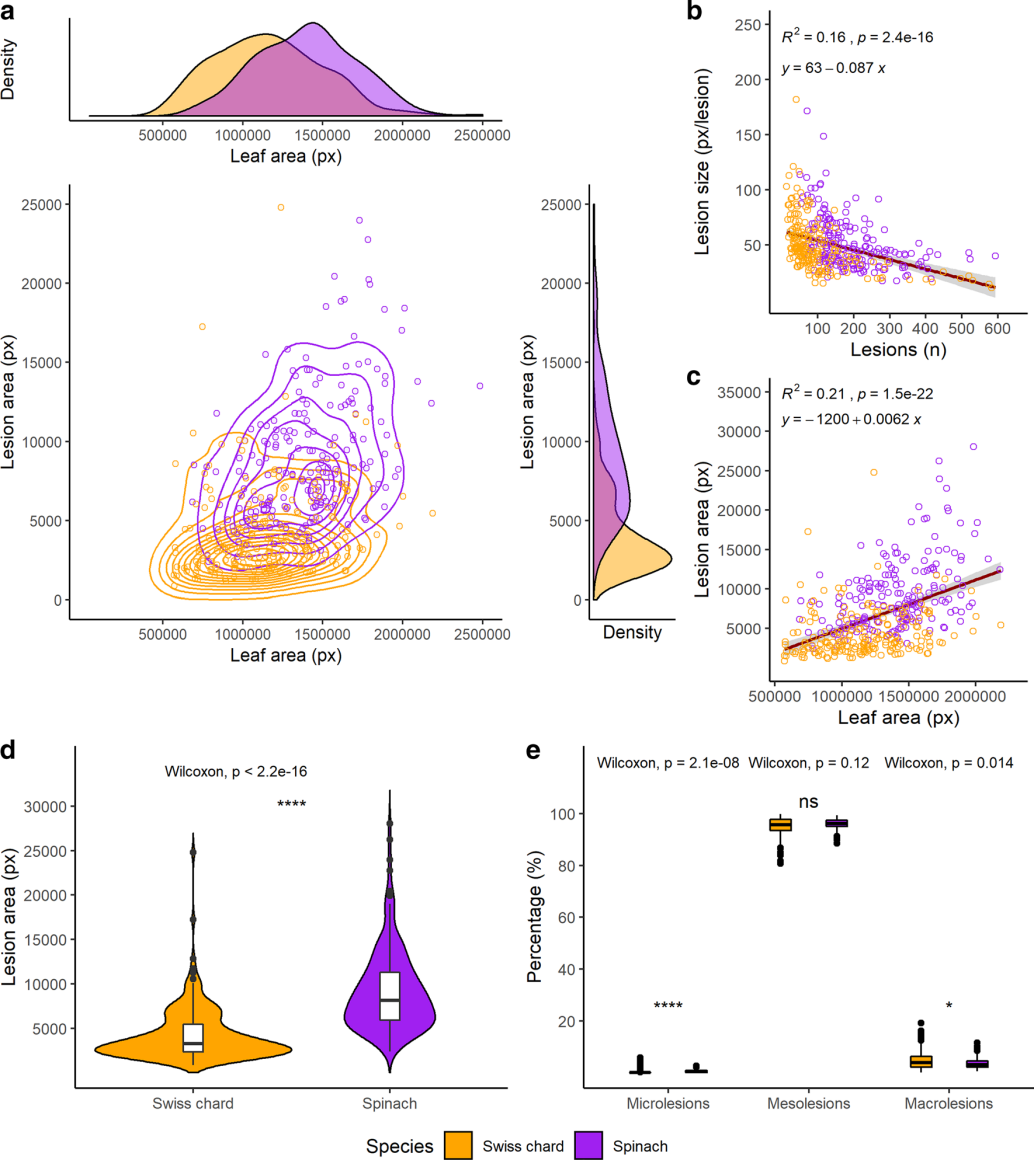
Results obtained for Swiss chard and spinach leaf and lesion parameters using the LiMu program. **a** Distribution of lesion area on spinach and Swiss chard leaves in scatter and marginal density plots. Correlations between **b** lesion number and size of individual lesions, and **c** leaf and lesion areas. Differences between Swiss chard and spinach in **d** lesion area and **e** relative distribution of different lesion classes between the two species quantified with the LiMu program. A non-parametric Wilcoxon test was used for comparisons of mean values between the two species (**d**, **e**). Significant differences (p ≤ 0.05) between the species are indicated with an asterisk (*) (Additional file 7)

Using the experimental dataset, we demonstrated that LiMu can be used to effectively quantify damage to Swiss chard leaves (Fig. 13). We then compared differences in quantified parameters between the two species. The scatter plot with marginal density plots in Fig. 13a shows the distribution of lesion area and leaf area of spinach and Swiss chard samples used in this experiment. Leaf areas occupied by larger lesions (macrolesions) were greater in spinach, while the proportion of microlesions and concomitant leaf areas occupied by microlesions were higher in Swiss chard (Fig. 13a). As expected from the experimental set-up, lesion number was positively correlated with lesion size, which explained 16% of the variations in lesion numbers (Fig. 13b). Likewise, increasing leaf area was related to increasing total lesion area (Fig. 13c), with a higher proportion of leaf area occupied by lesions in spinach, as confirmed by the Wilcoxon test (Fig. 13d). The separation into different lesion classes (micro-, meso- and macrolesions) revealed significant differences in the relative distribution of microlesions and macrolesions between spinach and Swiss chard (Fig. 13e).

The main aim in this paper was to provide a detailed description of the steps followed in development of a leaf-scale damage detection and quantification method, using parameters such as leaf and lesion areas, leaf-scale damage and lesion classes as examples. Numerous lesion parameters can be extracted using the LiMu program (Additional file 8). Parameters of biological relevance which may have the greatest utility in future studies are lesion area, shape descriptors (circularity, eccentricity, height and width, diameter, perimeter) and location (distance to leaf edge and to central line-midrib). Combining some of these parameters (e.g. area, location and circularity) would allow lesion classification (e.g. cuts, dot-like lesions), providing more information about the position and origin of the damage-lesion relationship (co-localisation) and the link to specific post-harvest processing steps.

## Conclusions

To meet the need for more automated damage detection and quantification approaches within the agriculture sector and food processing industry, we developed a high-throughput, automated and robust method for detection and quantification of lesions on leaf scale.

Leaf samples can vary widely with respect to size, thickness and maturity, and therefore optimisation of the leaf clearing step to remove chlorophyll is vital. Uneven clearing increases variation between and within leaf samples and leads to feature mis-segmentation. We found that a combination of clearing and staining provided a good colour contrast between intact and damaged leaf tissue and increased the sensitivity of the method. The great advantage of the method is that the staining with TB dye visualises all damage on leaf scale, even when visible symptoms are absent, facilitating early-stage damage detection. In tests, the approach enabled detection of large (macro) and single-cell (micro) lesions and automated quantification, classification and description of lesion parameters on leaf scale.

The method can be used for analysis of leafy vegetables post-harvest, in particular to identify critical steps introducing damage within the chain. With the method, it is possible to investigate whether the location, shape and size of individual lesions are specific to certain post-harvest steps and where on the leaf damage is more likely to occur (location). In-depth knowledge concerning lesion quantity and their morphometric parameters may be used for generating prediction models and risk assessments for economic losses and produce shelf-life. Our method enables large-scale screening for early-stage plant susceptibility to specific pathogens, and can be used in the identification of less susceptible plant cultivars. In addition, cell damage due to interactions of plant cultivar, pathogen strain and environmental factors can be assessed at different time points from inoculation to generation of first visible infection symptoms using the proposed approach. Finally, the information provided by the method regarding tissue damage on leaf scale allows correlations between leaf damage severity and infection and internalisation rate of specific opportunistic plant pathogens to be investigated.

## Supplementary information

**Additional file 1.** The LiMu image analysis program script.

**Additional file 2.** Lesion cluster classes. Lesion data were post-filtered and classified into 30 clusters, with each individual lesion in the text file assigned a number from 0 to 29 (true and false positive lesions).

**Additional file 3.** IMAGEJ macro for leaf area and damage quantification.

**Additional file 4.** Differences between IMAGEJ and LiMu image analysis workflows.

**Additional file 5.** Cost-effectiveness of the proposed method. Laboratory set-up.

**Additional file 6.** Visualisation of damaged and intact leaf tissue. Micrographs of damaged unstained leaf tissue and damaged and intact trypan blue-stained leaf tissue.

**Additional file 7.** Statistical analysis results. **Table S1.** Results of LiMu image analysis using negative and positive control images. **Table S2.** Comparison between LiMu program, IMAGEJ and manual assessment of morphometric leaf and lesion parameters. **Table S3.** Comparison in lesion class quantification between LiMu program, IMAGEJ and manual assessment. **Table S4.** LiMu program results for leaf and lesion parameters, using the experimental image dataset.

**Additional file 8.** Raw dataset. LiMu image analysis output.

## Data Availability

The image datasets used and analysed during the study are available from the corresponding author on reasonable request. The original LiMu code is made freely available in the Python Package Index (PyPI), and can be downloaded from https://pypi.org/project/limu/. It is also supplied as a text file in Additional file 1. Datasets supporting the conclusions in this article are included within the article (and its additional files).

## Abbreviations

AB: Aniline blue dye
BBCH: Scale used to identify the phenological development stages of plants (“Biologische Bundesanstalt, Bundessortenamt und CHemische Industrie”)
DBSCAN: Clustering algorithm (Density-Based Spatial Clustering of Applications with Noise)
OR: Logical operator
Px: Pixel
ROIs: Regions of Interest
RGB: Colour image (red–green–blue)
TB: Trypan blue dye
