# Supplementary material for: High-throughput method for detection and quantification of lesions on leaf scale based on trypan blue staining and digital image analysis
Source: Plant Methods. 2020 May 4;16:62. doi: 10.1186/s13007-020-00605-5 (PMC7197134; doi:10.1186/s13007-020-00605-5)

K-MEANS BASED CLUSTERS

True positive clusters: 0, 3, 4, 5, 8, 9, 10, 12, 13, 15, 18, 24, 26, 27, 28, 29

False positive clusters: 1, 2, 6, 7, 11, 14, 16, 17, 19, 20, 21, 22, 23, 25

CLUSTER 0

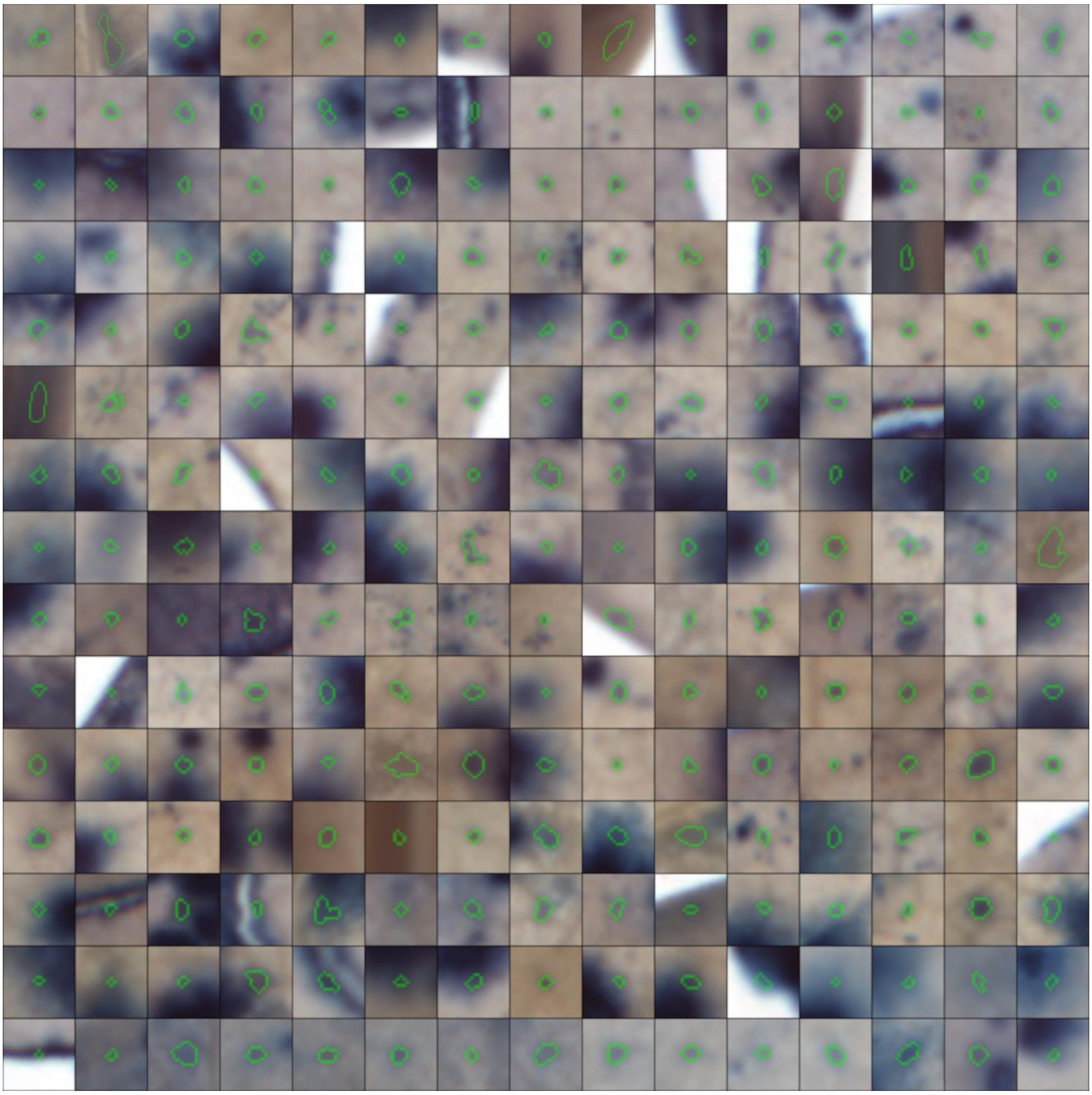

CLUSTER 1

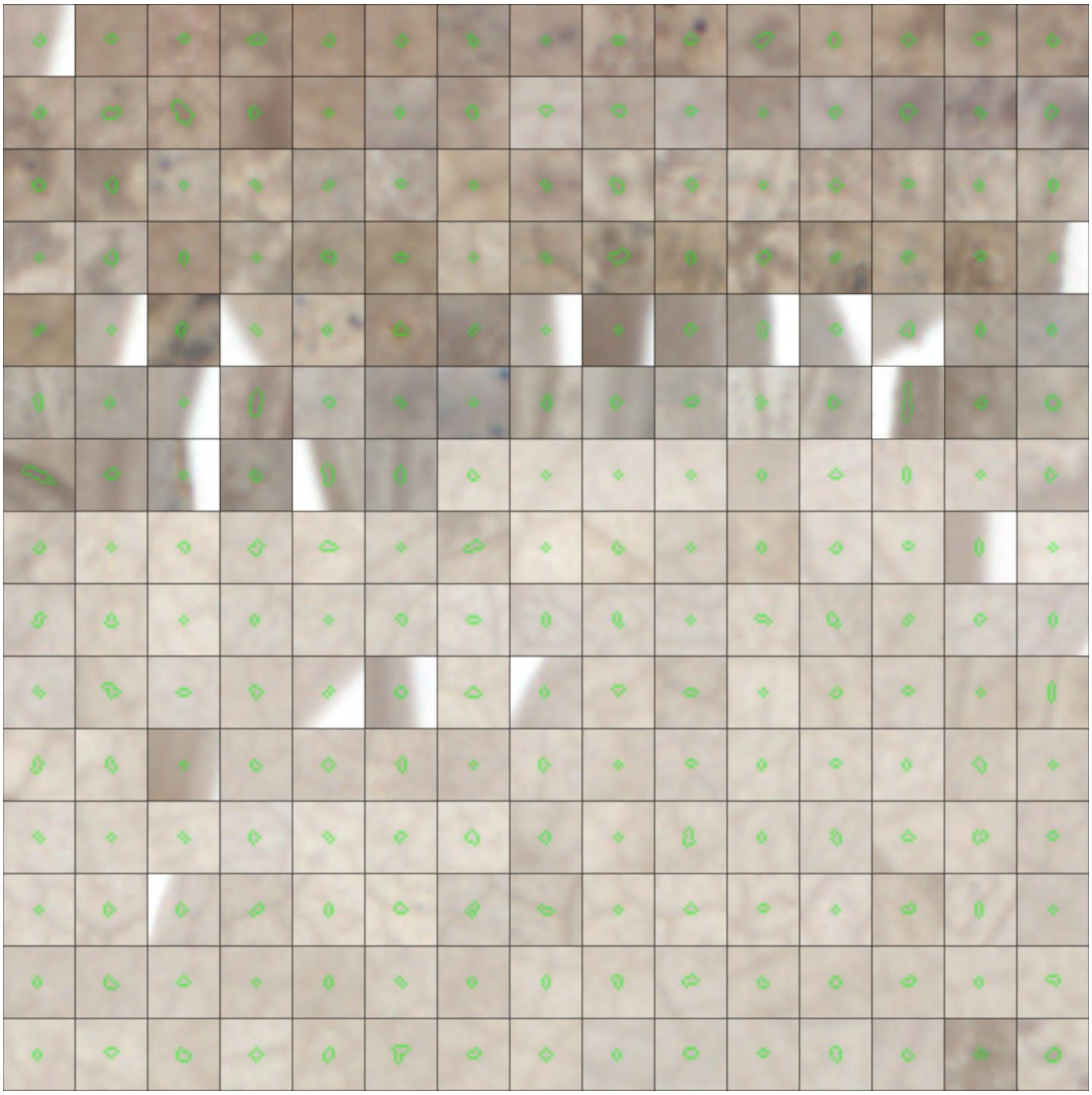

CLUSTER 2

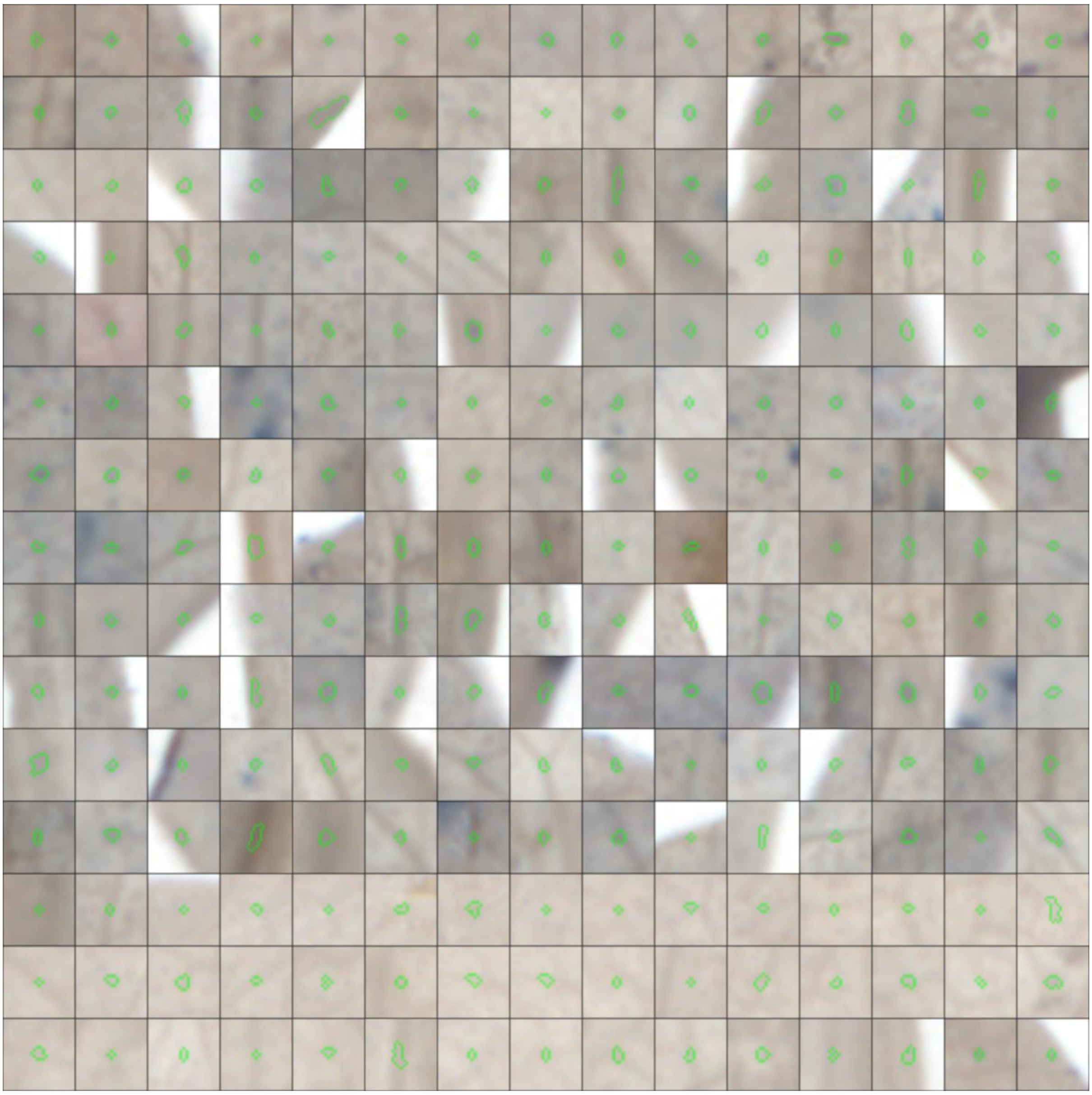

CLUSTER 3

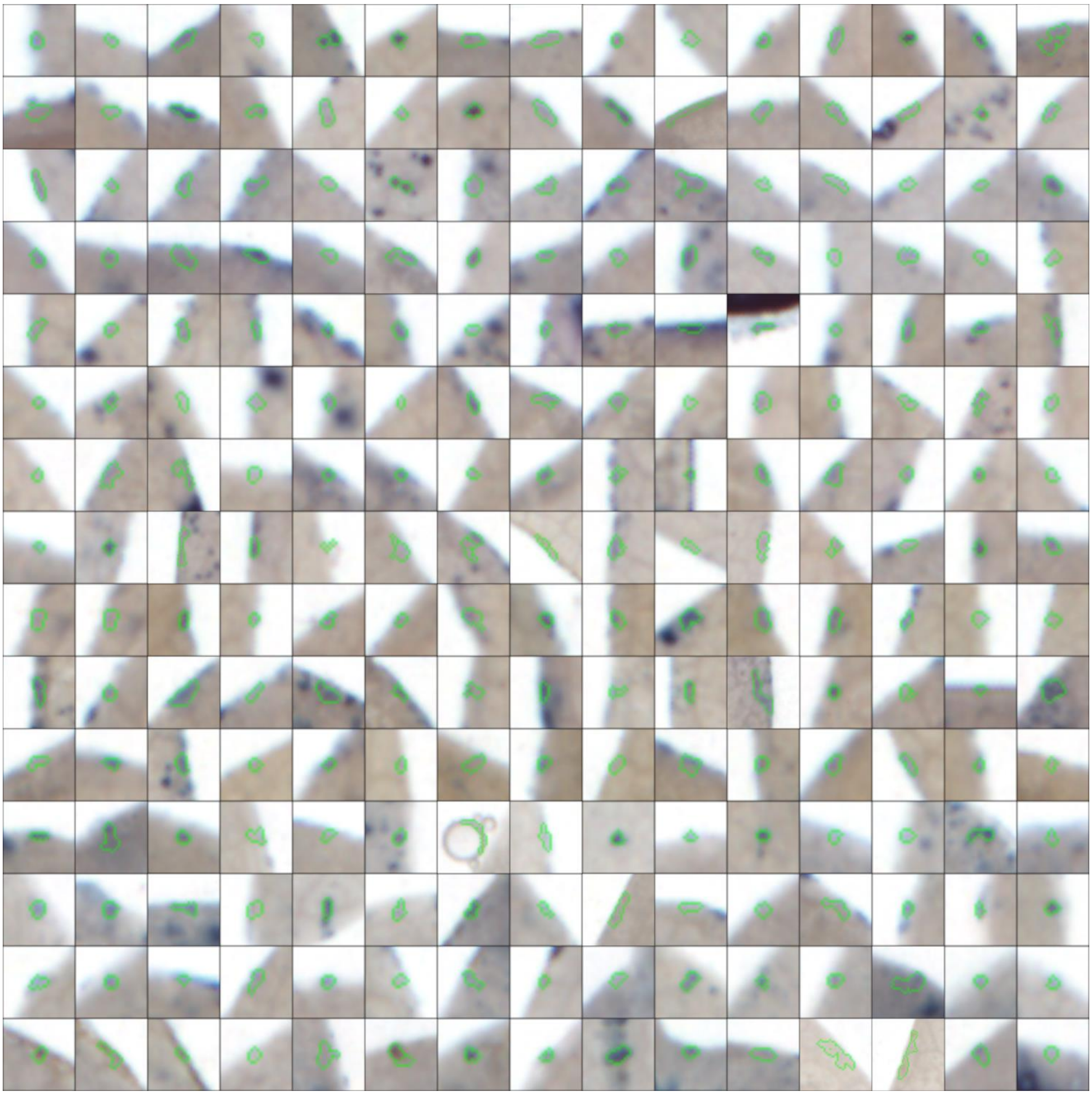

CLUSTER 4

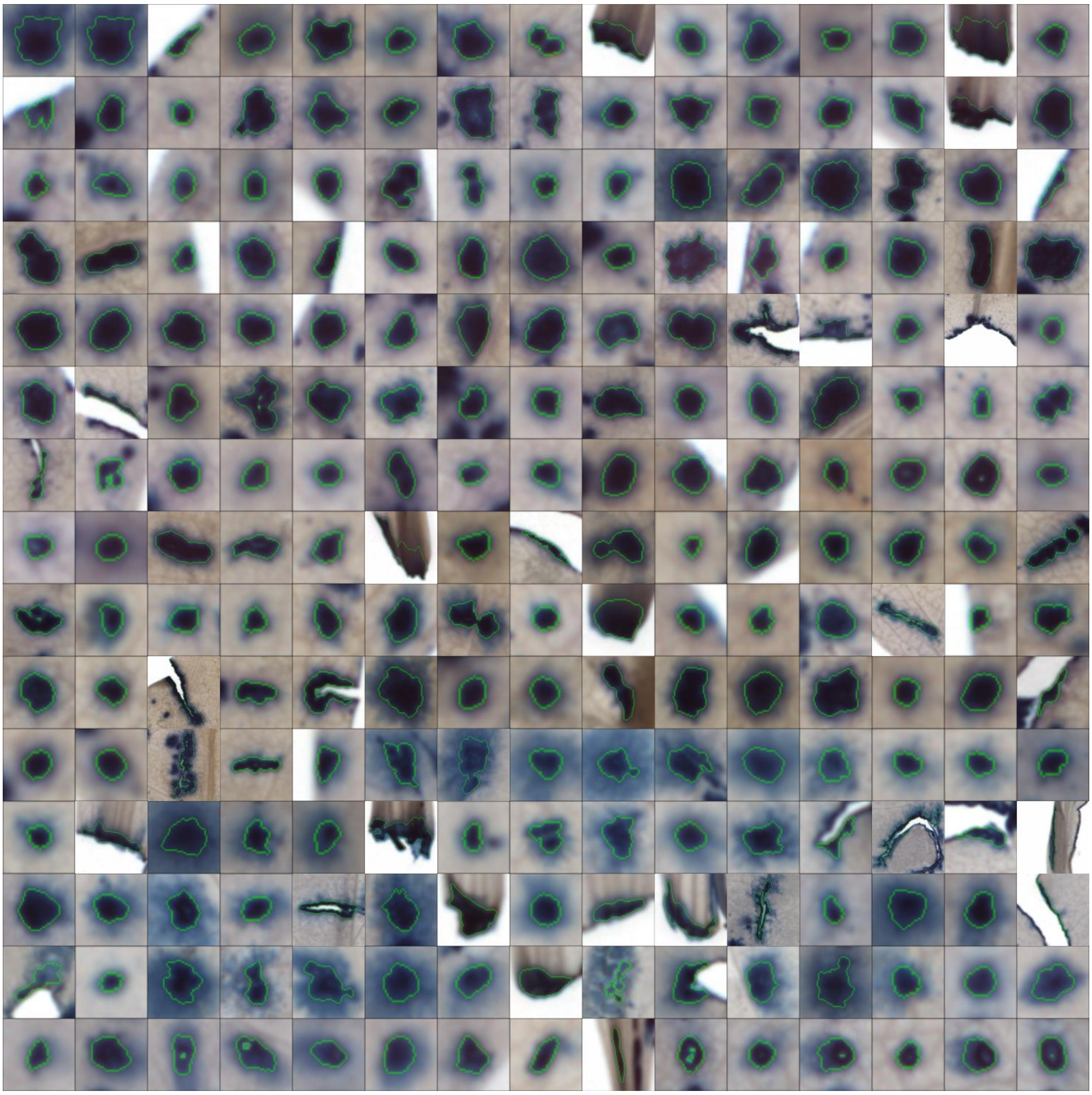

CLUSTER 5

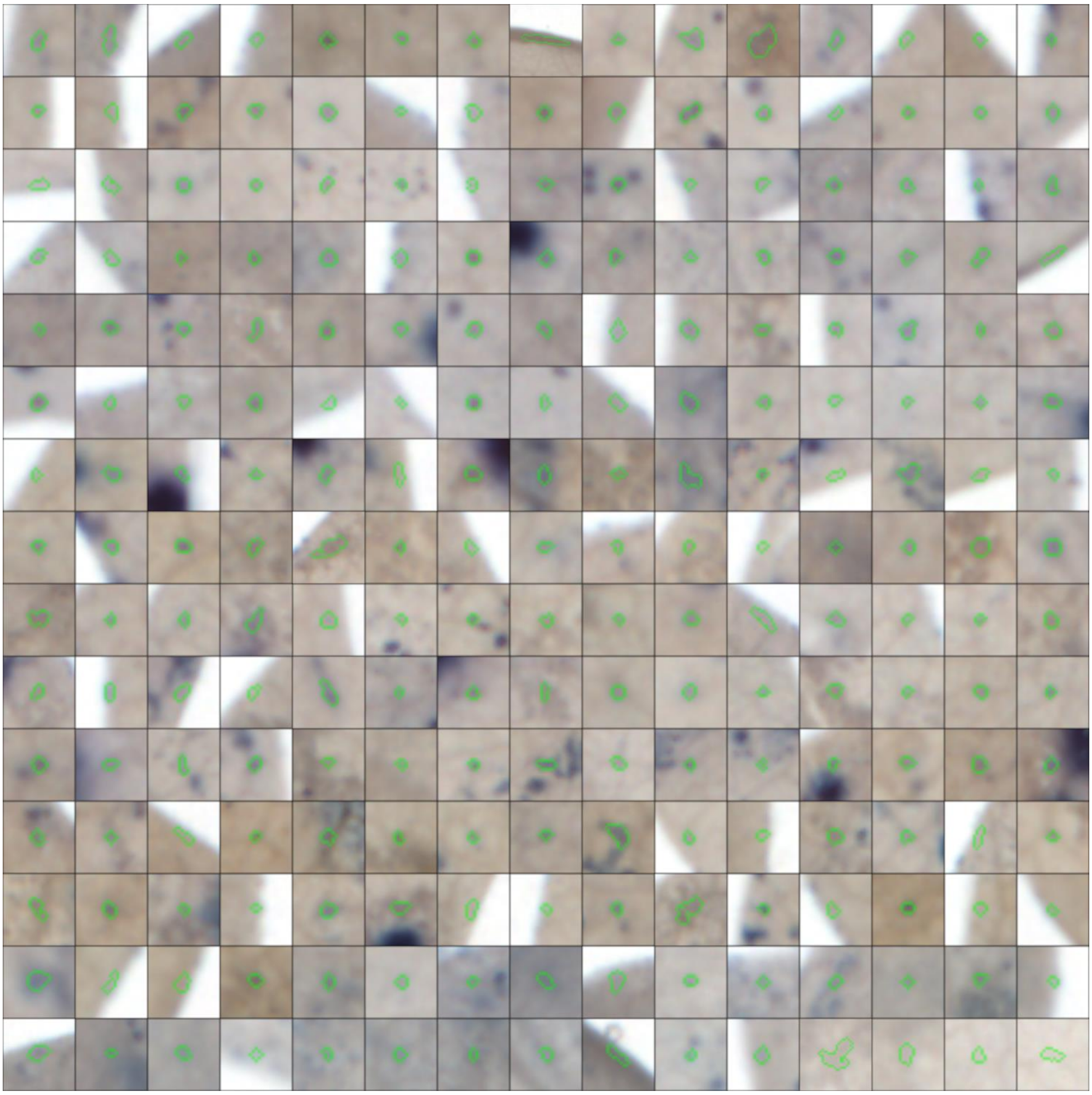

CLUSTER 6

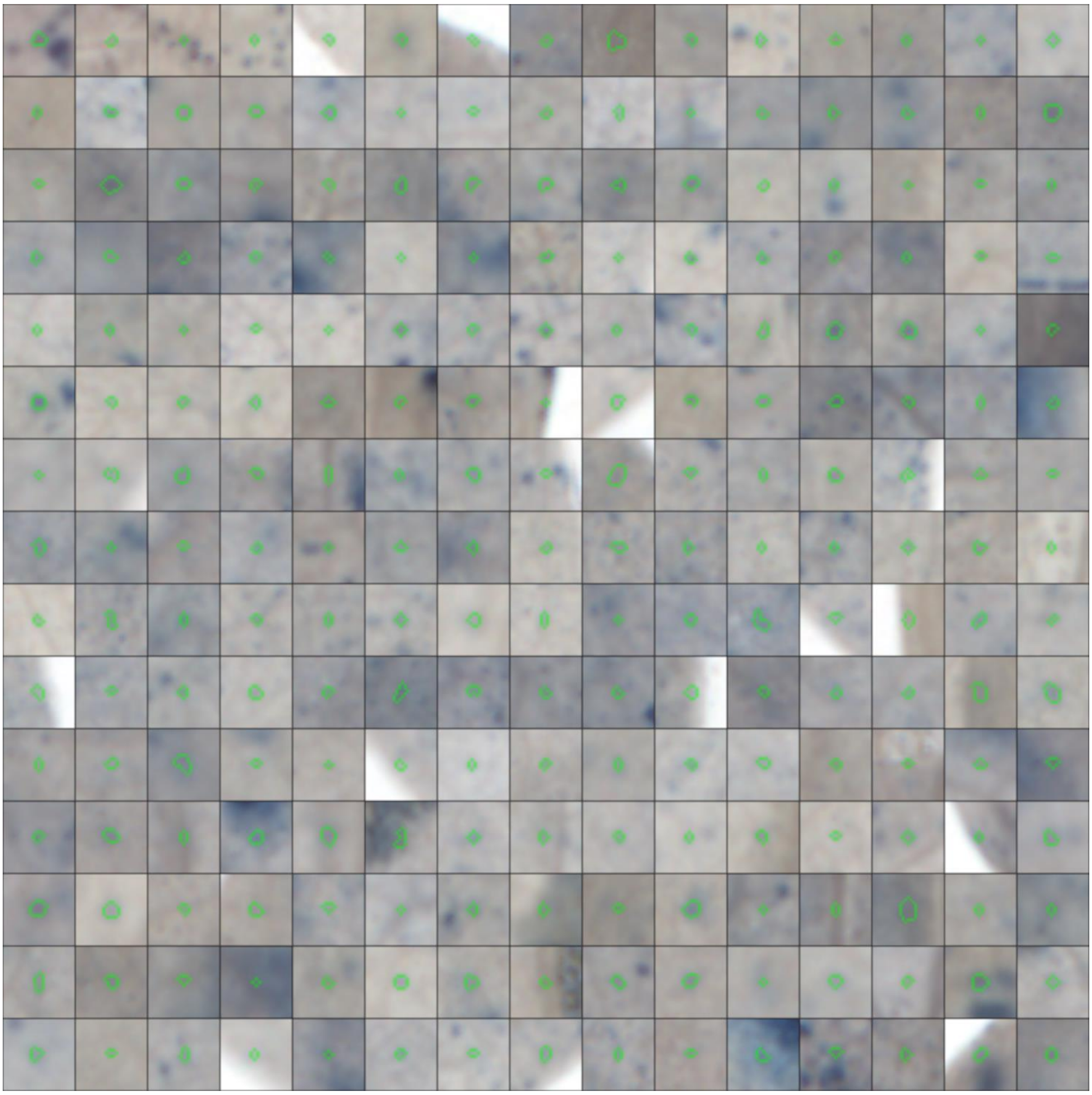

CLUSTER 7

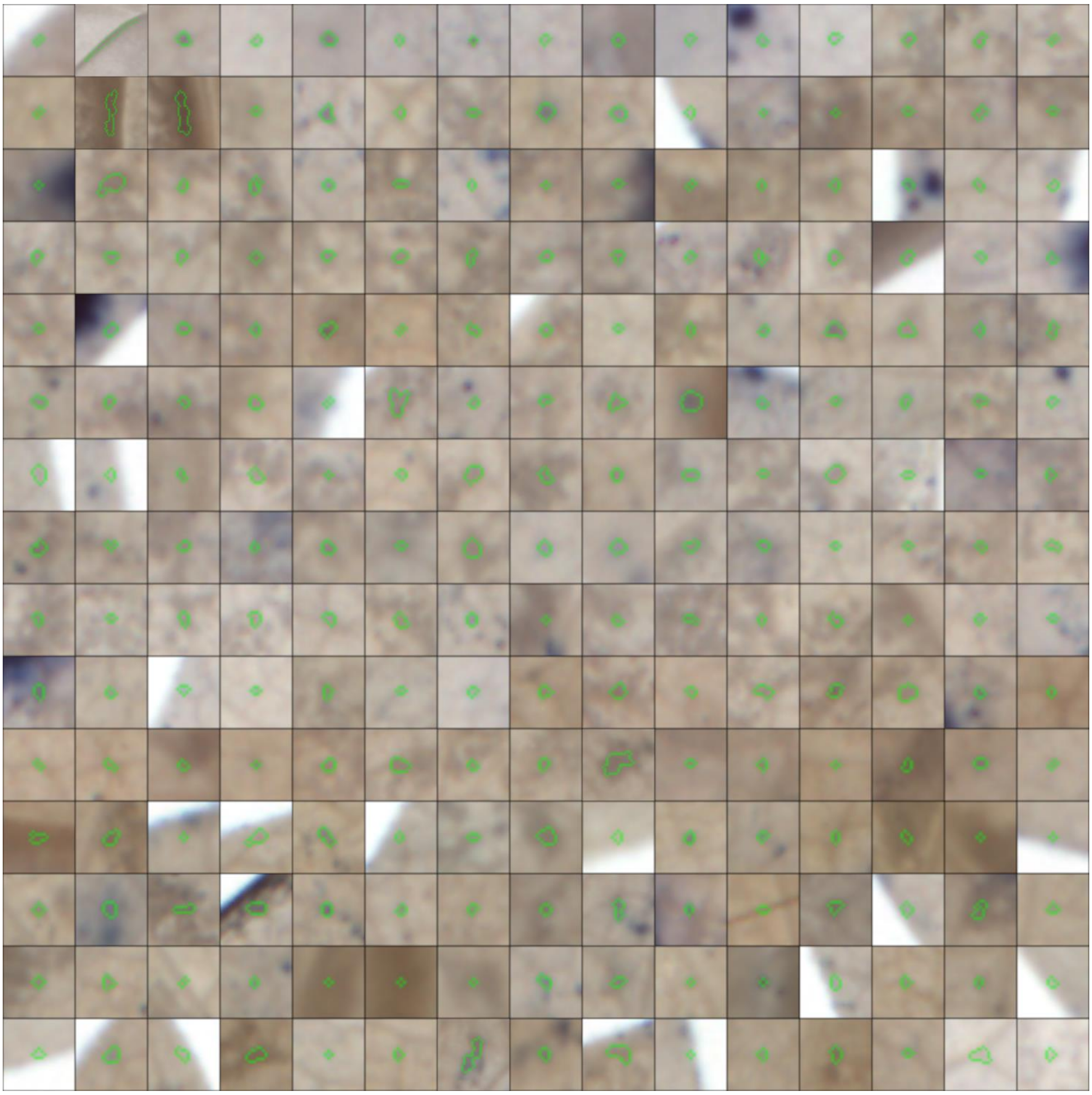

CLUSTER 8

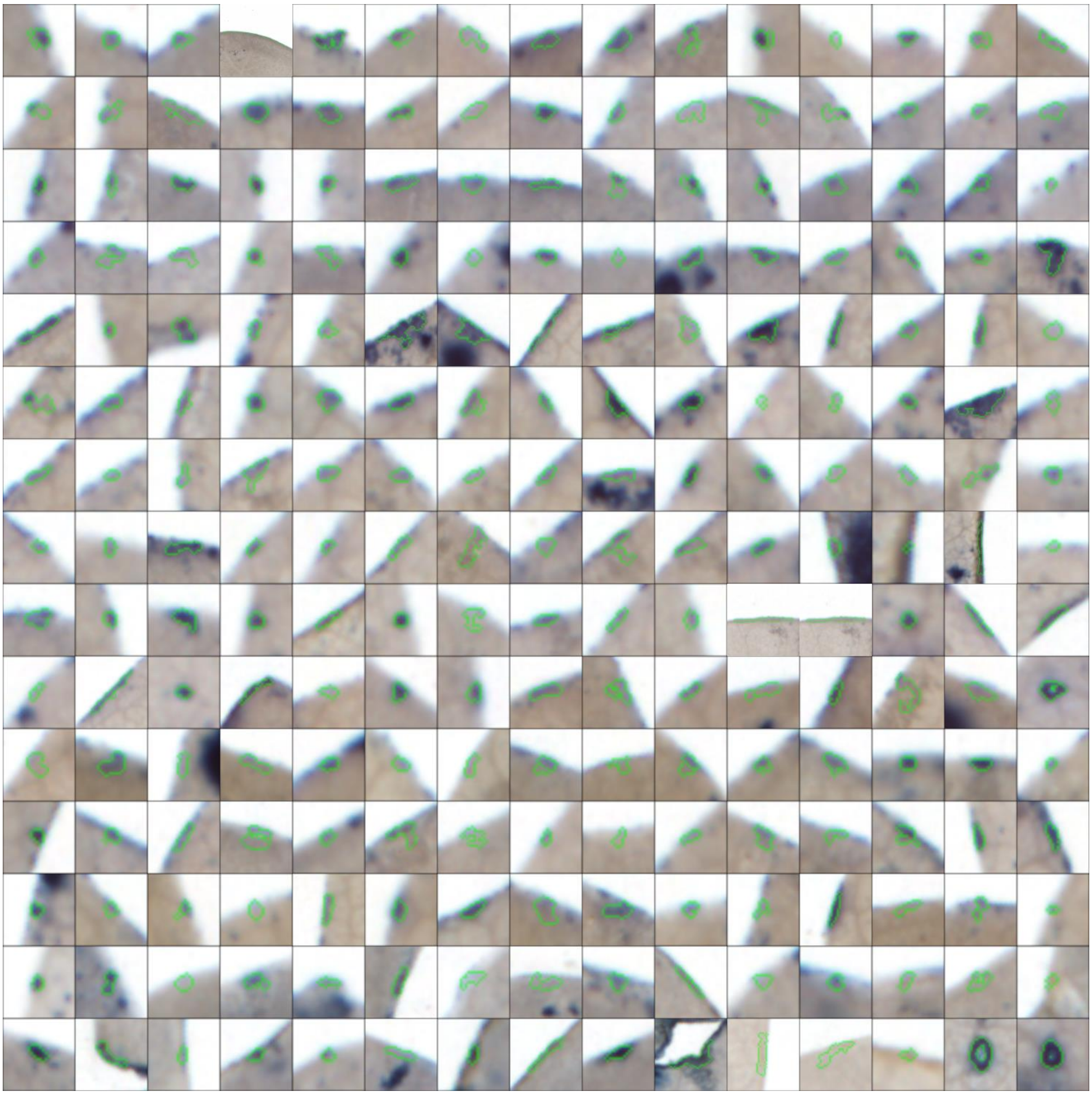

CLUSTER 9

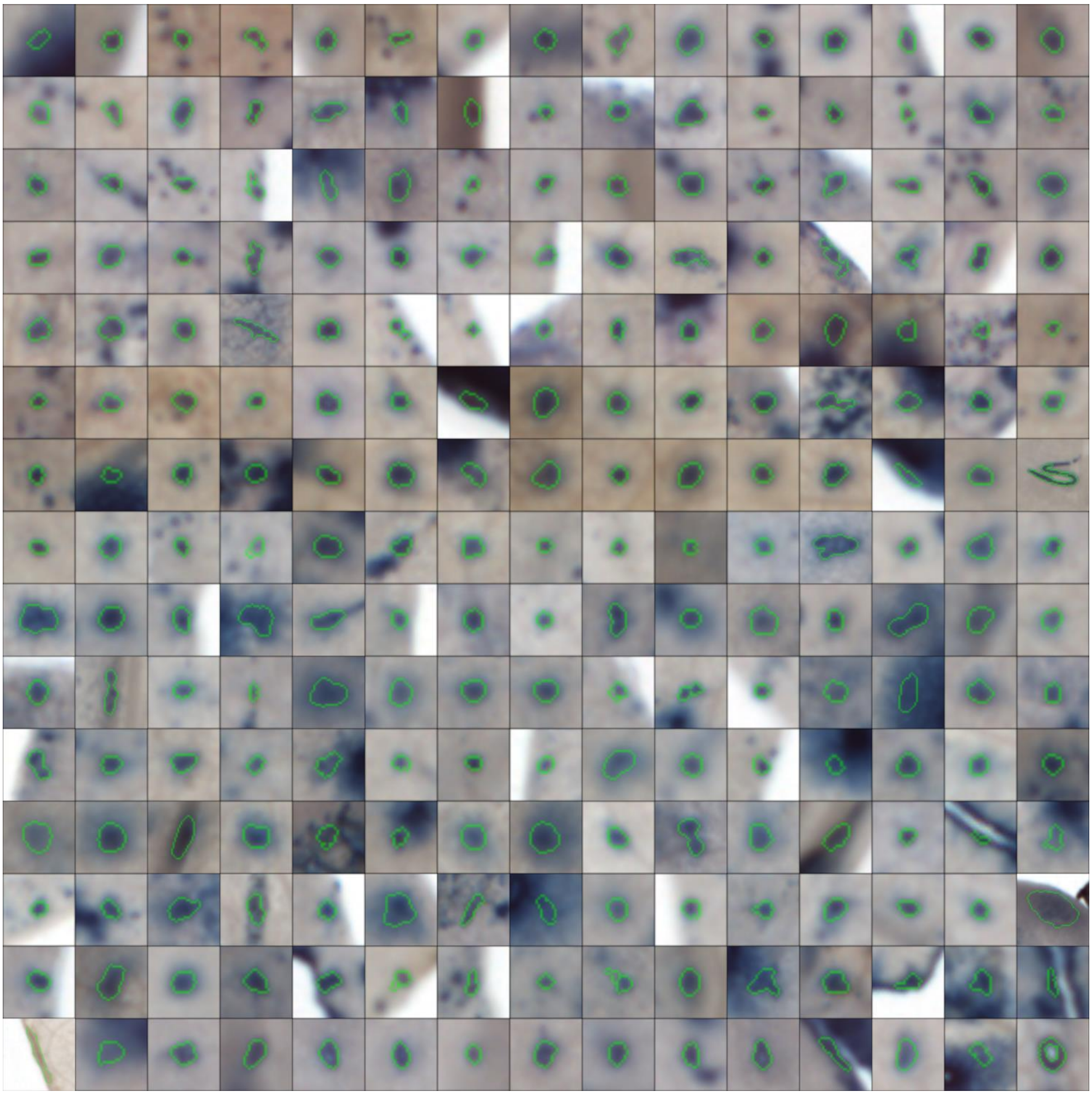

CLUSTER 10

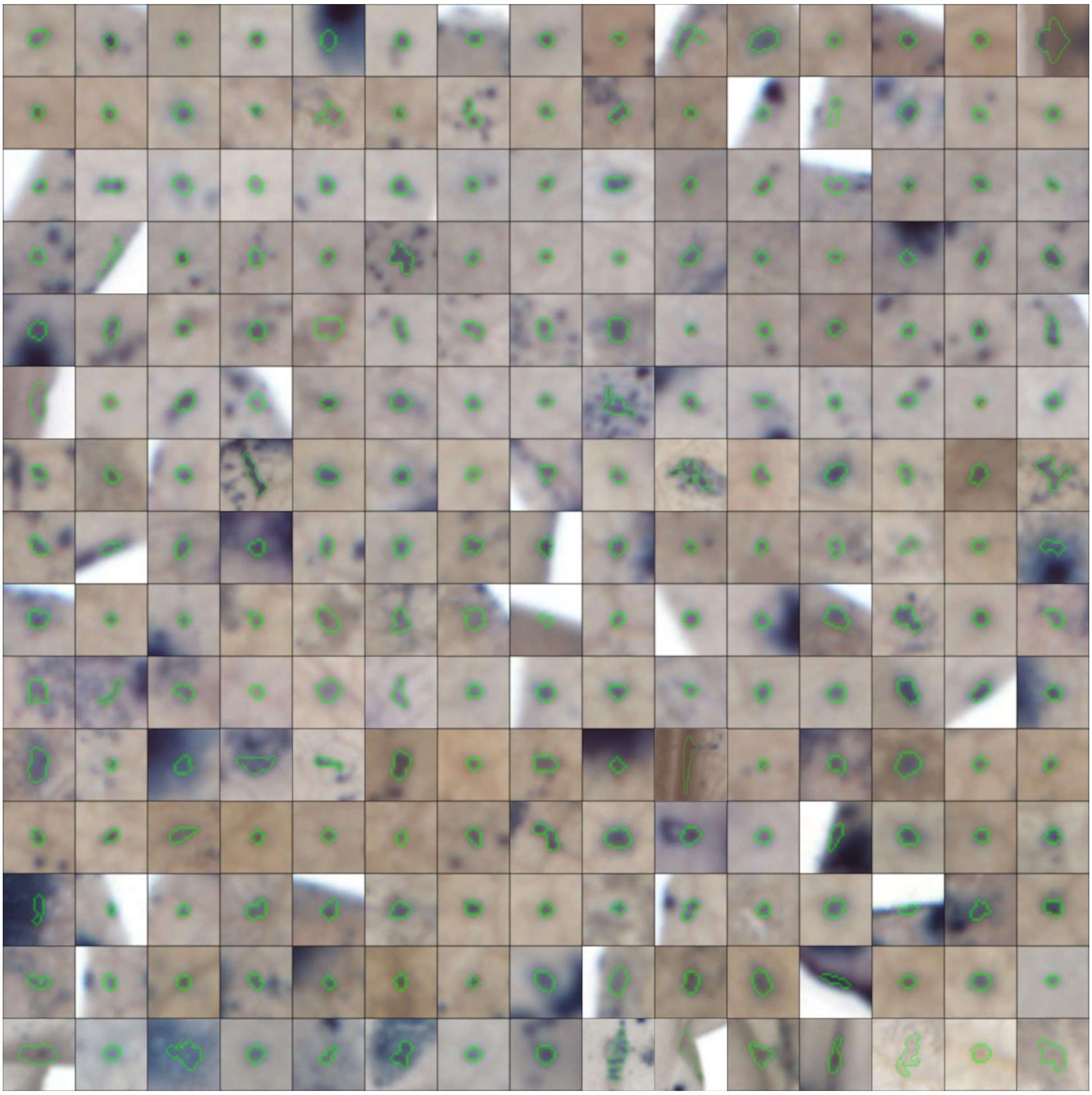

CLUSTER 11

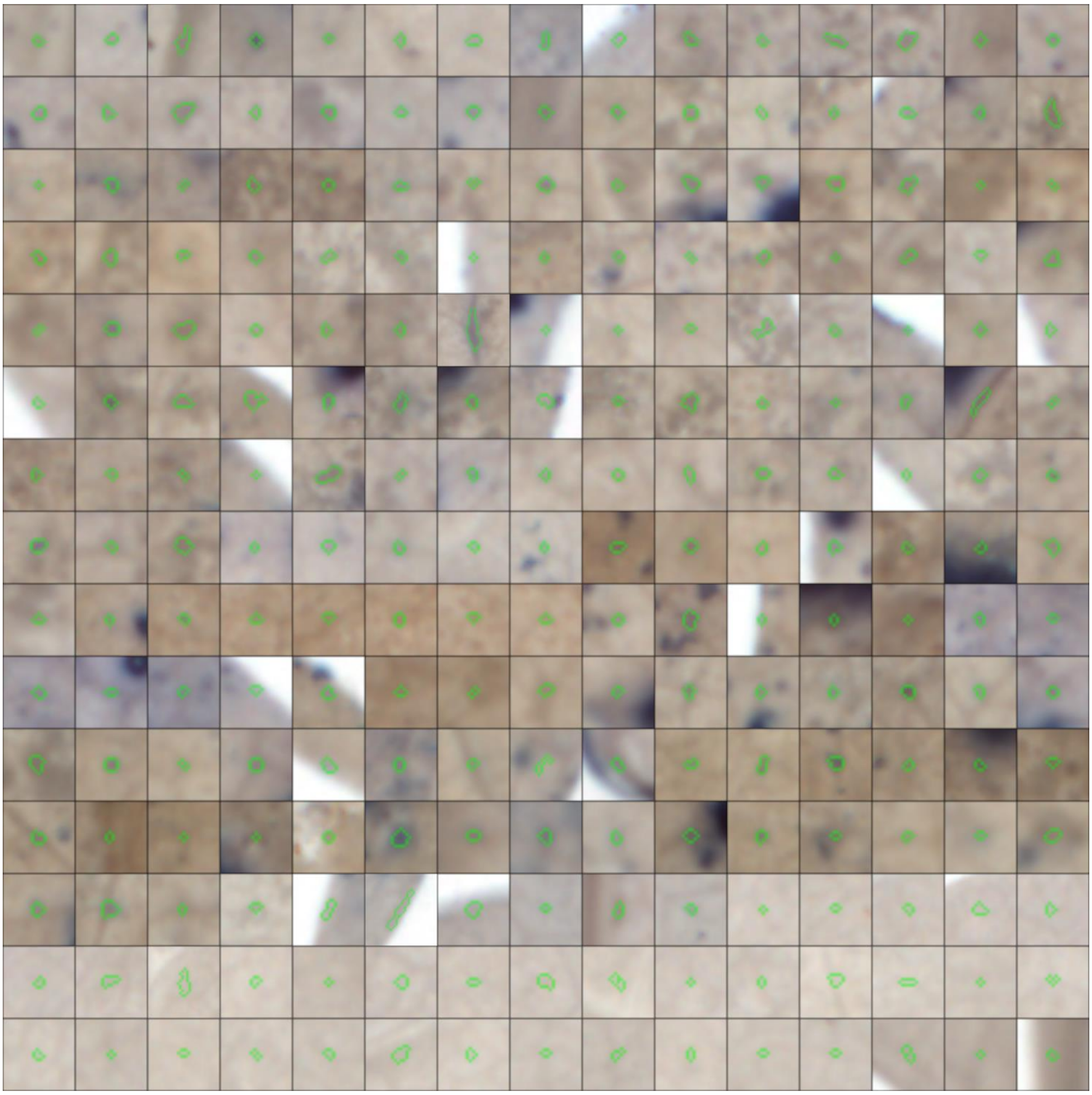

CLUSTER 12

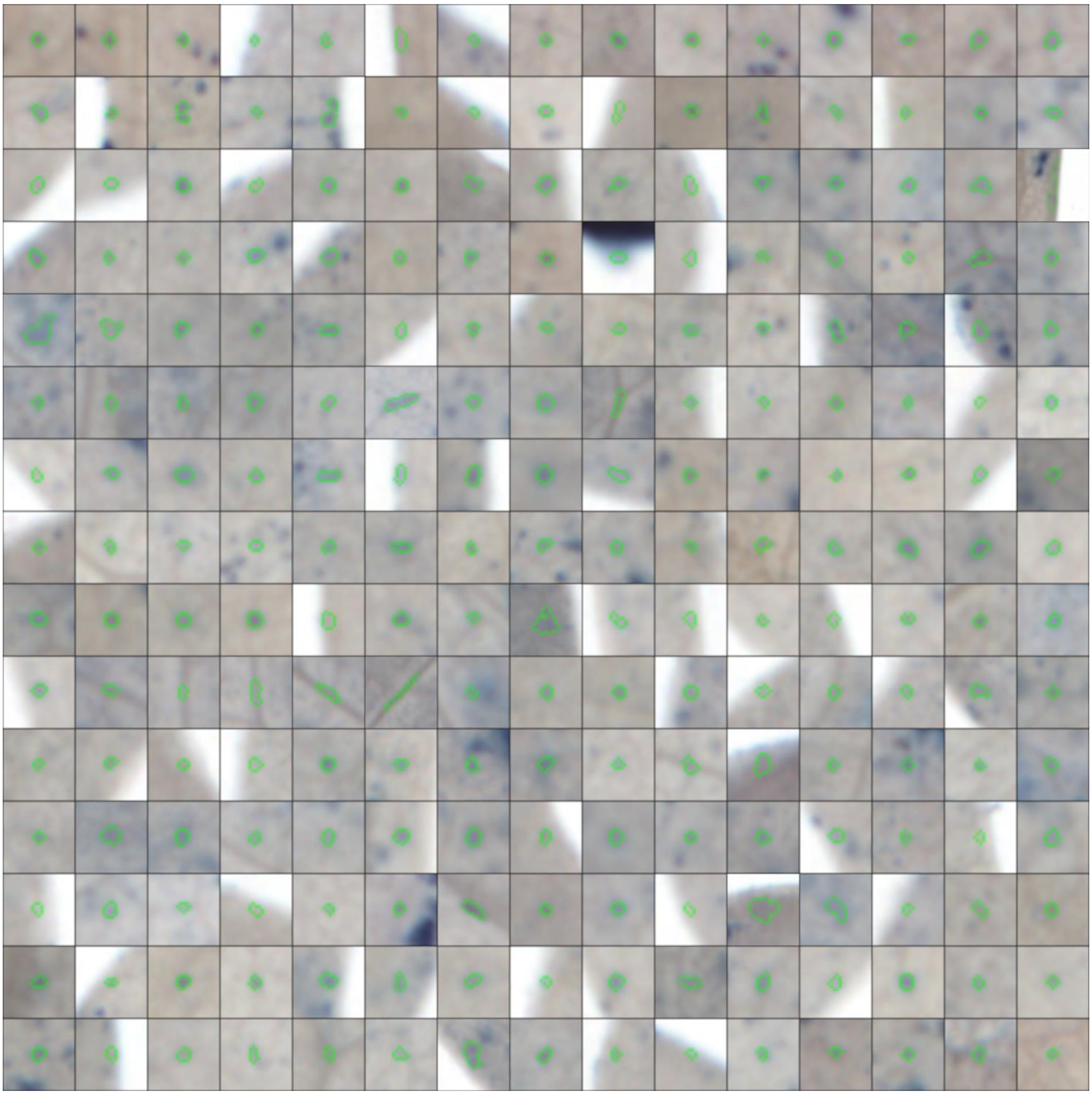

CLUSTER 13

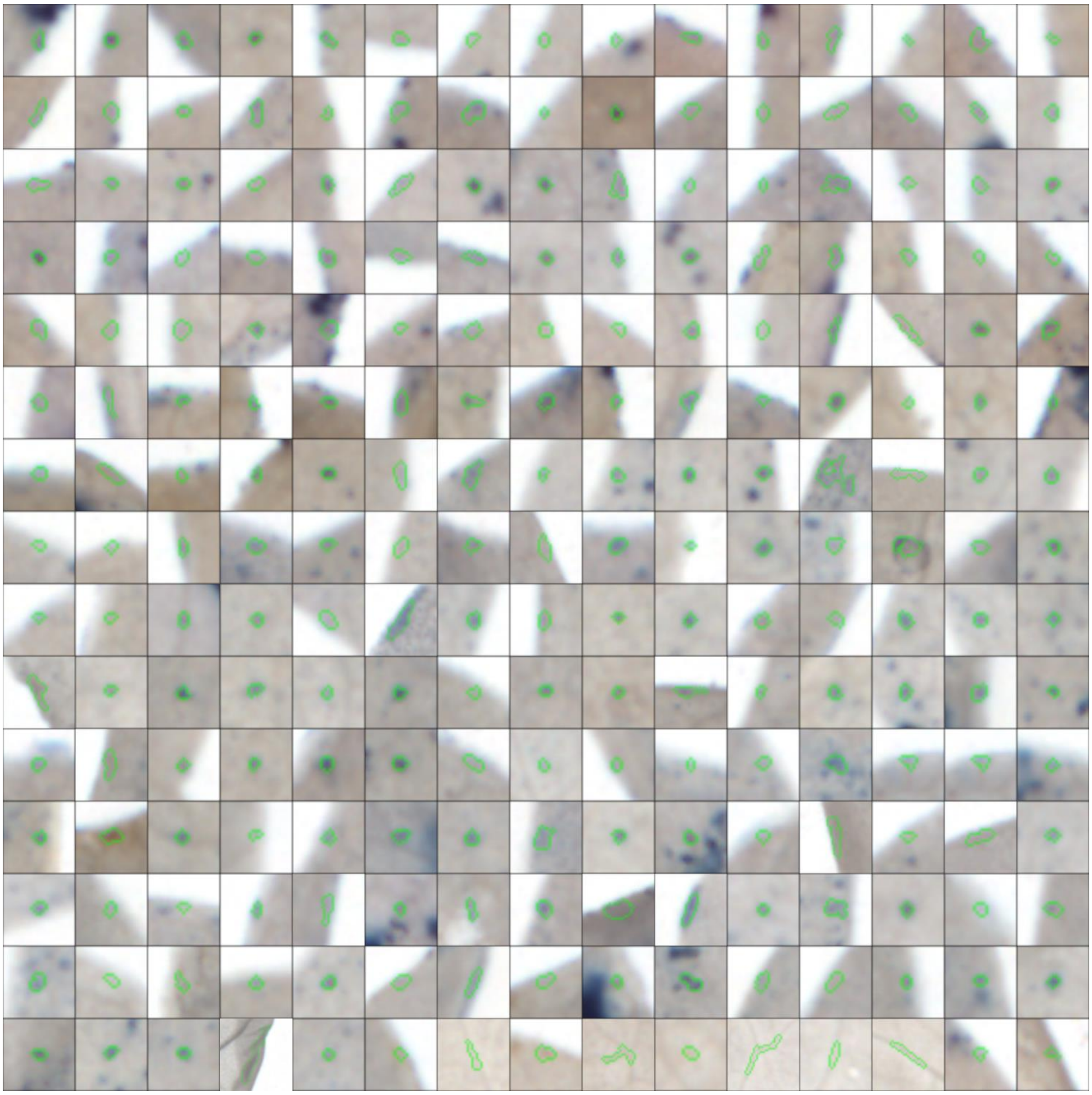

CLUSTER 14

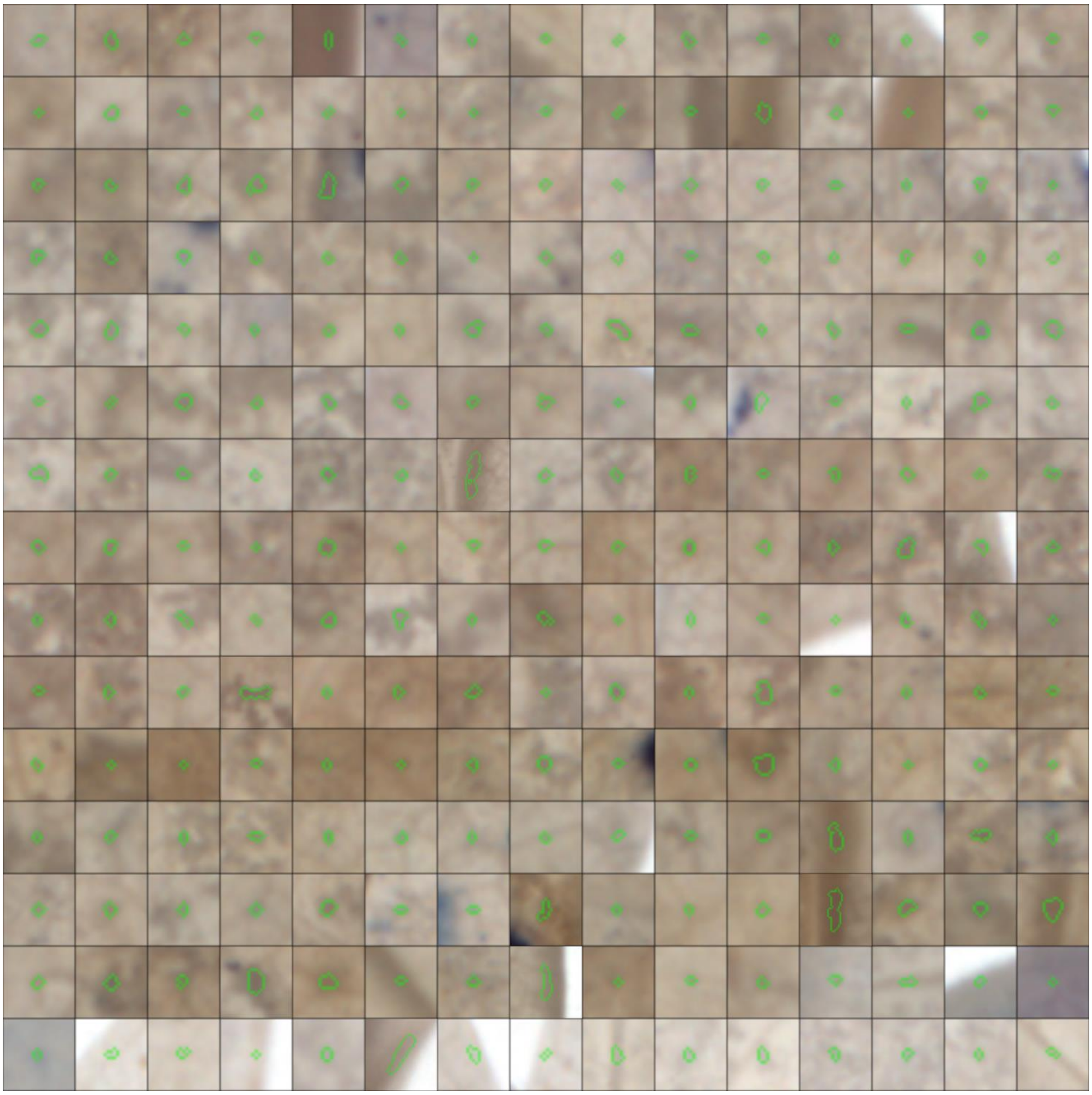

CLUSTER 15

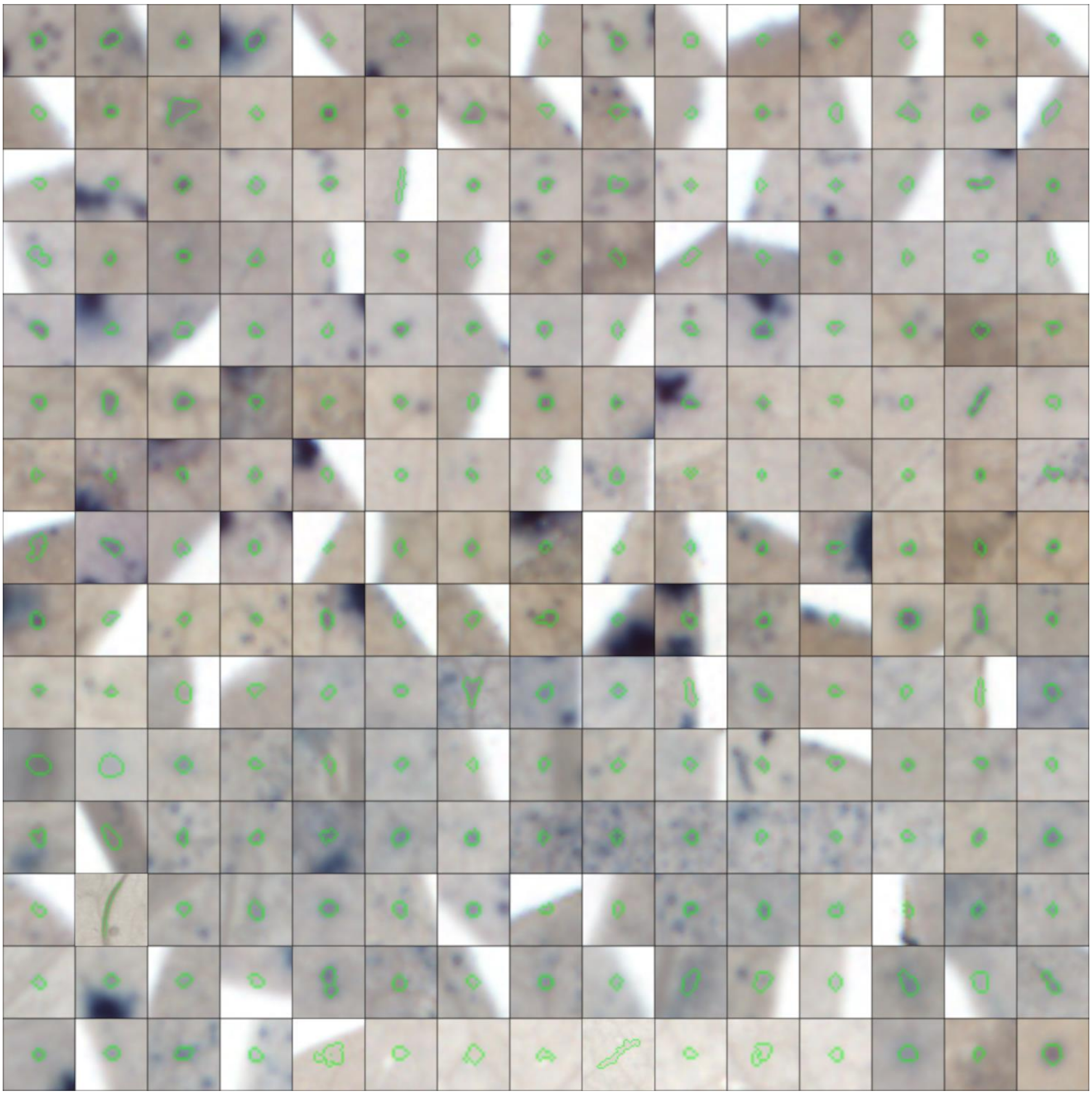

CLUSTER 16

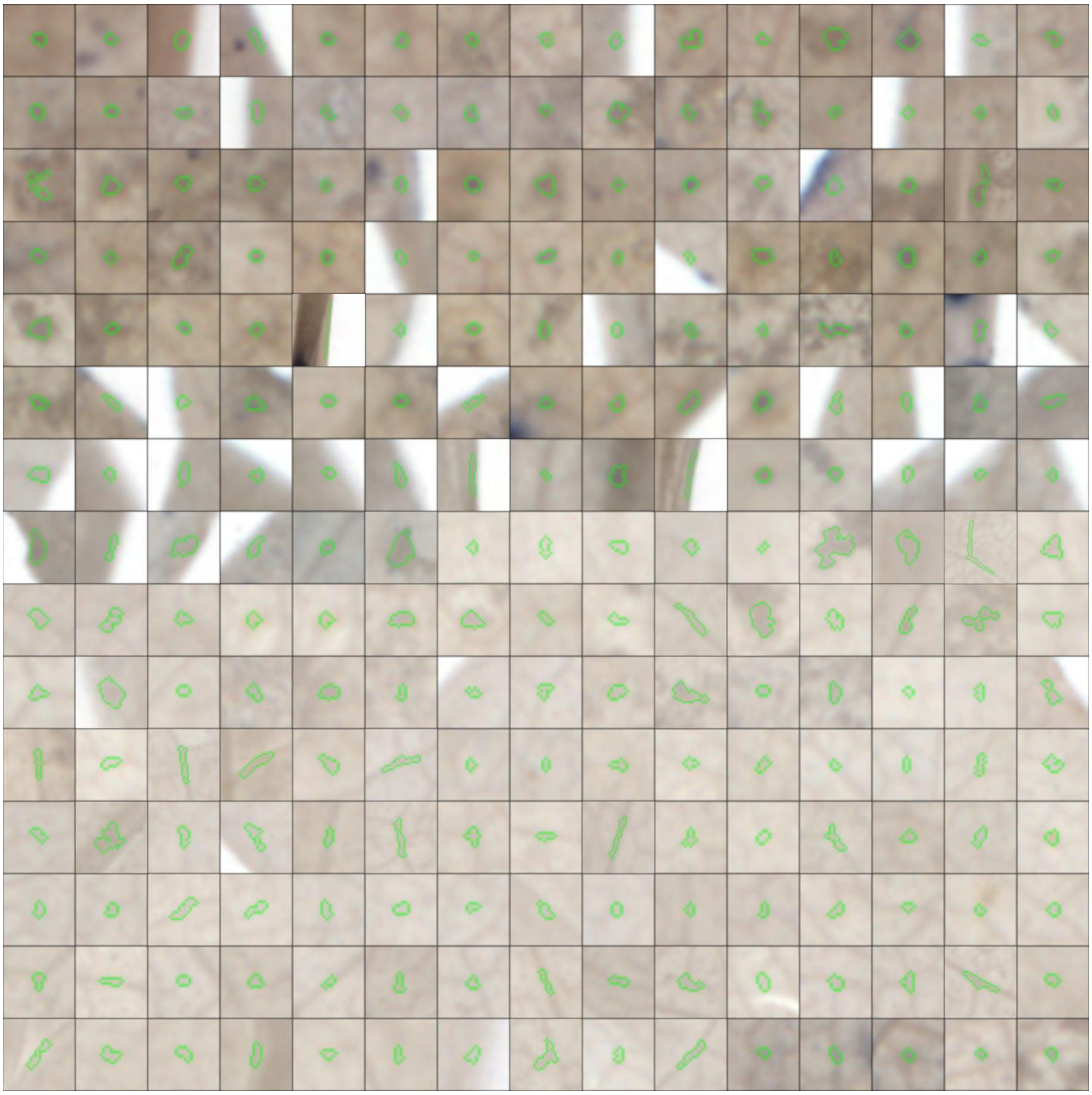

CLUSTER 17

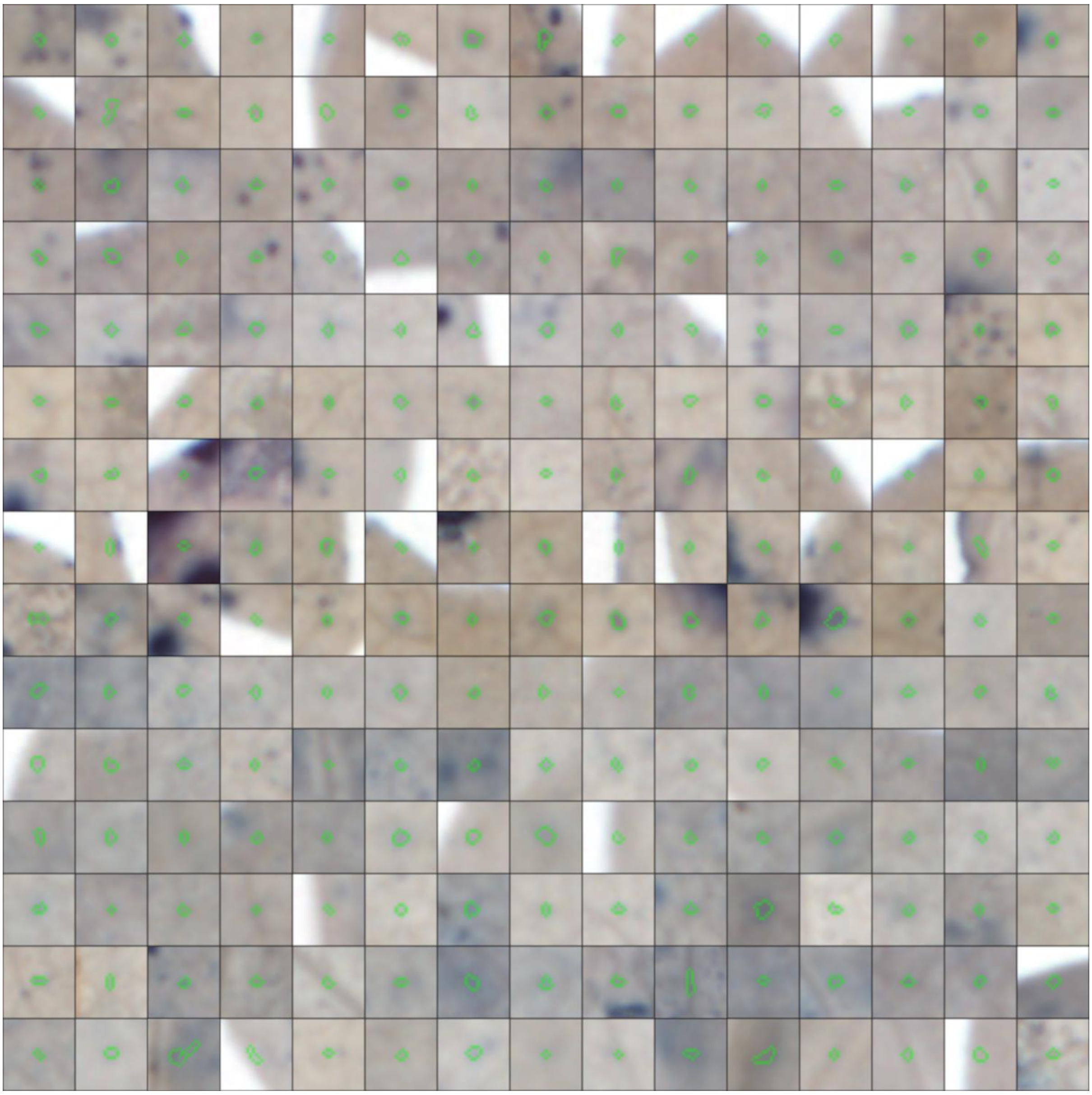

CLUSTER 18

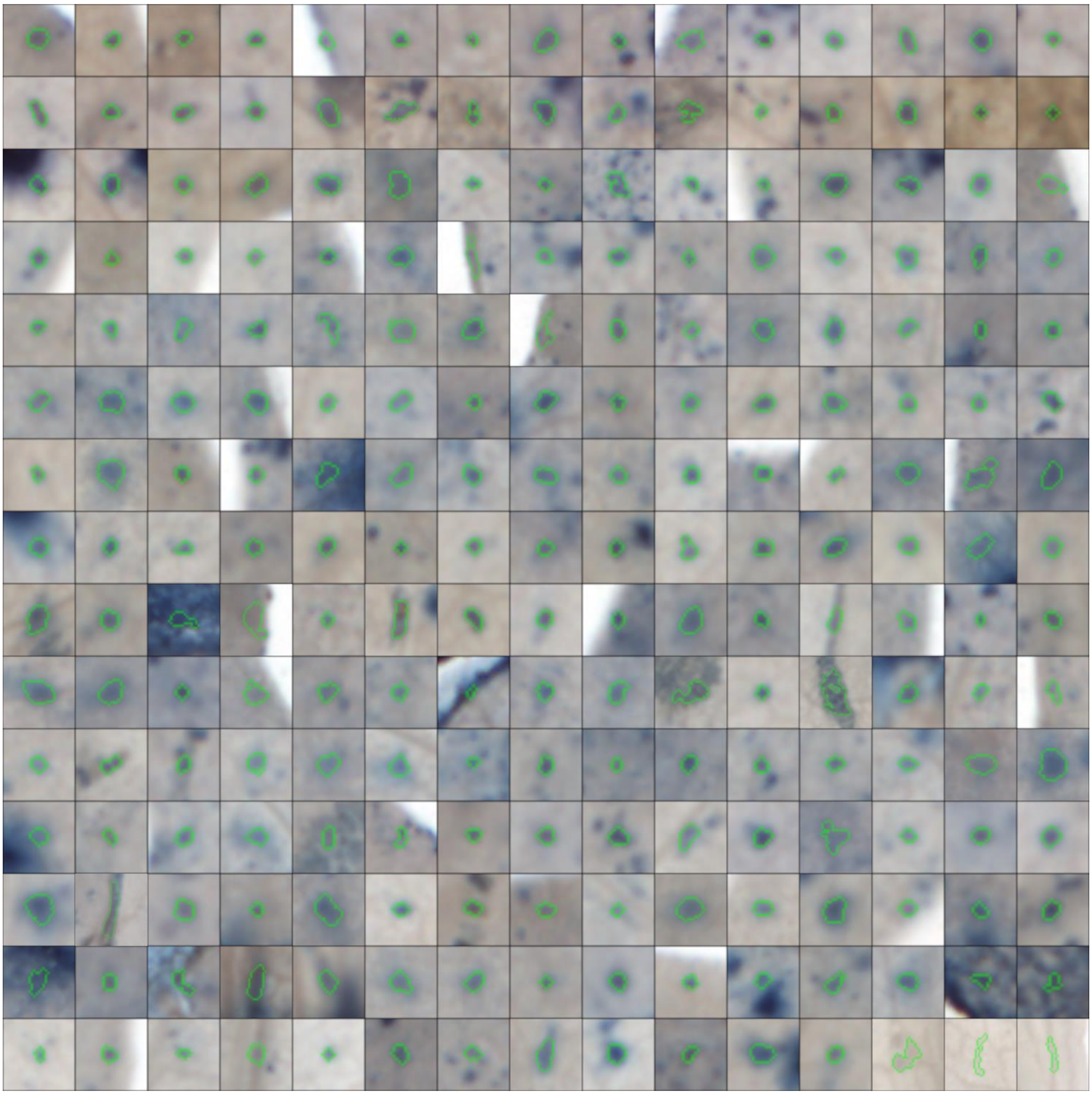

CLUSTER 19

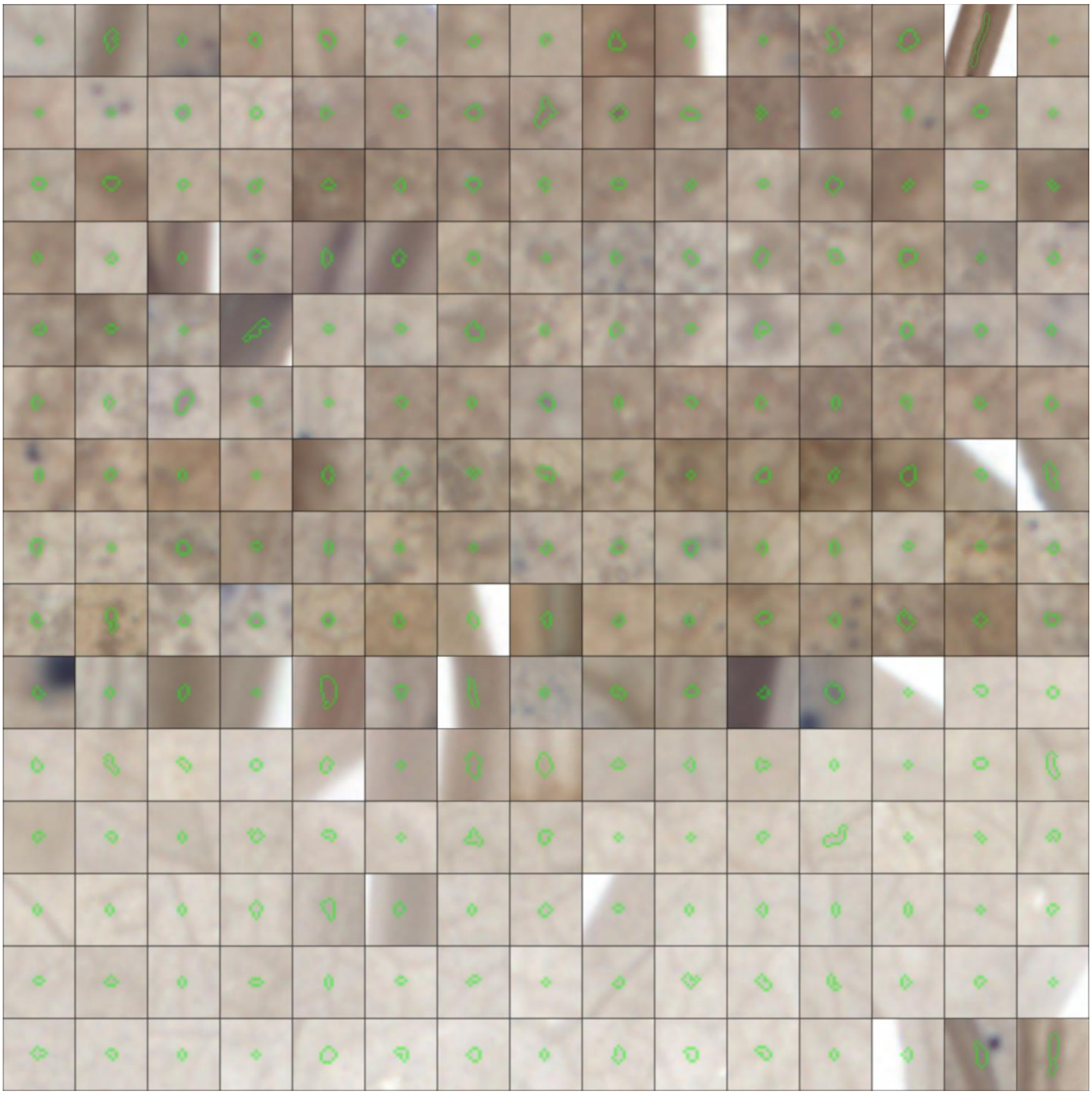

CLUSTER 20

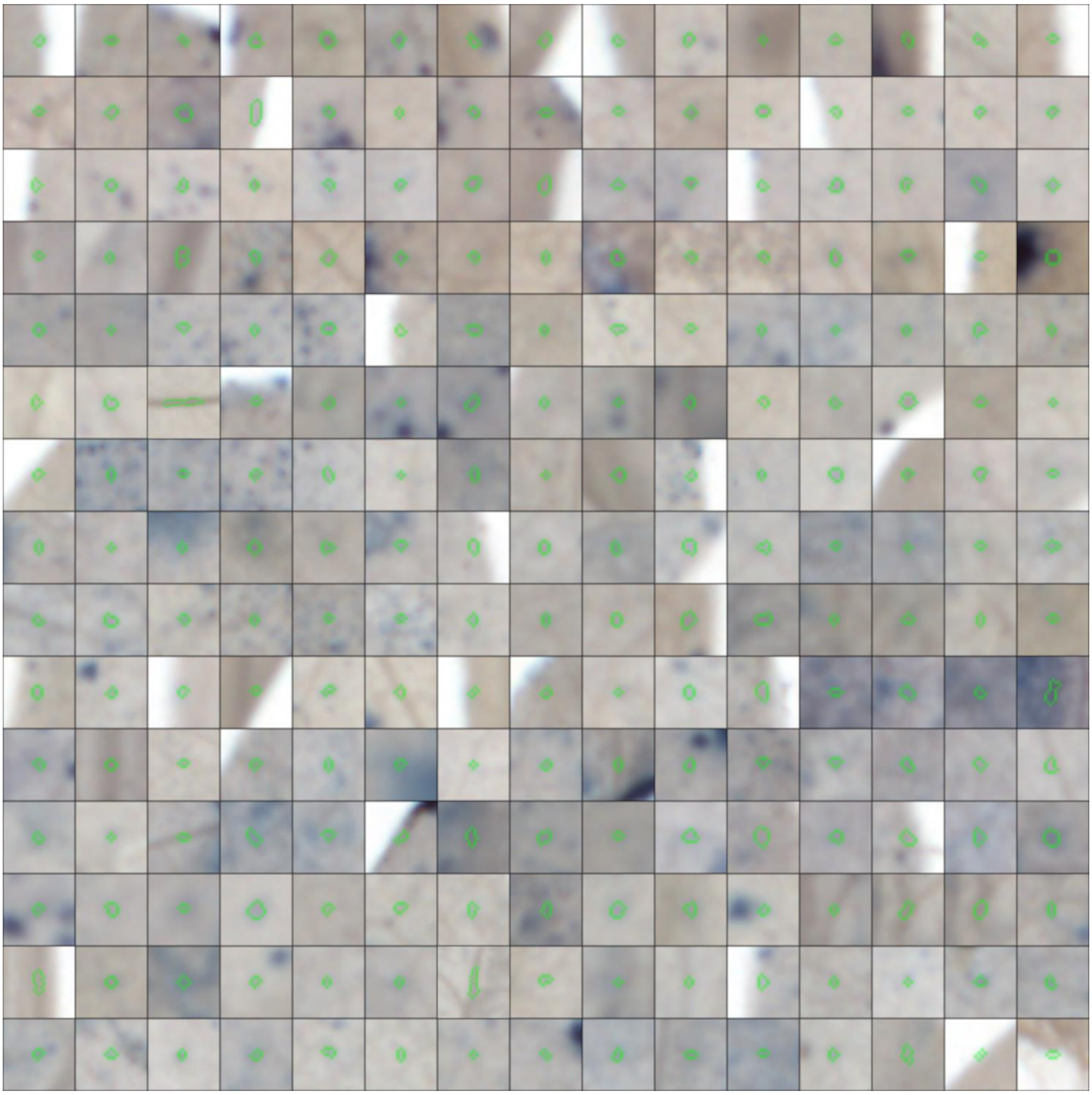

CLUSTER 21

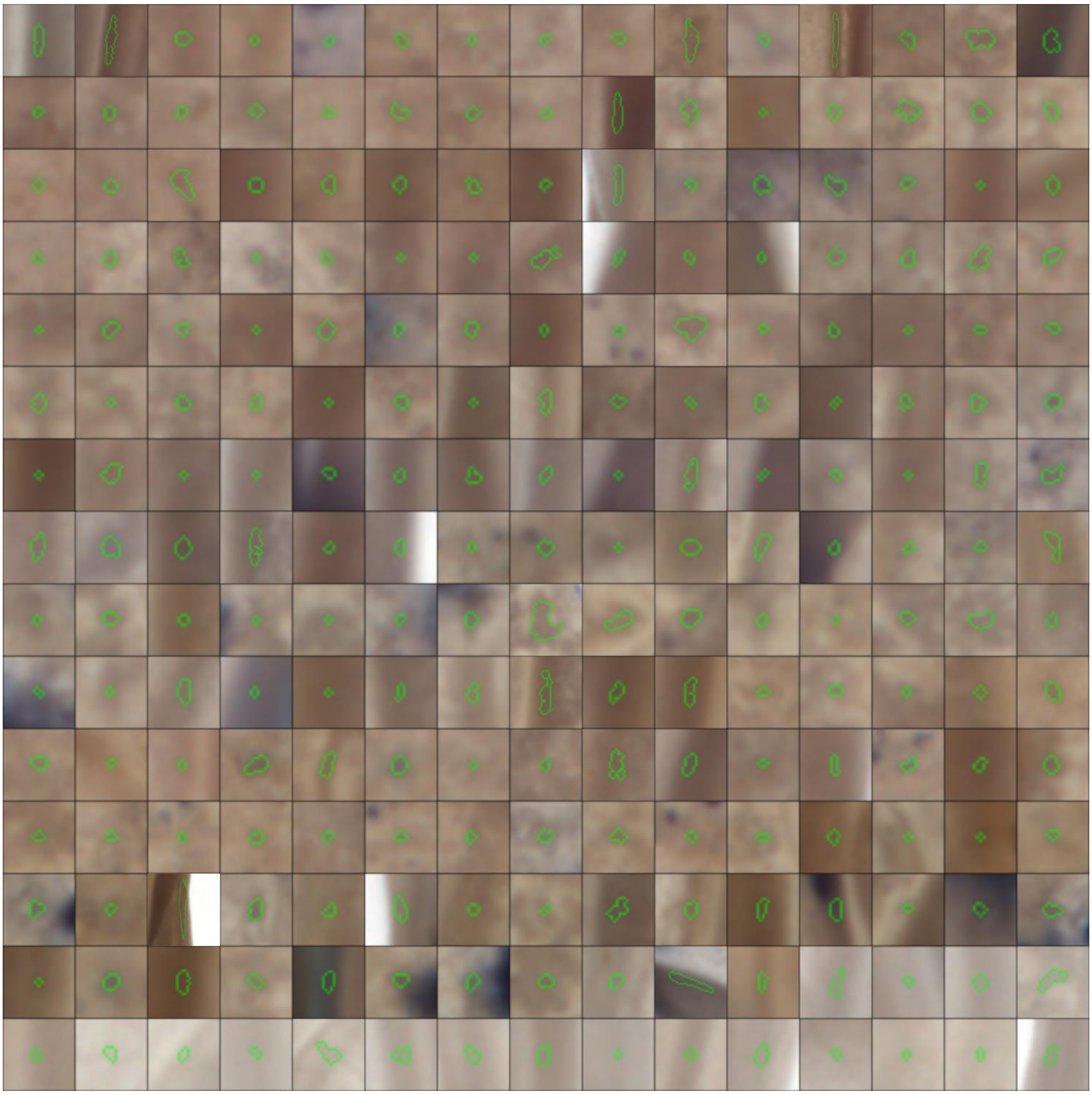

CLUSTER 22

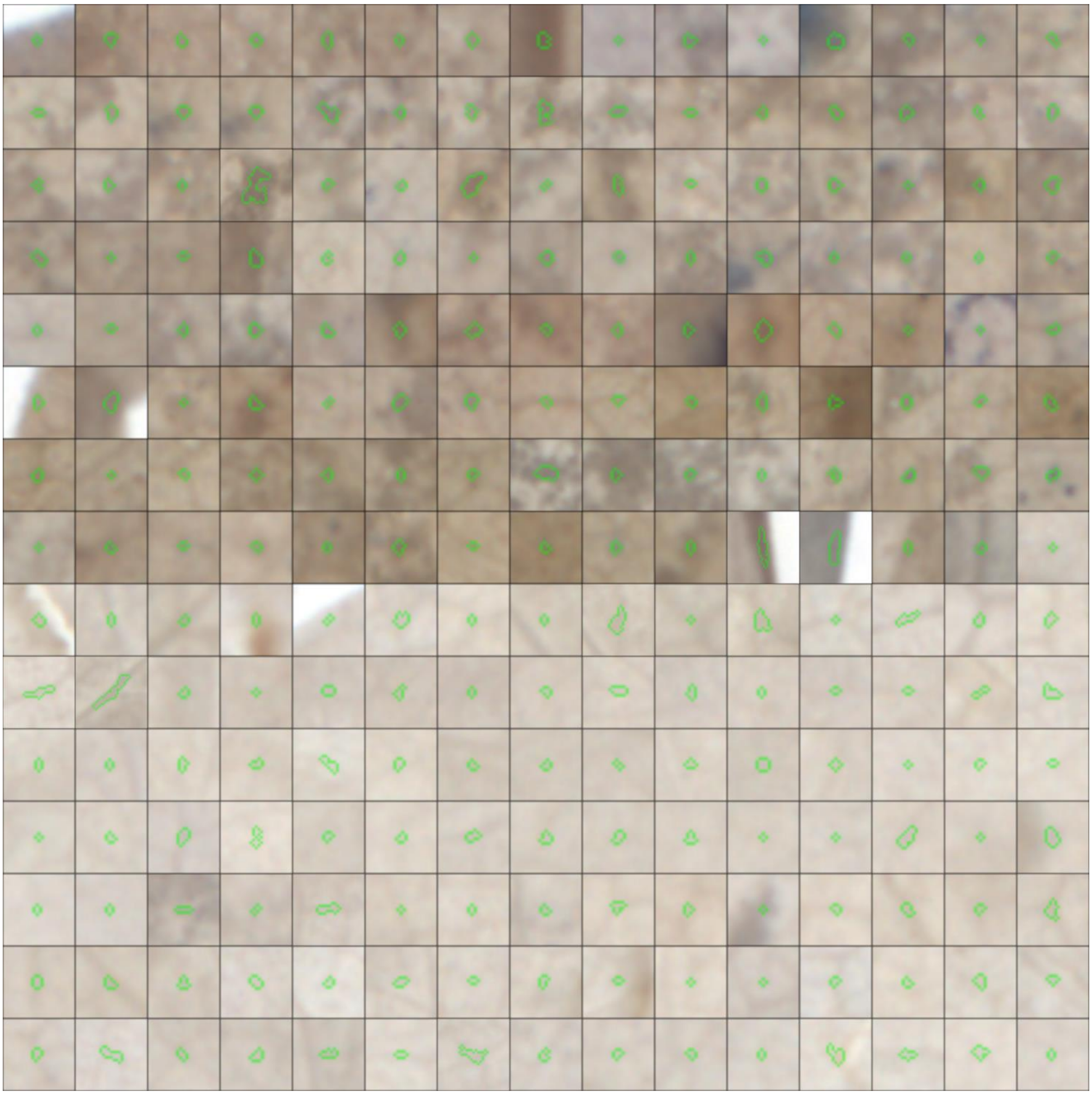

CLUSTER 23

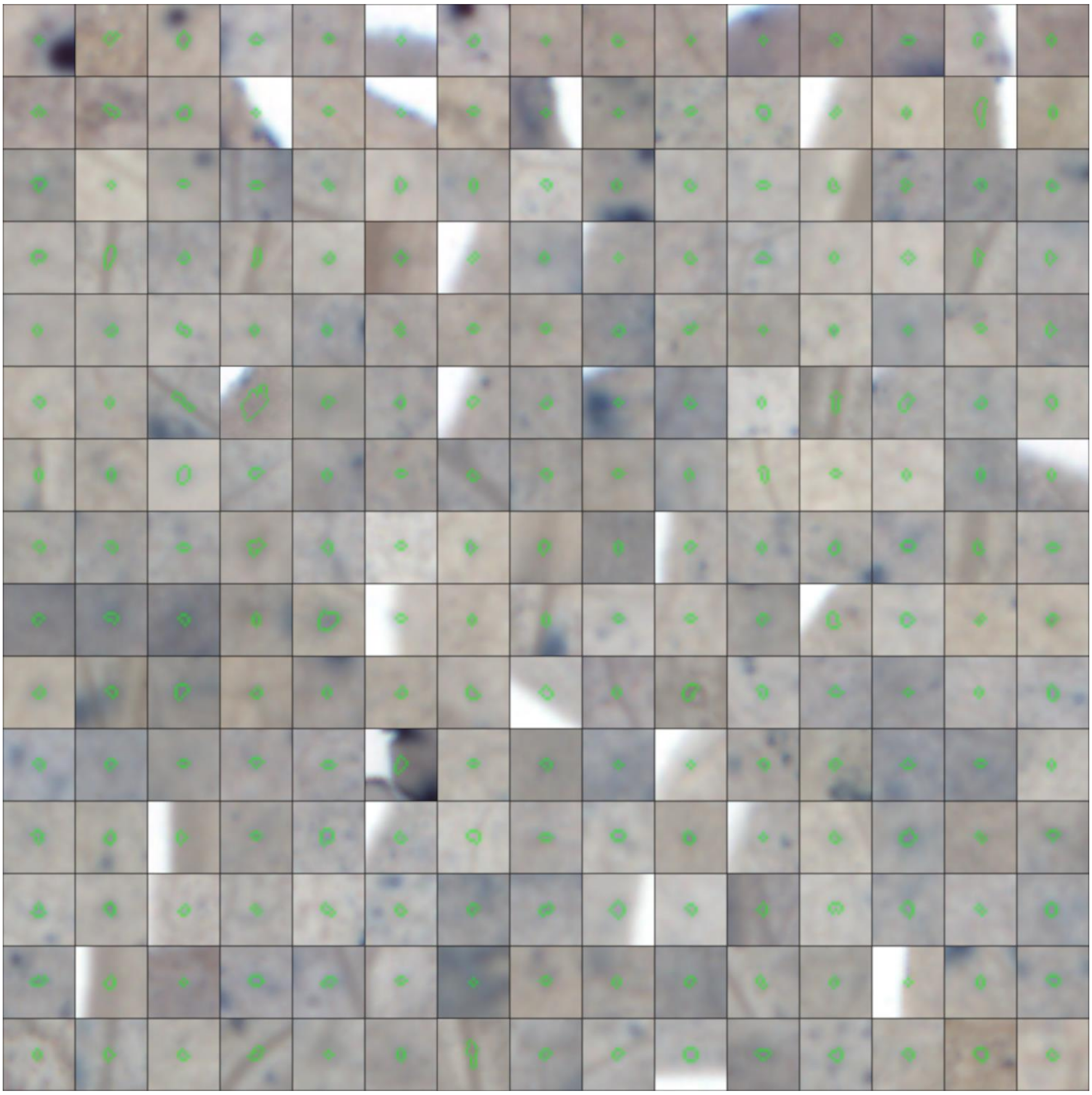

CLUSTER 24

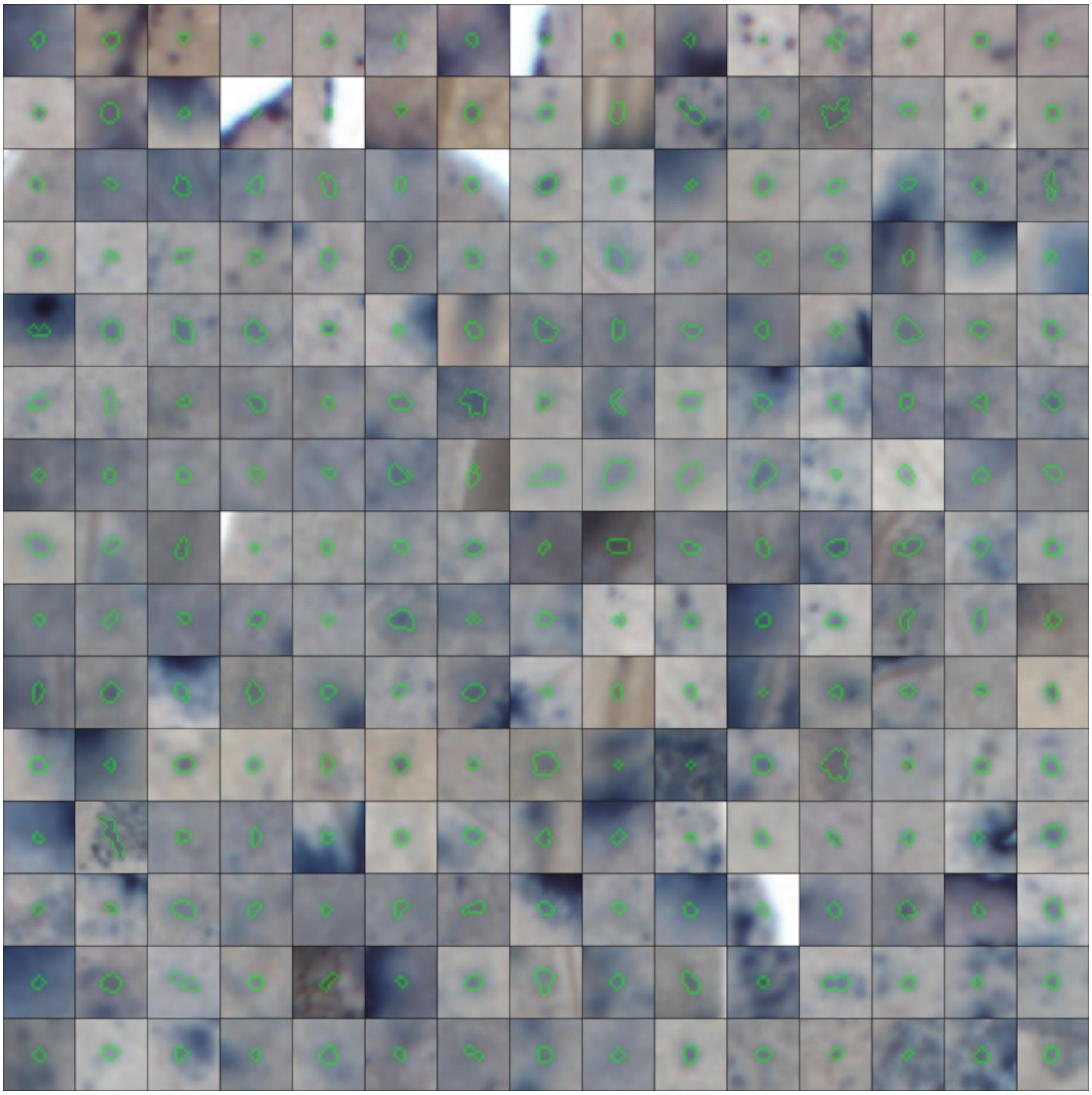

CLUSTER 25

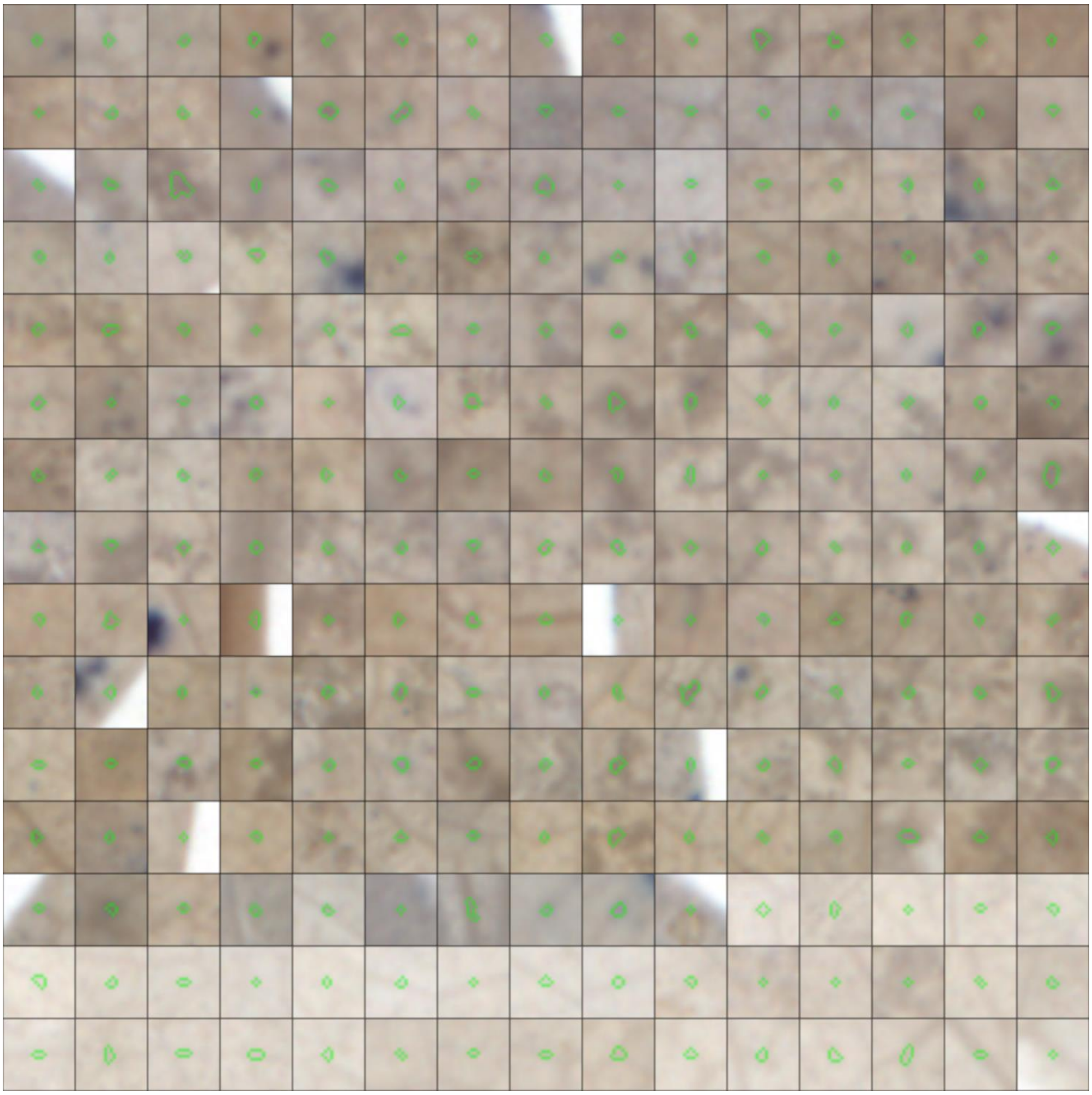

CLUSTER 26

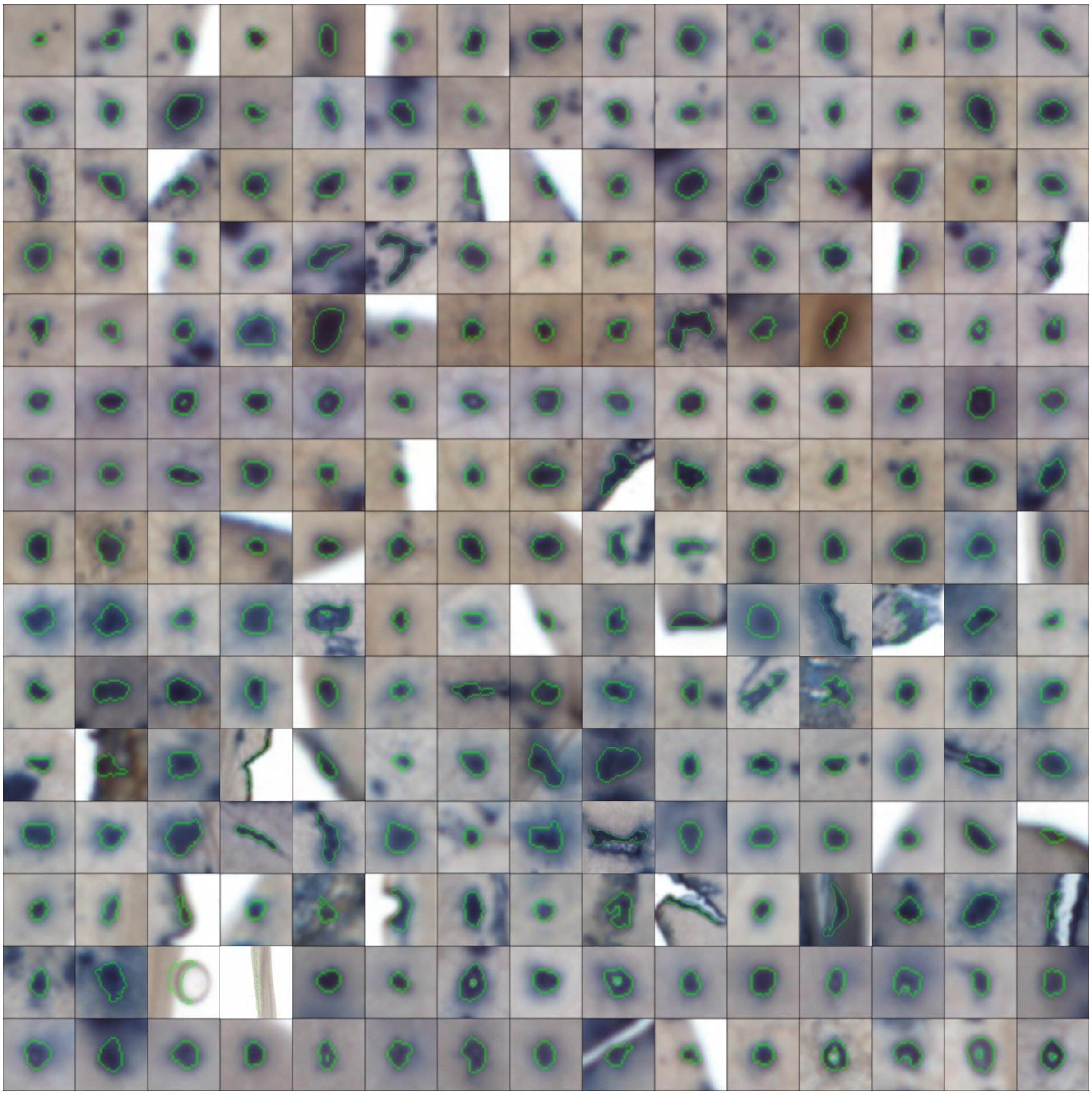

CLUSTER 27

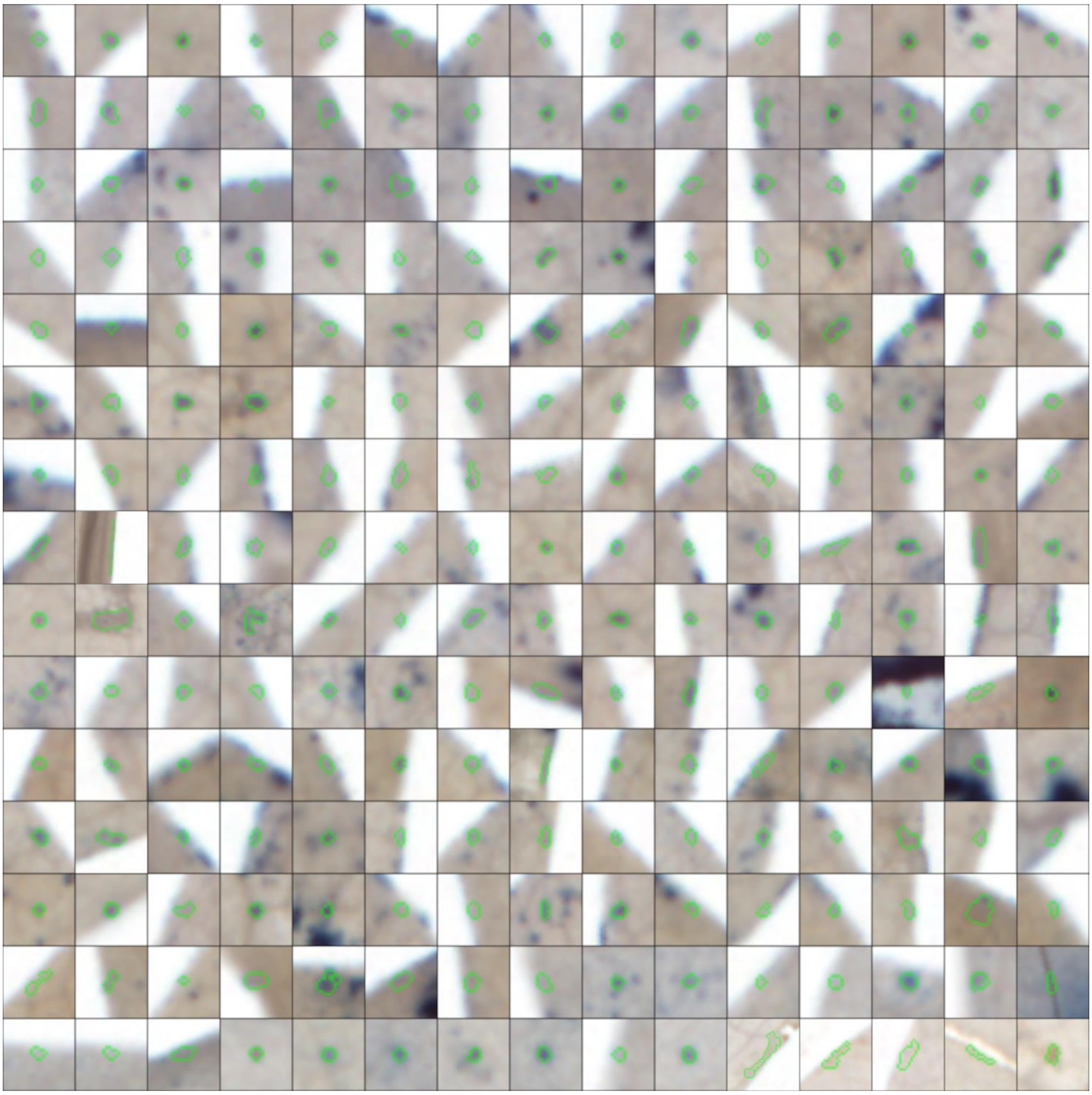

CLUSTER 28

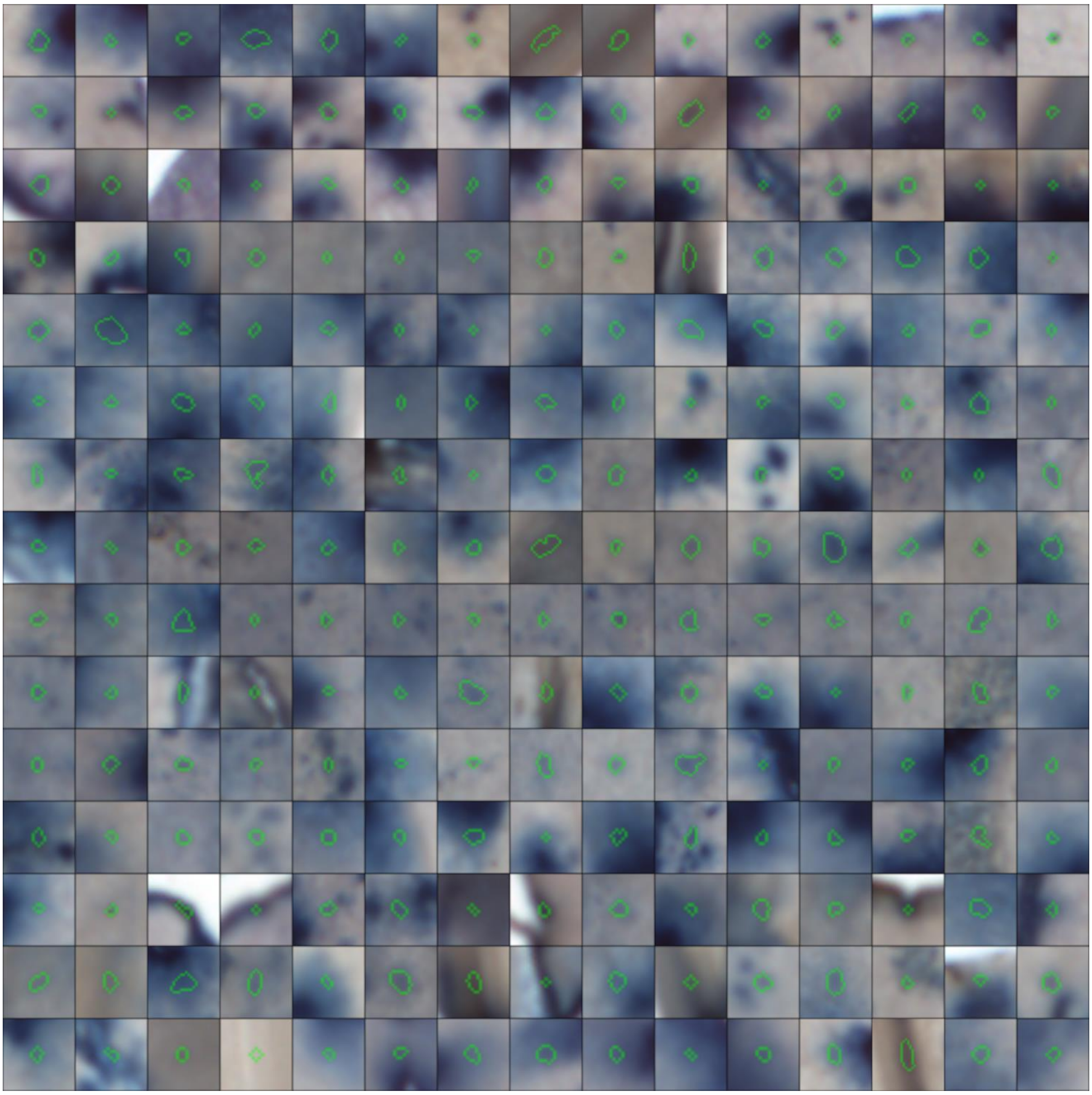

CLUSTER 29

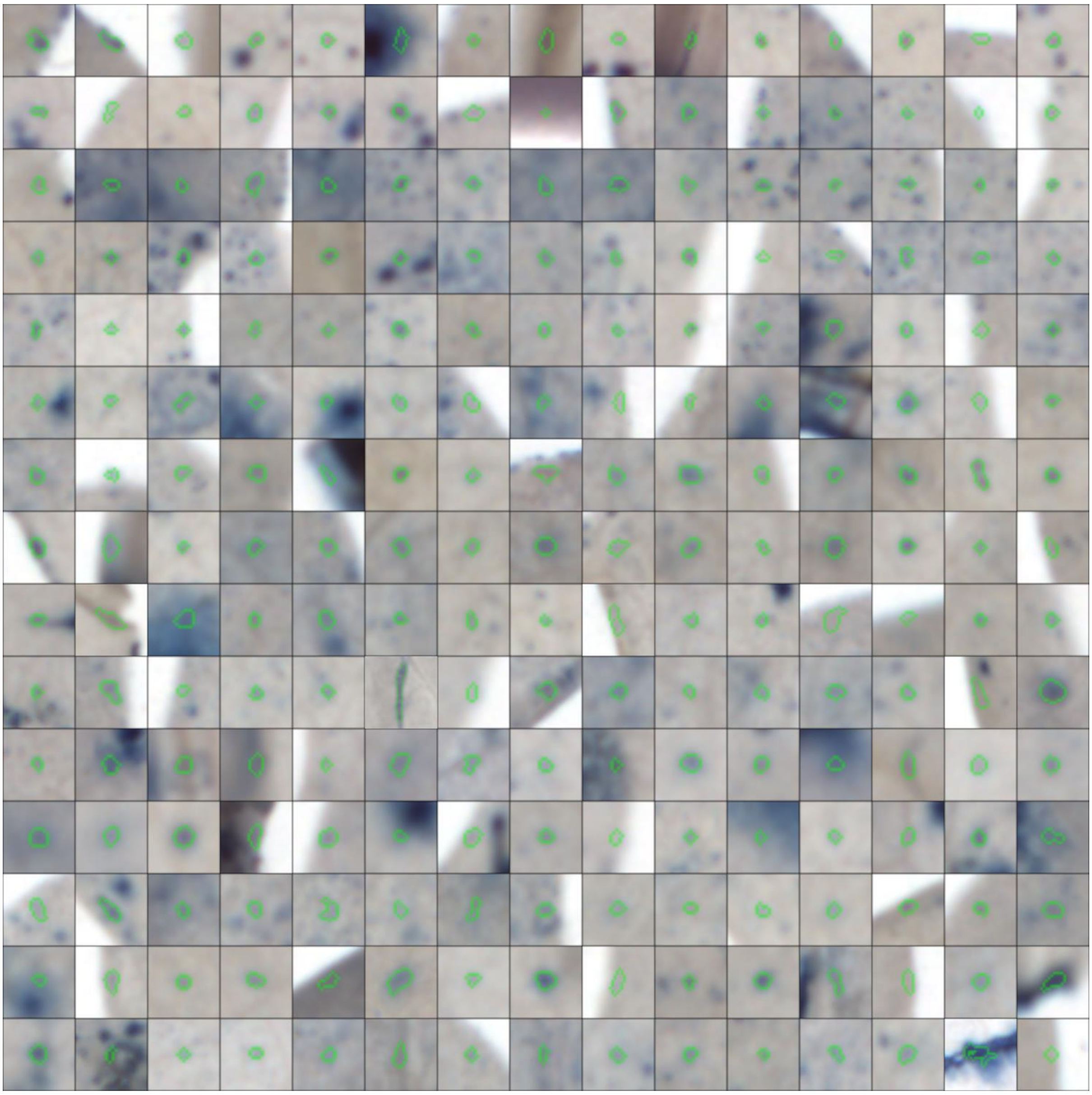

Supplement: Supplementary file 2 — Additional file 2. Lesion cluster classes. Lesion data were post-filtered and classified into 30 clusters, with each individual lesion in the text file assigned a number from 0 to 29 (true and false positive lesions). [file 13007_2020_605_MOESM2_ESM.pdf]
