# Supplementary material for: High-throughput method for detection and quantification of lesions on leaf scale based on trypan blue staining and digital image analysis
Source: Plant Methods. 2020 May 4;16:62. doi: 10.1186/s13007-020-00605-5 (PMC7197134; doi:10.1186/s13007-020-00605-5)
Supplement: Supplementary file 4 — Additional file 4. Differences between IMAGEJ and LiMu image analysis workflows. [file 13007_2020_605_MOESM4_ESM.docx]

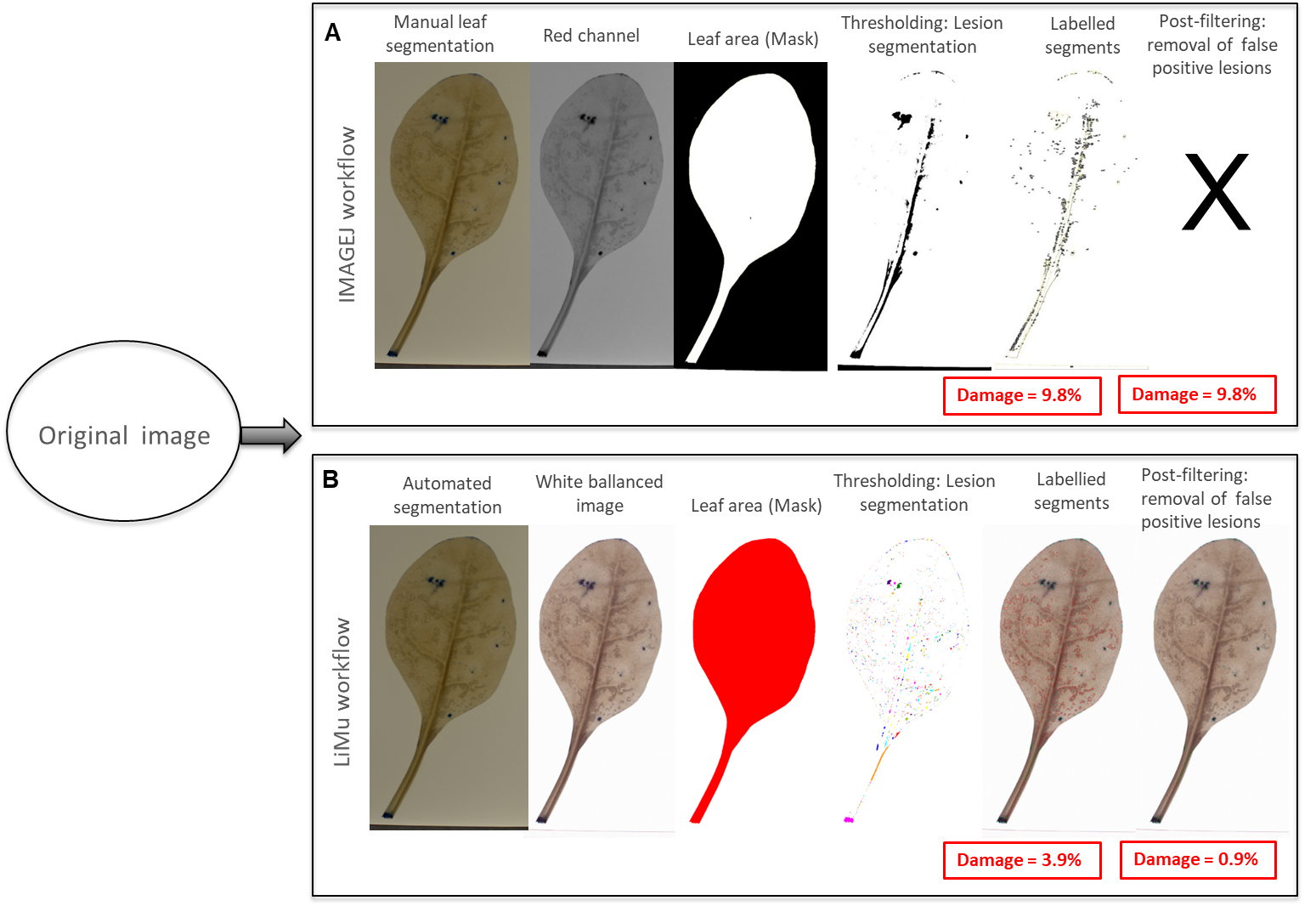


**Additional file 4**. Differences between IMAGEJ (A) and LiMu (B) image analysis workflows. An ”X” symbol represents a step omitted (lacking) in a workflow.
