# Supplementary material for: High-throughput method for detection and quantification of lesions on leaf scale based on trypan blue staining and digital image analysis
Source: Plant Methods. 2020 May 4;16:62. doi: 10.1186/s13007-020-00605-5 (PMC7197134; doi:10.1186/s13007-020-00605-5)
Supplement: Supplementary file 5 — Additional file 5. Cost-effectiveness of the proposed method. Laboratory set-up. [file 13007_2020_605_MOESM5_ESM.docx]

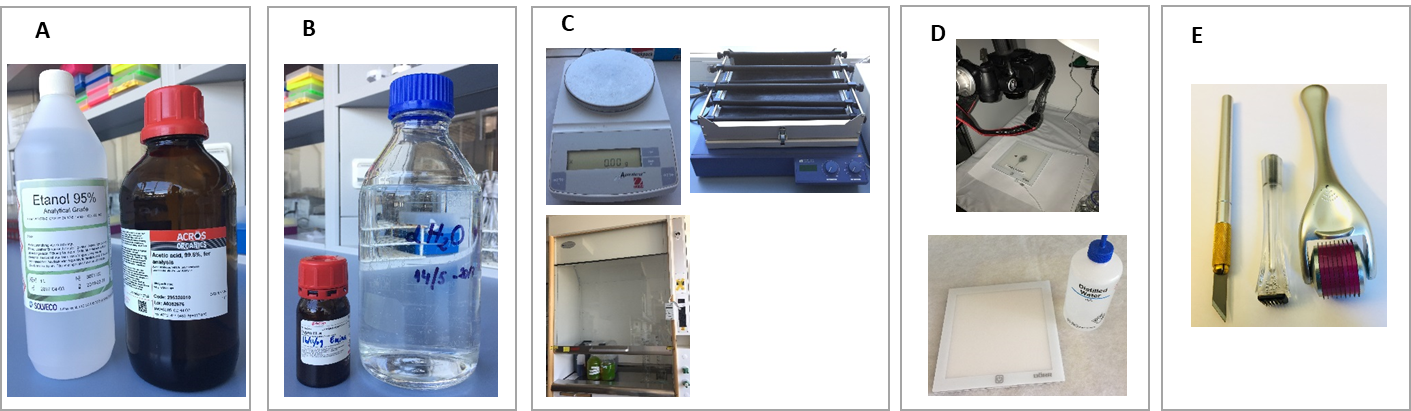


**Additional file 5.** Cost-effectiveness of the proposed method. Cost-effectiveness is achieved by using inexpensive chemicals for clearing (A), and staining (B), operating with common laboratory instruments (C), and image acquiring setting (D) placed in the same laboratory. Tools for inducing artificial damage to plant material were scalpel, mimicking cuts, and Derma stamp and Derma roller tools, mimicking dot-like lesions (E).
