# Supplementary material for: High-throughput method for detection and quantification of lesions on leaf scale based on trypan blue staining and digital image analysis
Source: Plant Methods. 2020 May 4;16:62. doi: 10.1186/s13007-020-00605-5 (PMC7197134; doi:10.1186/s13007-020-00605-5)
Supplement: Supplementary file 6 — Additional file 6. Visualisation of damaged and intact leaf tissue. Micrographs of damaged unstained leaf tissue and damaged and intact trypan blue-stained leaf tissue. [file 13007_2020_605_MOESM6_ESM.docx]

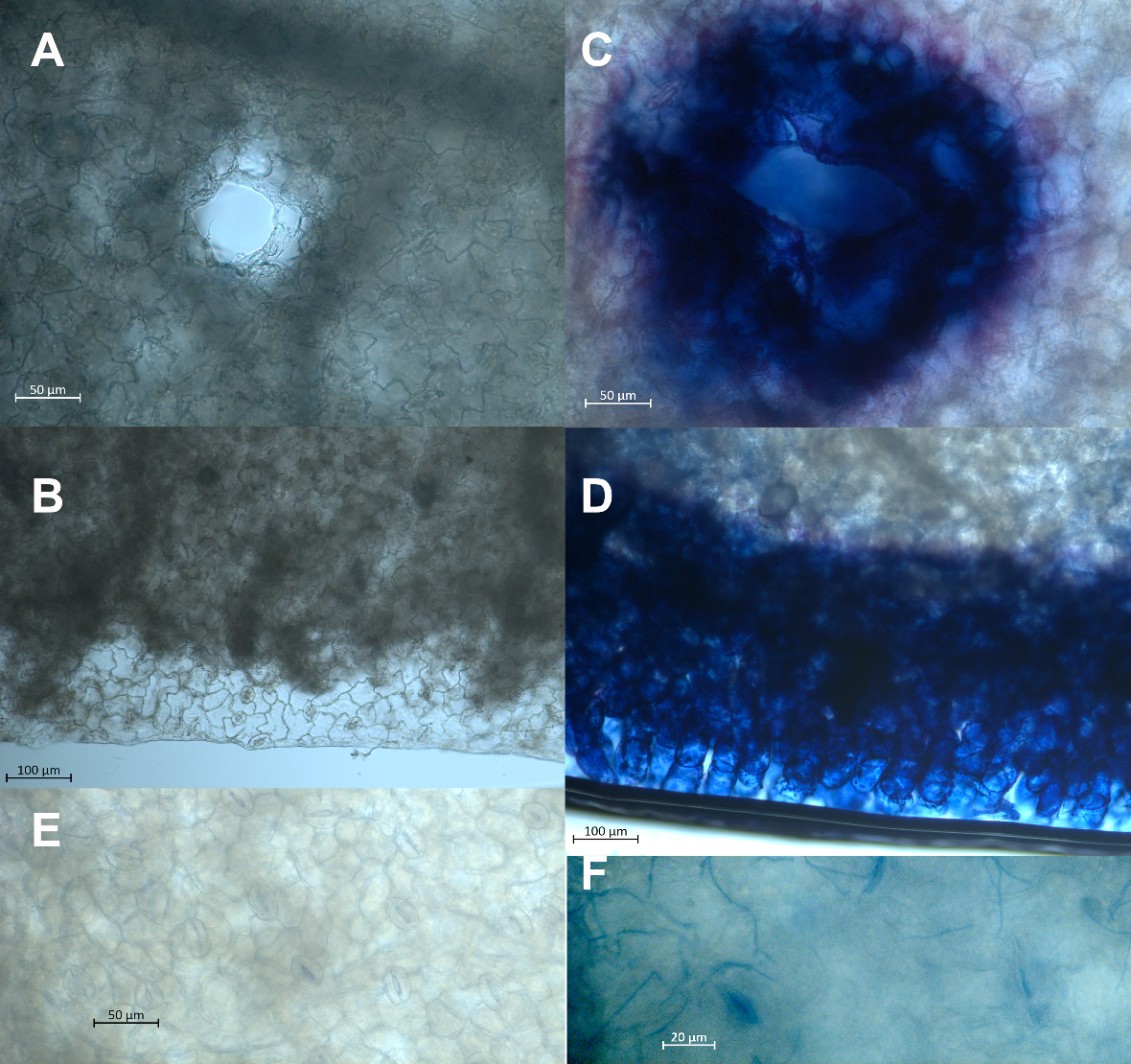


**Additional file 6.** Visualization of damaged and intact leaf tissue. Micrographs of

damaged unstained (A, B), damaged (C, D) and intact (E, F) trypan blue stained

spinach leaf tissue, observed using inverted Zeiss Axio Observer D1 microscope.
