# Supplementary material for: High-throughput method for detection and quantification of lesions on leaf scale based on trypan blue staining and digital image analysis
Source: Plant Methods. 2020 May 4;16:62. doi: 10.1186/s13007-020-00605-5 (PMC7197134; doi:10.1186/s13007-020-00605-5)
Supplement: Supplementary file 7 — Additional file 7. Statistical analysis results. Table S1. Results of LiMu image analysis using negative and positive control images. Table S2. Comparison between LiMu program, IMAGEJ and manual assessment of morphometric leaf and lesion parameters. Table S3. Comparison in lesion class quantification between LiMu program, IMAGEJ and manual assessment. Table S4. LiMu program results for leaf and lesion parameters, using the experimental image dataset. [file 13007_2020_605_MOESM7_ESM.docx]

**Table S1.** Different levels of standardized artificial damage (0 – 5) are induced to spinach leaves, mimicking different damage severity levels. Results of LiMu image analysis represent average values for negative (N=25) control images and each damage level for positive (N=5) control images. A non-parametric Kruskal-Wallis test was used, followed by Dunn′s post-hoc test. Significant differences between damage levels (p ≤ 0.05) found in the post-hoc test are indicated with asterisk (*).

| **Morphometric parameters** | **Damage levels** | | | | | | **Kruskal - Wallis test** | | | **Post-hoc test** | | | | | | | | | | | | | | |
| --- | --- | --- | --- | --- | --- | --- | --- | --- | --- | --- | --- | --- | --- | --- | --- | --- | --- | --- | --- | --- | --- | --- | --- | --- |
|  | **Median** | | | | | |  |  |  | **Damage levels** | | | | | | | | | | | | | | |
|  | **0** | **1** | **2** | **3** | **4** | **5** | ***Chi-squared*** | **df** | **p-value** | **0-1** | **0-2** | **1-2** | **0-3** | **1-3** | **2-3** | **0-4** | **1-4** | **2-4** | **3-4** | **0-5** | **1-5** | **2-5** | **3-5** | **4-5** |
| Leaf area (pixels) | 1562229 | 1362277 | 1215582 | 1357243 | 1309086 | 1239869 | 8.24 | 5 | 0.14 | 1.00 | 0.51 | 1.00 | 1.00 | 1.00 | 1.00 | 1.00 | 1.00 | 1.00 | 1.00 | 0.55 | 1.00 | 0.96 | 1.00 | 1.00 |
| Lesion area (pixels) | 13662 | 29165 | 22330 | 44788 | 51687 | 43677 | 22.03 | 5 | 5.2e-04 | 1.00 | 1.00 | 0.86 | 0.04* | 0.49 | 0.35 | 0.00* | 0.26 | 0.18 | 1.00 | 0.09 | 0.68 | 0.53 | 1.00 | 1.00 |
| Number of lesions | 102.5 | 598 | 263 | 667 | 593 | 306 | 25 | 5 | 1.4e-04 | 0.05 | 1.00 | 1.00 | 0.00* | 1.00 | 0.53 | 0.00* | 1.00 | 0.59 | 0.93 | 1.00 | 1.00 | 1.00 | 1.00 | 1.00 |
| Damage (%) | 0.93 | 1.72 | 1.76 | 3.72 | 3.95 | 3.44 | 27.92 | 5 | 3.8e-05 | 1.00 | 1.00 | 1.00 | 0.00* | 0.46 | 0.51 | 0.00* | 0.34 | 0.46 | 1.00 | 0.00* | 0.44 | 0.50 | 0.95 | 1.00 |

**Table S2.** Comparison between LiMu image analysis program, commonly used image analysis software ImageJ, and manual assessment of morphometric leaf and lesion parameters. Images used for comparisons between the three methods are positive control images (N=10), and randomly chosen images from experimental data set (N=10). Morphometric parameters such as leaf area, lesion area, and percentage of leaf damage were compared. A non-parametric Friedman′s test was used, followed by Dunn′s post-hoc test. Significant differences (p ≤ 0.05) found in the post-hoc test are indicated with asterisk (*).

| **Positive comtrol images (N=10)** | **Methods** | | | | | | **Friedman test** | | | **Post-hoc test** | | |
| --- | --- | --- | --- | --- | --- | --- | --- | --- | --- | --- | --- | --- |
|  | **Median** | | | **Interquartile range (IQR)** | | |  | | | **Methods** | | |
|  | **LiMu** | **ImageJ** | **Manual** | **LiMu** | **ImageJ** | **Manual** | ***Chi-squared*** | **df** | **p-value** | **LiMu-ImageJ** | **LiMu-Manual** | **ImageJ-Manual** |
| Leaf area (pixels) | 1258998 | 1257363 | 1263459 | 177869.2 | 180036.8 | 177135.2 | 5.6 | 2 | 0.06 | 0.076 | 0.147 | 0.655 |
| Lesion area (pixels) | 22643.5 | 16256 | 28285.5 | 11898.7 | 8641.2 | 11247.7 | 18.2 | 2 | 1.11e-5 | 0.028* | 0.073 | 6.45e-05* |
| Damage (%) | 1.758 | 1.313 | 2.126 | 0.586 | 0.437 | 0.756 | 18.2 | 2 | 1.11e-5 | 0.028* | 0.074 | 6.45e-05* |
| **Experimental images (N=10)** | **LiMu** | **ImageJ** | **Manual** | **LiMu** | **ImageJ** | **Manual** | ***Chi-squared*** | ***df*** | ***p-value*** | **LiMu-ImageJ** | **LiMu-Manual** | **ImageJ-Manual** |
| Leaf area (pixels) | 1636461 | 1629291 | 1635874 | 381507 | 377847.5 | 371651 | 13.818 | 2 | 9.98e-4 | 0.002* | 0.669 | 0.005* |
| Lesion area (pixels) | 13550 | 10442 | 13909 | 9645.5 | 10732.5 | 16132.0 | 11.091 | 2 | 3.9e-3 | 0.011* | 0.831 | 0.008* |
| Damage (%) | 0.914 | 0.729 | 1.117 | 0.347 | 0.661 | 1.010 | 11.091 | 2 | 3.9e-3 | 0.011* | 0.831 | 0.008* |

**Table S3:** Regions of interest (ROI′s = lesions) are measured on a binary image of artificially damaged and stained spinach leaf. Total quantified ROI′s are based on their area classified in three classes, namely microlesions, mesolesions, and macrolesions. Result of comparison in lesion classes’ quantification between LiMu program, a commonly used Image analysis software ImageJ, and manual assessment is conducted. Images used for comparisons are positive control images (N=10), and randomly chosen images from experimental data set (N=10). Significant differences (p ≤ 0.05) between the three methods found in the post-hoc test are indicated with asterisk (*).

| **Positive control images (N=10)** | **Methods** | | | | | | **Friedman test** | | | **Post-hoc test** | | |
| --- | --- | --- | --- | --- | --- | --- | --- | --- | --- | --- | --- | --- |
|  | **Median** | | | **Interquartile range (IQR)** | | |  |  |  |  |  |  |
|  | **LiMu** | **ImageJ** | **Manual** | **LiMu** | **ImageJ** | **Manual** | ***Chi-squared*** | **df** | **p-value** | **LiMu-ImageJ** | **LiMu-Manual** | **ImageJ-Manual** |
| Total lesion count | 302 | 106.5 | 98.5 | 268.5 | 40.75 | 45.75 | 15.43 | 2 | 4.4e-4 | 0.001* | 0.002* | 0.823 |
| Microlesions | 3 | 6.5 | 0 | 3.5 | 3.5 | 0 | 17.21 | 2 | 1.8e-4 | 0.093 | 0.037* | 0.0001* |
| Mesolesions | 281.5 | 74.5 | 48.5 | 268.5 | 35.5 | 25 | 16.8 | 2 | 2.2e-4 | 0.014* | 0.0001* | 0.179 |
| Macrolesions | 25.5 | 21 | 42.5 | 14 | 10.5 | 19 | 18.2 | 2 | 1.1e-4 | 0.073 | 0.027* | 6.45e-05* |
| **Experimental images (N=10)** | **LiMu** | **ImageJ** | **Manual** | **LiMu** | **ImageJ** | **Manual** | ***Chi-squared*** | **df** | **p-value** | **LiMu-ImageJ** | **LiMu-Manual** | **ImageJ-Manual** |
| Total lesion count | 306 | 24 | 35 | 162.5 | 9.5 | 9 | 22 | 2 | 1.67e-05 | 8.18e-06* | 0.038* | 0.038* |
| Microlesions | 3 | 1 | 0 | 3.5 | 1.5 | 0 | 11.4 | 2 | 3.3e-03 | 0.2 | 0.004* | 0.11 |
| Mesolesions | 291 | 15 | 27 | 155 | 13 | 8.5 | 22 | 2 | 1.67e-05 | 8.17e-06* | 0.038* | 0.038* |
| Macrolesions | 10 | 8 | 10 | 7 | 3.5 | 2.5 | 16.8 | 2 | 2.24e-04 | 0.021* | 0.2 | 0.0003* |

| **Morphometric parameters** | **Methods** | | | | **Wilcoxon test** | |
| --- | --- | --- | --- | --- | --- | --- |
|  | **Median** | | **Interquartile range (IQR)** | |  |  |
|  | **Swiss chard** | **Spinach** | **Swiss chard** | **Spinach** | **W** | **p-value** |
| Lesion area (px) | 3250 | 8153 | 3112.5 | 5377.0 | 5374 | < 2.2e-16 **** |
| Lesion area (%) | 0.296 | 0.579 | 0.231 | 0.377 | 7092 | < 2.2e-16 **** |
| Microlesions (%) | 0.00 | 0.336 | 0.00 | 0.857 | 14320 | 2.141e-08 **** |
| Mesolesions (%) | 95.652 | 96.207 | 4.286 | 2.474 | 18120 | 0.1222 |
| Macrolesions (%) | 3.846 | 3.125 | 4.074 | 2.385 | 22723 | 0.01428* |

**Table S4:** Results for leaf and lesion parameters for spinach and Swiss chard vegetables using LiMu program. Differences between Swiss chard and spinach in lesion area (px), damage (%) and lesion classes between two species are shown. A non-parametric Wilcoxon test is used for mean values comparisons between two species, and significant differences (p ≤ 0.05) between species are indicated with asterisk (*).

* p ≤ 0.05, ** p ≤ 0.01, *** p ≤ 0.001, **** p ≤ 0.0001
